# A New Basal Hadrosauroid Dinosaur (Dinosauria: Ornithopoda) with Transitional Features from the Late Cretaceous of Henan Province, China

**DOI:** 10.1371/journal.pone.0098821

**Published:** 2014-06-05

**Authors:** Hai Xing, Deyou Wang, Fenglu Han, Corwin Sullivan, Qingyu Ma, Yiming He, David W. E. Hone, Ronghao Yan, Fuming Du, Xing Xu

**Affiliations:** 1 College of Earth Sciences and Resources, China University of Geosciences, Beijing, China; 2 Key Laboratory of Vertebrate Evolution and Human Origins of Chinese Academy of Sciences, Institute of Vertebrate Paleontology and Paleoanthropology, Chinese Academy of Sciences, Beijing, China; 3 Paleobiology, Canadian Museum of Nature, Ottawa, Ontario, Canada; 4 Henan Academy of Land and Resources Sciences, Zhengzhou, Henan, China; 5 Graduate University of Chinese Academy of Sciences, Beijing, China; 6 School of Earth Sciences, University of Bristol, Bristol, United Kingdom; 7 School of Biological and Chemical Sciences, Queen Mary University of London, London, United Kingdom; 8 Xixia Museum of Dinosaur Fossil Eggs of China, Xixia, Henan, China; Royal Ontario Museum, Canada

## Abstract

**Background:**

Southwestern Henan Province in central China contains many down-faulted basins, including the Xixia Basin where the Upper Cretaceous continental sediments are well exposed. The Majiacun Formation is a major dinosaur-bearing stratigraphic unit that occurs in this basin.

**Methodology/Principal Findings:**

A new basal hadrosauroid dinosaur, *Zhanghenglong yangchengensis* gen. et sp. nov., is named based on newly collected specimens from the middle Santonian Majiacun Formation of Zhoujiagou Village, Xixia Basin. Two transitional features between basal hadrosauroids and hadrosaurids are attached to the diagnosis of the new taxon, namely five maxillary foramina consisting of four small scattered ones anteroposteriorly arranged in a row and a large one adjacent to the articular facet for the jugal, and dentary tooth crowns bearing both median and distally offset primary ridges. *Zhanghenglong* also displays a unique combination of plesiomorphic and derived features of hadrosauroids, and is clearly morphologically transitional between basal hadrosauroids and hadrosaurids. Furthermore, some measurement attributes in osteology are applied to the quantitative analysis of *Zhanghenglong*. For these attributes, the partition of the dataset on most hadrosauroid species resulting from model-based cluster analysis almost matches taxonomic separation between basal hadrosauroids and hadrosaurids. Data of *Zhanghenglong* on selected measurement attributes straddle the two combinations of intervals of partitioned datasets respectively related to basal hadrosauroids and hadrosaurids. This condition is similar to mosaic evolution of morphological characters present in the specimens of the taxon. The phylogenetic analysis of Hadrosauroidea recovers a clade composed of *Zhanghenglong*, *Nanyangosaurus*, and Hadrosauridae with an unresolved polytomy.

**Conclusions/Significance:**

*Zhanghenglong* is probably a relatively derived non-hadrosaurid hadrosauroid, based on the inferences made from the morphological comparisons, quantitative evaluation of measurements, and cladistic analysis. In combination with information on the stratigraphy, phylogeny and biogeography, the material of *Zhanghenglong* provides direct evidence for the hypothesis that hadrosaurids might have originated in Asia.

## Introduction

Hadrosauroidea is a diverse and highly specialized clade of herbivorous dinosaurs whose remains have been found in the late Early and Late Cretaceous (Aptian to late Maastrichtian) of Europe, Asia, the Americas, and Antarctica [1]–[3]. It is amongst the most morphologically derived groups within Ornithischia, consisting of the most recent common ancestor of *Equijubus normani* and *Parasaurolophus walkeri* plus all its descendants [1]. Since the middle of the 20th century, the group has been recognized as a substantial component of the terrestrial vertebrate faunas of the Late Cretaceous [2], [4]–[6]. The known fossil record of hadrosauroids is one of the richest and best preserved nonrenewable resources of the Dinosauria, and includes dozens of articulated skeletons in addition to multi-individual bonebed assemblages, eggs and embryonic material, soft-tissue impressions, and footprints [4], [7]–[10].

Definitions of relevant taxa are described as follows. Hadrosauriformes is the least inclusive clade containing *Iguanodon bernissartensis* and *Parasaurolophus walkeri* [11]. Hadrosauroidea is the least inclusive taxon containing *Equijubus normani* and *Parasaurolophus walkeri*, which follows the phylogenetic topology argued by You et al. [1]. This clade has a series of unambiguous synapomorphies, including the elongate anteroventral corner of the lacrimal, the elevated, subrectangular jugal sutural surface of the maxilla that closely abuts the lacrimal sutural surface of the bone, and the moderately expanded transversely oral margin of the rostrum that is greater than 1.5 times as wide as the maximum constriction of the snout. Hadrosauridae is the monophyletic group consisting of *Saurolophus osborni*, *Parasaurolophus walkeri*, their most recent common ancestor, and all other descendants [11]. Given the close affinities of *Hadrosaurus foulkii* with non-lambeosaurine hadrosaurids (see below), Hadrosauridae could be traditionally divided into two major clades: Hadrosaurinae (the flat-headed or solid-crested hadrosaurids) and Lambeosaurinae (the hollow-crested hadrosaurids) [2].

Basal hadrosauroids, or non-hadrosaurid hadrosauroids, display a series of plesiomorphic features, such as a poorly defined posterior margin of the circumnarial fossa, a relatively low maxillary body with a posteriorly offset dorsal ramus, a nearly vertical or posteriorly inclined coronoid process just concealed entirely by the posterior region of the dental battery in medial view, and one or two active teeth per dentary alveolar position [1], [3], [12]. Over the last century and the beginning of the present one, numerous valuable discoveries of basal hadrosauroids in Asia have filled the gap in our understanding of the early evolution of the clade, particularly regarding significant morphological changes that took place from basal hadrosauriforms to more derived hadrosaurids lasting during the Late Cretaceous [2]. The first Asian basal hadrosauroid species to be erected and described was *Tanius sinensis* from the Jiangjunding Formation of the Wangshi Group, China [13]. Subsequently, Gilmore [14] briefly described another two basal hadrosauroids, *“Mandschurosaurus” mongoliensis* and *Bactrosaurus johnsoni*, from the middle to late Campanian Iren Dabasu Formation of Inner Mongolia, north China, based upon a large number of disarticulated cranial and postcranial bones [15]. Considering *Mandschurosaurus* as a *nomen dubium*, Brett-Surman [16] removed this original genus name, and then extended a new genus *Gilmoreosaurus* to the firstly named hadrosauroid species in Gilmore’s paper [14], with the new combination *G. mongoliensis*. In 1966, *Probactrosaurus gobiensis* was established and preliminarily described by Rozhdestvensky on the basis of three partial skeletons and numerous other bones recovered from the Bayingobi Formation in the Maortu area of Inner Mongolia [17]. After this work, Norman [12] further conducted an overall osteological re-evaluation of the taxon, which drew support from the cladistic analysis. Based on a partial postcranial skeleton recovered from the early Late Cretaceous Xiaguan Formation in the Xiaguan-Gaoqiu Basin of southwest Henan Province, Xu et al. [18] named and thoroughly described *Nanyangosaurus zhugeii* that possibly represents another basal hadrosauroid. However, the phylogenetic position of the taxon is unresolved and controversial all the while. More recently, three primitive non-hadrosaurid hadrosauroids have been found in succession from the late Early Cretaceous (Aptian–Albian) middle grey member of the Xinminbao Group in the Mazongshan area of Gansu Province [19]: *Equijubus normani* [1], *Jintasaurus meniscus* [20], and *Xuwulong yueluni* [21]. The discoveries of these species in East Asia indicate that the ancestral area of Hadrosauroidea is very likely located in Asia [1], [21]. Sues and Averianov [22] revised the systematics and paleobiogeography of Hadrosauroidea, and reported a new basal hadrosauroid taxon *Levnesovia transoxiana* from the Turonian deposits of Central Asia, which has a close affinity with *B. johnsoni*. Furthermore, two additional basal hadrosauroid taxa, *Shuangmiaosaurus gilmorei* [23] and *Nanningosaurus dashiensis* [24], are known from the Upper Cretaceous of northeast and southwest China, respectively. Some basal hadrosauriforms outside of Hadrosauroidea have also been reported in Asia, including *Altirhinus kurzanovi* [25] and *Jinzhousaurus yangi* [26].

Apart from Asia, basal hadrosauroids are explicitly recorded in Europe (*Telmatosaurus transsylvanicus* [27] and *Tethyshadros insularis* [28]) and North America (e.g., *Eolambia caroljonesa* [29] and *Protohadros byrdi* [30]). The fossil record of Europe and North America substantially expands the biodiversity, stratigraphic presence, and paleogeographic distribution of non-hadrosaurid hadrosauroids. Although the known record of basal hadrosauroids has provided a wealth of information on the morphology and diversity of this paraphyletic group, whose phylogenetic interrelationships are becoming better understood, broad consensus on the evolutionary history and systematic positions of derived basal hadrosauroids remains elusive. A major cause of this lack of consensus is the fact that some relatively derived basal hadrosauroids are known only from partial or fragmentary remains, so that a large amount of character information is missing from the fossil record.

**Figure 1:**
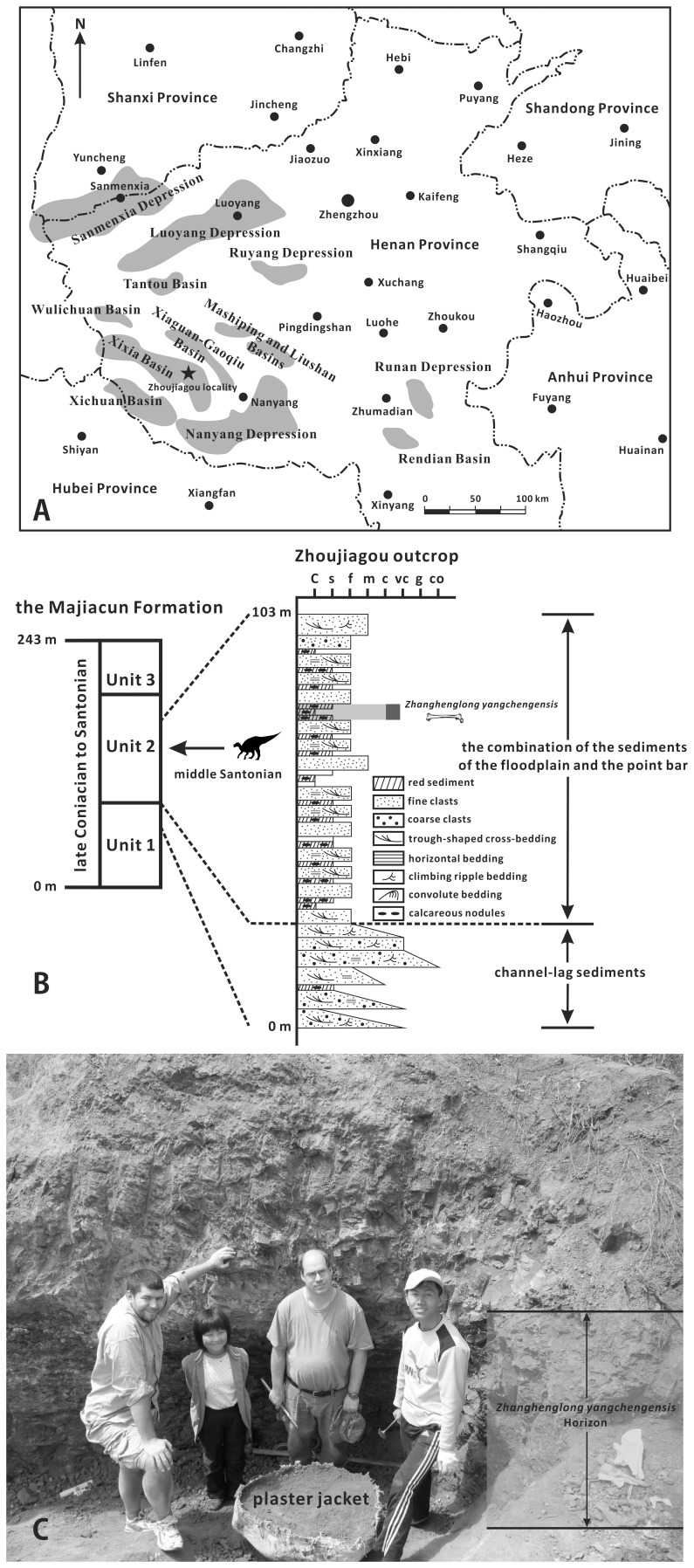
Simplified geographic map and detailed stratigraphic section showing the locality and horizon of *Zhanghenglong yangchengensis*. (A) Geographic map showing the down-faulted basins and depressions (dark grey areas) of southwest Henan Province, China where Upper Cretaceous continental sediments are well developed and the locality of *Z. yangchengensis* (the black five-pointed star) in the Xixia Basin. (B) Stratigraphic section of the Zhoujiagou outcrop that represents most of the middle portion (Unit 2) of the Majiacun Formation and the upper part of the lower portion (Unit 1) of the same formation, with the horizon of *Z. yangchengensis* indicated by an arrow. (C) Excavation of the Zhoujiagou locality in 2011.

The southwestern part of Henan Province in central China contains many down-faulted basins or depressions in which Upper Cretaceous continental sediments are widely distributed, including the Xixia, Xiaguan-Gaoqiu, and Xichuan Basins [31] (Fig. 1A). The Majiacun Formation is a major dinosaur-bearing lithostratigraphic unit that is exposed in the uplifted areas along the edges of the Xixia Basin and the neighboring Xichuan Basin; the formation is bounded above and below by its conformable contacts with the overlying Sigou Formation and the underlying Gaogou Formation, respectively [31], [32].

This paper describes a new basal hadrosauroid genus and species from the Upper Cretaceous Majiacun Formation, based on some disarticulated bones collected from Zhoujiagou Village in the Xixia Basin of southwest Henan Province, China (Fig. 1A). The quarry site is located approximately 0.4 km southeast of the centre of the village. According to palynological and stratigraphic data obtained from the recent regional geological survey of Henan Province, the outcrop in Zhoujiagou Village is generally equivalent to most of the middle portion (Unit 2) of the Majiacun Formation plus the upper part of the lower portion (Unit 1) of the formation [31] (Fig. 1B). Unit 2 could be interpreted as a combination of floodplain and point bar deposits, clearly differing from the coarse-grained channel-lag sediments in Unit 1. The layer from which the hadrosaur material was recovered is embedded within Unit 2 of the Majiacun Formation. It consists of a mixture of light purple and red muddy siltstone or mudstone and calcareous nodules that represents well-developed palaeosols, as is characteristic of the fine-grained member of typical meandering stream deposits (Fig. 1B, C). Although the Majiacun Formation extends in age from the late Coniacian through to the end of the Santonian, the dinosaur-bearing bed very likely dates to the middle Santonian [31], [33]. In addition to the hadrosauroid material, the formation has yielded dinosaur eggs, a spinosaurid tooth, the incomplete articulated postcranial skeleton of an alvarezsaurid, a partially preserved sacrum belonging to an ankylosaurid, sauropod cervical vertebrae, turtle shell elements, and crocodilian dorsal vertebrae [31], [33], [34].

The osteological description of the new basal hadrosauroid taxon presented in this paper may be helpful in understanding the morphological transition between basal hadrosauroids and more derived hadrosaurids. Furthermore, the phylogenetic analysis carried out as part of the study could provide new insights into the evolutionary relationships among derived basal hadrosauroids and hadrosaurids, and helps to test the potential dispersal patterns that have been previously suggested for basal hadrosauroids. Attention is also paid to the statistical partitions of osteological measurements; the dataset of each measurement attribute is the collection of the corresponding values for most species within Hadrosauroidea and several close relatives of this group. The statistical analysis is enormously enlightened by Prieto-Márquez’s work [3].

Institutional Abbreviations: AEHM, Amur Natural History Museum, Blagoveschensk, Russia; AMNH, American Museum of Natural History, New York, USA; CCMGE, Chernyshev’s Central Museum of Geological Exploration, Saint Petersburg, Russia; CEUM, College of Eastern Utah Prehistoric Museum, Price, USA; CMN, Canadian Museum of Nature, Ottawa, Canada; FGGUB, Facultatea de Geologie si Geofizica, Universitatea Bucuresti, Bucharest, Romania; FMNH, The Field Museum, Chicago, USA; GMV, Geological Museum of China, Beijing, China; GSGM, Gansu Geological Museum, Lanzhou, China; IVPP, Institute of Vertebrate Paleontology and Paleoanthropology, Beijing, China; LPM, Liaoning Paleontological Museum, Beipiao, China; MOR, Museum of the Rockies, Bozeman, USA; MPZ, Museo Paleontológico de la Universidad de Zaragoza, Zaragoza, Spain; NMMNH, New Mexico Museum of Natural History and Science, Albuquerque, USA; PIN, Paleontological Institute of the Russian Academy of Sciences, Moscow, Russia; PMU, Museum of Evolution, Uppsala University, Uppsala, Sweden; RAM, Raymond M. Alf Museum, Claremont, USA; ROM, Royal Ontario Museum, Toronto, Canada; SBDE, Sino-Belgian Dinosaur Expedition; SC, Italian State collections; SMU, Shuler Museum of Paleontology, Southern Methodist University, Dallas, USA; TMP, Royal Tyrrell Museum of Paleontology, Drumheller, Canada; USNM, United States National Museum, Smithsonian Institution, Washington, DC, USA; XMDFEC, Xixia Museum of Dinosaur Fossil Eggs of China, Xixia, China.

## Methods

The necessary permits for the described field studies were obtained from the Department of Land and Resources of Henan Province (Zhengzhou, China), XMDFEC (Xixia, China), and IVPP (Beijing, China). Permission was also granted to the authors for accessing the collection of fossils at the XMDFEC (Xixia, China), in order to examine the hadrosauroid materal collected from the fieldwork and housed at the museum. In addition, the individual in this manuscript has given written informed consent (as outlined in PLOS consent form) to publish these case details.

### Nomenclatural Acts

The electronic edition of this article conforms to the requirements of the amended International Code of Zoological Nomenclature, and hence the new names contained herein are available under that Code from the electronic edition of this article. This published work and the nomenclatural acts it contains have been registered in ZooBank, the online registration system for the ICZN. The ZooBank LSIDs (Life Science Identifiers) can be resolved and the associated information viewed through any standard web browser by appending the LSID to the prefix “http://zoobank.org/”. The LSID for this publication is: urn:lsid:zoobank.org:pub:AC2A0339-382D-483D-874E-DB3188DEBE90. The electronic edition of this work was published in a journal with an ISSN, and has been archived and is available from the following digital repositories: PubMed Central, LOCKSS.

### Material

All available material assigned to the new basal hadrosauroid taxon described in this paper was collected from three plaster field jackets that are removed from a single quarry, including a very small one (Fig. 1C). The largest one of the three jackets measures about 1.8 m in length. The fossil assemblage in the three jackets is composed of disarticulated elements, some of hadrosauroid origin and some belonging to non-dinosaurian reptiles. The hadrosauroid material in the jackets has undergone detailed morphological examination. It includes a variety of cranial and postcranial bones, all of which are approximately proportionate in size. All of the cranial bones come from the same field jacket. These bones are referable to at least two individuals representing two taxa, and the osteological discrepancies between the two taxa are very striking: some cranial elements bear a close resemblance to their counterparts in the basal hadrosauroid *Gilmoreosaurus mongoliensis* from Inner Mongolia [14], whereas the others show derived features that distinguish them from the equivalent bones in *Gilmoreosaurus*. The new basal hadrosauroid genus and species described herein is the more derived taxon recovered from the quarry. We retrieved five sets of paired cranial elements, namely the maxillae, jugals, quadrates, dentaries, and surangulars, all in close proximity to one another. The general morphology of the paired quadrates and surangulars is very similar to that in *G. mongoliensis*. For the paired maxillae, jugals, and dentaries, the left member of the pair appears to be *Gilmoreosaurus*-like, whereas the right member is morphologically different and more derived. For example, the ectopterygoid ridge of the right maxilla is robust and straight along its entire length; the medial face of the anterior process of the right jugal is bounded posteriorly by a crescent-shaped articular facet for the palatine; in dorsal view of the right dentary, the long axis of the tooth battery is nearly parallel with the lateral side of the dentary ramus. These characters are very common among hadrosaurids but rarely reported in basal hadrosauroids. In addition, the occlusal surfaces of the right maxillary and dentary tooth rows are markedly convex dorsally, and are complementary to each other; the maxillary facet of the right jugal also matches the sutural surface for the jugal on the lateral side of the right maxilla. These different lines of evidence suggest that the right maxilla, jugal, and dentary were associated with a single individual in life. Thus, the cranial elements that show derived features relative to *G. mongoliensis* (the right maxilla, jugal, and dentary) comprise the holotype of the new taxon described here. It is difficult to assign a number of postcranial bones in the jackets to either taxon because these bones are not markedly different from their equivalents observed in *G. mongoliensis* and other hadrosauroids. Nevertheless, there is one exception to this condition. A right scapula, a right ulna, five anterior-middle dorsal vertebrae (D1 and D4–D7) preserved in series, and some dorsal ribs occur together in a small region. They are disarticulated, but appear to be in association. Similar to the condition seen in the right maxilla, jugal and dentary, the right scapula has a derived feature that is typical of hadrosaurids, namely the arcuate dorsal edge of the scapular blade. In addition, the neural spines of the anterior dorsals, which are nearly complete in D1 and D5, are more elongate than those in *G. mongoliensis*. Therefore, these postcranial elements may come from one individual of the new basal hadrosauroid described here, and are tentatively designated as the paratype of the taxon. Most of the bones assigned to the new basal hadrosauroid taxon are generally well-preserved, but the right maxilla in particular appears to have undergone substantial distortion owing to anomalous preservation.

### Osteological Comparisons and Cladistic Analysis

All known fossils of the new taxon are stored at the XMDFEC. The osteological description of the fossil material is conducted on the basis of detailed qualitative observations supplemented by relevant linear measurements. Comparative information on osteological features in other iguanodontian dinosaurs was obtained from direct observations and the literature. The phylogenetic position of the new taxon was inferred via the maximum parsimony analysis using the tree bisection reconnection (TBR) algorithm in the program TNT [35]. To assess the stability of the major clades within the Hadrosauroidea that were found to include the new taxon described in this paper, bootstrap and Bremer decay values were calculated using the option “Resampling” with 1000 replicates and the script “BREMER.RUN”, respectively.

### Model-based Clustering

Model-based cluster analysis (MCA) is a comprehensive mathematical method for partitioning a dataset using the expectation-maximization (EM) algorithm, with the initial classification obtained from agglomerative hierarchical clustering and direct comparisons of potential partition models based on values of the Bayesian Information Criterion (BIC) [36], [37]. Prieto-Márquez [3] used MCA, as implemented in the package Mclust of the statistical program R, to divide each of three morphology-based datasets into several discrete intervals with an optimal number of clusters. After this clustering, the intervals of the one-dimensional dataset were objectively transformed into independent but associated character states used for phylogenetic analysis [3]. We follow Prieto-Márquez’s application of MCA to evaluate the taxonomic status of the new hadrosauroid. Measurements derived from phylogenetic characters include linear measurement ratios and angles. The one-dimensional database of each measurement attribute is constituted by the measured values for most available hadrosauroid species and some iguanodontians outside of Hadrosauroidea. However, the data of the new taxon are not allowed to participate in MCA. Prior to the application of MCA, the sample mean was used to represent the multiple values obtained from more than one specimens of a certain species. For some attributes referring to almost all hadrosauroid taxa except the newly erected one, the partition of the dataset implemented using MCA is generally aligned with the taxonomic separation between basal hadrosauroids and more derived hadrosaurids. These attributes are very significant for the validation of the new taxon probably representing a basal hadrosauroid dinosaur. They could therefore be selected for the quantitative analysis of the relevant data of this taxon. When the data of the new taxon lie in the intervals of selected measurement attributes, the total distribution of the data could test the potential taxonomic status of the taxon deduced from morphological comparisons among all hadrosauroid taxa to some extent.

## Results

### Systematic Paleontology

Dinosauria Owen, 1842 [38]

Ornithischia Seeley, 1887 [39]

Ornithopoda Marsh, 1881 [40]

Iguanodontia Dollo, 1888 [41] *sensu* Sereno, 1998 [11]

Ankylopollexia Sereno, 1986 [42] *sensu* Sereno, 1998 [11]

Styracosterna Sereno, 1986 [42] *sensu* Sereno, 1998 [11]

Hadrosauriformes Sereno, 1997 [43] *sensu* Sereno, 1998 [11]

Hadrosauroidea Cope, 1870 [44] *sensu* You et al., 2003 [1]

*Zhanghenglong* gen. nov

urn:lsid:zoobank.org:act:0ADAC26E-2FFB-4276-8E93-E0FBF034D97A

#### Type species

*Zhanghenglong yangchengensis* sp. nov.

#### Etymology

“Zhangheng” is derived from the full name of Mr. Zhang Heng, a famous Chinese astronomer, mathematician, inventor, poet, and statesman who lived during the Eastern Han Dynasty (AD 25–220) of China. The figure was born in the outskirts of Nanyang in southwestern Henan Province, quite close to the Xixia Basin. The word “long” is the direct transliteration of the Mandarin Chinese word that means dragon.

#### Diagnosis

As for the type and only species.

*Zhanghenglong yangchengensis* sp. nov.

urn:lsid:zoobank.org:act:60BA715B-EF20-449C-8E28-3450C4E80D9A

#### Holotype

XMDFEC V0013, an incomplete, disarticulated cranium, including the nearly complete right maxilla, as well as the partial right jugal and dentary.

#### Paratype

XMDFEC V0014, a partial, disarticulated postcranial skeleton, including five anterior-middle dorsal vertebrae (some of which lack the dorsal portion of the neural spine), fragments of the dorsal ribs, and the nearly complete right scapula and ulna.

#### Etymology

The specific name is derived from a large administrative region called Yangcheng that was established in the Spring and Autumn period (BC 770–403) of the Eastern Zhou Dynasty of China. This ancient administrative region included what is now southwestern Henan Province.

#### Locality and horizon

Zhoujiagou Village, about 5 km northeast of Sanlimiao Village where the Xixia Dinosaur Relics Park is located, Xixia Basin, Xixia County, Henan Province (Fig. 1A). The quarry probably occurs in the middle of the middle member (Unit 2) of the Majiacun Formation (Fig. 1B), and is thought to be the middle Santonian in age [31].

#### Diagnosis

Medium-sized hadrosauroid ornithopod characterized by the following autapomorphies: strongly deflected posteroventrally posterior third of the maxilla relative to the anterior two thirds of the same element, and crowns of dentary teeth with both median and distally offset primary ridges. Also diagnosed by a unique combination of the following features: five maxillary foramina consisting of four scattered small ones anteroposteriorly arranged in a row and a large one close to the ventral extremity of the jugal articular surface of the maxilla; markedly convex dorsally maxillary and dentary occlusal surfaces; elevated maxillary body that is approximately 150% longer than tall; laterally exposed, large anterior foramen limited to the anterior half of the anterodorsal surface of the maxilla and located just lateral to the premaxillary articular surface of the bone; relatively long, well-developed ectopterygoid ridge; dorsoventrally low anterior process of the jugal with a lunate articular facet for the palatine along its posterior border; dorsoventrally deep embayment along the ventral margin of the jugal; dentary showing 26 alveolar positions; long axis of the dentary occlusal surface parallel with the lateral side of the dentary ramus; maxillary tooth crowns with sigmoid and nearly straight primary ridges; one or two functional teeth per alveolus for most of the dentary occlusal surface; no more than four teeth per dentary alveolus; scapular neck strongly constricted dorsoventrally; and dorsal margin of the scapula arcuate.

### Osteological Description

**Figure 2:**
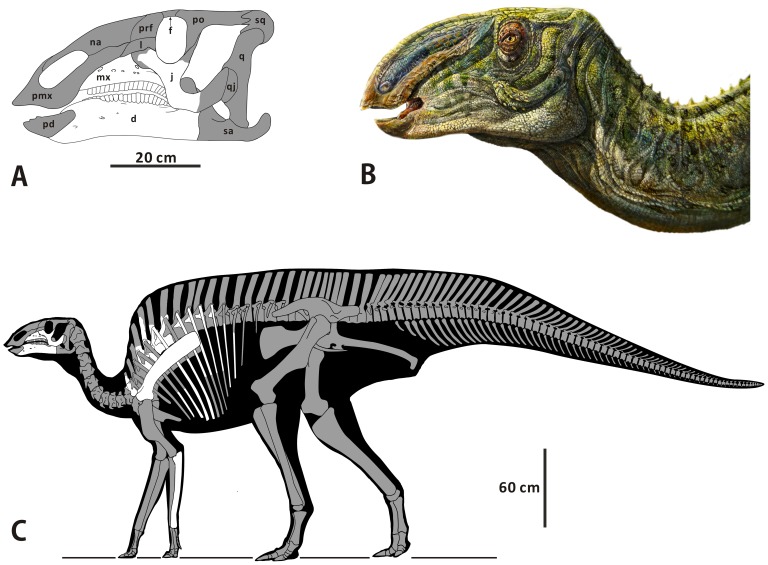
Reconstruction and restoration of the skeleton of *Zhanghenglong yangchengensis*. (A) Skull reconstruction of *Z. yangchengensis* in left lateral view. (B) Restoration of the head and the anterior part of the neck of *Z. yangchengensis* in left lateral view. (C) Skeleton reconstruction of *Z. yangchengensis* in left lateral view. Bones in white are preserved in the specimens of *Z. yangchengensis* (XMDFEC V0013 and V0014). Bones in grey are unknown. Abbreviations: d, dentary; f, frontal; j, jugal; l, lacrimal; mx, maxilla; na, nasal; pd, predentary; pmx, premaxilla; po, postorbital; prf, prefrontal; q, quadrate; qj, quadratojugal; sa, surangular; sq, squamosal.

The material of *Zhanghenglong yangchengensis* consists of the holotype (XMDFEC V0013) and the paratype (XMDFEC V0014). Partial measurements of these specimens with relevant data comparisons are shown in the online documents (see Supporting Information S1). Reconstructions of the skull and skeleton of the new genus and species are proposed in Fig. 2.

#### Maxilla (XMDFEC V0013)

**Figure 3:**
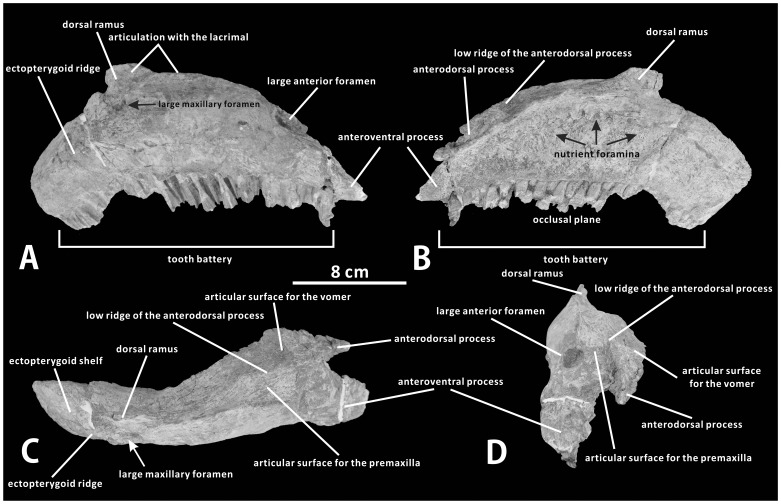
Maxilla of *Zhanghenglong yangchengensis*. Right maxilla (XMDFEC V0013, holotype) in lateral (A), medial (B), dorsal (C), and anterior (D) views.

The maxilla is asymmetrically subtriangular in lateral view, with the dorsal ramus moderately offset towards the posterior end of the bone (Fig. 3A, B). Although the tip of the dorsal ramus is broken away, it is clear that the maxilla is dorsoventrally high, the length of the bone measuring about 2.5 times the estimated height. Anterior to the large anterior foramen, the anteroventral process is slightly pendent. The edentulous anterior end of the anteroventral process is significantly shorter than that in some other non-hadrosaurid hadrosauroids, such as *Shuangmiaosaurus gilmorei* (LPM 0165). The lateral part of the anteroventrally inclined dorsal surface of the anteroventral process extends anteriorly from the large anterior foramen, and would have been exposed in the articulated skull, whereas the medial part forms most of the anterior portion of the articular surface for the premaxilla (Fig. 3C, D). As in other non-hadrosaurid hadrosauriforms such as *Jinzhousaurus yangi* (IVPP V12691), the large anterior foramen is elliptical and laterally exposed; it is located within the anterior half of the anterodorsal surface of the maxilla and just lateral to the articular surface for the premaxilla [3], [26]. In contrast, the foramen is limited to the posterior half of the anterodorsal surface of the maxilla in hadrosaurids. For example, in lambeosaurines such as *Hypacrosaurus altispinus* (e.g., ROM 702) and *Parasaurolophus tubicen* (e.g., NMMNH P-25100), the large anterior foramen is displaced posterodorsally relative to its position in basal hadrosauroids, lying just anterior to the articular surface for the jugal, and is obscured by the premaxilla in lateral view. In *Zhanghenglong*, the dorsolateral edge of the anteroventral process is moderately inclined in lateral view, forming a 32° angle with the anterior third of the ventral margin of the maxilla. Dorsally, the articular surface for the posteroventral process of the premaxilla forms a subtriangular trough between the prominent lateral edge of the dorsal surface of the maxilla and an anteroposteriorly oriented ridge separating the premaxillary and vomerine facets (Fig. 3C). This trough gradually becomes dorsoventrally shallower and mediolaterally narrower as it extends posteriorly towards the articular surface for the lacrimal.

Medial to the anteroventral process, the anterodorsal process of the maxilla is medially offset from the anterior third of the main part of the bone, showing the shape of a small, triangular, anteriorly directed prong. In dorsal view, it forms a 22° angle with the maxillary body, in contrast to the relatively low angle of 10° seen in *Gilmoreosaurus mongoliensis* (AMNH FARB 30653). The anterodorsal process projects anteroventrally, and is slightly shorter than the more laterally positioned anteroventral process, as in *Bactrosaurus johnsoni* (AMNH 6553) and *Protohadros byrdi* (SMU 74582). The condition contrasts with an elongate, raised anterodorsal process of the maxilla observed in hadrosaurines [3], [45]. The ridge separating the premaxillary and vomerine facets extends anteriorly onto the anterodorsal process, so that the lateral part of the process contacts the premaxilla whereas the medial part meets the vomer (Fig. 3C, D). It represents the prominent, narrow, somewhat dorsally projecting lateral edge of the articular surface for the vomer. The dorsal margin of the ridge is weakly convex and slightly inclined anteroventrally. It is angled more gently than the dorsolateral margin of the anteroventral process. The part of the anterodorsal process contacting the vomer forms a thin, medially projecting shelf, below which perches the anterior portion of the medial wall of the maxilla that covers the anterior region of the dental battery.

The articular surface for the jugal is relatively low dorsoventrally along the lateral side of the dorsal ramus, a short distance above the anterior end of the ectopterygoid ridge. By contrast, the corresponding articular surface in hadrosaurids other than *Pararhabdodon isonensis* and *Tsintaosaurus spinorhinus* is dorsoventrally deep and in contact, at its ventral extremity, with the anterior end of the ectopterygoid ridge [46], [47]. Although the dorsal ramus is not intact in the holotype of *Zhanghenglong*, this structure is sufficiently well preserved to confirm that the articular facet for the jugal has a subrectangular outline in lateral view as in other basal hadrosauroids. The jugal articular surface of the maxilla is composed of the dorsal portion on the lateral side of the dorsal ramus and the ventral portion located anterodorsal to the ectopterygoid shelf. The dorsal portion of this surface faces laterally, whereas the ventral portion faces dorsolaterally. The surface tapers anteriorly to a peak at the level of its dorsoventral midpoint, suggesting that the anterior process of the jugal would have tapered in a similar way, and is separated by an anteroventrally directed ridge from the articular surface for the lacrimal. The posteroventral corner of the articular surface for the jugal forms a posterolaterally directed prominence, which is preserved in contact with a small fragment that may represent part of the jugal. Above the prominence, the posterior margin of the articular surface for the jugal is built up into a slight, lateroventrally oriented ridge. The maxilla meets the lacrimal laterally and dorsally, and forms the articular surface for the latter that occupies the anterodorsal region of the lateral side of the dorsal ramus, as well as a stretch of the mediolaterally narrow dorsal side between the posterior end of the articular surface for the premaxilla and the anterodorsal apex of the dorsal ramus. It is more likely that the jugal did not strongly interfere with the laterally visible maxilla-lacrimal joint in the fully articulated skull. The articular surface for the lacrimal abuts the more posteroventrally positioned articular surface for the jugal along the lateral side of the dorsal ramus, as in many basal hadrosauroids. By contrast, in some basal hadrosauriforms such as *Altirhinus kurzanovi* (e.g., PIN 3386/7), the finger-shaped articular facet for the jugal is strongly offset posteroventrally from the dorsal ramus, where the articular facet for the lacrimal occurs.

A large, oval foramen is situated anterodorsal to the ectopterygoid ridge and just below the anteroventral margin of the articular surface for the jugal. The condition is apparently more similar to hadrosaurids, in which the posteriormost one or two maxillary foramina are enlarged and adjacent to the ventral extremity of the articular facet for the jugal when a series of maxillary foramina with the total number less than seven are arranged in an anterodorsally oriented row and dorsally positioned. In *Zhanghenglong*, four small additional foramina are also laterally visible, forming an irregular horizontal row near the ventral margin of the lateral side of the maxilla; the first two of these small foramina are positioned far anterior to the others. Typical basal hadrosauroids resemble *Zhanghenglong* in having a ventrally positioned, nearly horizontally aligned row of small, irregularly spaced foramina. Therefore, *Zhanghenglong* shows similarities to both hadrosaurids and typical basal hadrosauroids in the configuration of the foramina on the lateral surface of the maxilla. This condition in *Zhanghenglong* could be interpreted as an intermediate stage. The total count of five foramina on the lateral surface of the maxilla of *Zhanghenglong* is unusually low for other basal hadrosauroids with the exception of *Telmatosaurus transsylvanicus* (FGGUB R1010, three or four maxillary foramina) [27] and *Tethyshadros insularis* (SC 57021, three maxillary foramina) [28].

As in other hadrosauroids, the posterior third of the maxilla bears the palatine and pterygoid processes medially, the ectopterygoid ridge laterally, and the ectopterygoid shelf in between. Interestingly, the part of the maxilla is strongly deflected posteroventrally, forming an angle of 43° with the anterior two thirds of the bone. This feature is comparable to, although not as same as, the strongly curved ventrally posterior half of the maxilla seen in *Shuangmiaosaurus gilmorei* [23]. However, the possibility that this downturning of the maxilla in *Zhanghenglong* is the result of distortion cannot be excluded. Posteroventral to the articular surface for the jugal, the ectopterygoid shelf is transversely broad and flat, and extends laterally to form a prominent lip-shaped ectopterygoid ridge. The ectopterygoid ridge is very robust and straight along its entire length; it is approximately 36% as long as the maxillary body. Such a ridge bears a distinct resemblance to the equivalent structure in hadrosaurids such as *Edmontosaurus regalis* (e.g., CMN 2289) and *Tsintaosaurus spinorhinus* (e.g., IVPP V725). In contrast, the slightly shorter ectopterygoid ridge is weakly developed anteriorly but becomes dorsoventrally thick posteriorly in basal hadrosauroids [3], such as *Levnesovia transoxiana* (CCMGE 579/12457). In lateral view, the ectopterygoid shelf and ridge are moderately inclined posteroventrally even relative to the posterior portion of the maxilla, being deflected downwards at an angle of 16° rather than nearly horizontal as in most hadrosaurids [3], [48]. The palatine and pterygoid processes are too eroded to provide detailed morphological information. They protrude dorsally from the medial margin of the ectopterygoid shelf, and respectively form an anterior contact with the palatine and a posterior contact with the pterygoid.

The medial surface of the maxilla is perfectly flat, and is pierced by a dorsally convex row of round nutrient foramina that are typical of hadrosauroids (Fig. 3B). The nutrient foramina are limited to the dorsal half of the maxilla. The number of nutrient foramina is consistent with that of maxillary tooth positions. The dental battery of the maxilla includes a minimum of 27 tooth positions. As preserved, the occlusal surface is dorsally convex because of the downward curvature of the posterior third of the maxilla, and is also inclined to face ventromedially.

#### Jugal (XMDFEC V0013)

**Figure 4:**
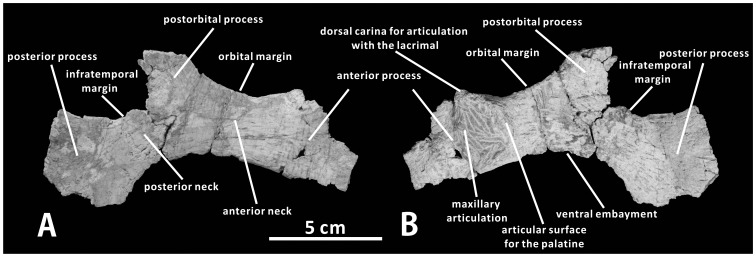
Jugal of *Zhanghenglong yangchengensis*. Right jugal (XMDFEC V0013, holotype) in lateral (A) and medial (B) views.

The jugal is a triradiate bone that contributes to the ventral margins of the orbit and the infratemporal fenestra (Fig. 4A, B). As seen in other iguanodontians, it consists of the anterior process, ascending postorbital process, and posterior process, as well as two dorsoventrally constricted necks [2], [49]. This element is mediolaterally compressed and laterally convex from its anterior contact with the maxilla and lacrimal to its posterior union with the quadratojugal and quadrate. Unfortunately, only the right jugal is present in the holotype, and this element is so incomplete and poorly preserved that much osteological information is missing.

As in basal hadrosauroids like *Equijubus normani* (IVPP V12534) and *Xuwulong yueluni* (GSGM-F00001), the anterior process is slightly expanded dorsoventrally relative to the anterior neck. It is too damaged to be confidently reconstructed, but probably tapered anteriorly. The posterodorsal margin of the anterior process is sufficiently intact to indicate that the process does not strongly expand dorsally. By contrast, in most hadrosaurids such as *Prosaurolophus maximus* (e.g., TMP 84.1.1) and *Corythosaurus casuarius* (e.g., ROM 1933), the anterior process of the jugal is comparatively high relative to the anterior neck of the bone due to a pronounced, strongly recurved, subtriangular dorsal expansion of the process. The lateral side of the anterior process is relatively flat, whereas the medial side bears a markedly concave, medioventrally directed articular facet for the maxilla. This concave facet is ornamented with numerous faint striations that radiate anterodorsally and anteriorly from its posterior margin. The medial face of the anterior process is bounded posteriorly by a crescent-shaped articular facet for the palatine (Fig. 4B). A similar feature is likely present in all hadrosaurids, in which a dorsoventrally elongate, arched articular surface for the palatine occurs along the posterodorsal edge of the medial side of the jugal anterior process [49], [50]. However, the condition observed in *Zhanghenglong* strongly contrasts with that in most non-hadrosaurid iguanodontians, in which a dorsoventrally narrow, strip-like facet for articulation with the palatine is situated on the dorsal border of the medial face of the anterior process [51], [52]. Anterodorsal to the palatine articular surface of the jugal, the dorsal edge of the anterior process forms a narrow carina for contact with the lacrimal. However, only the posteriormost part of the carina remains intact. This configuration is slightly protuberant dorsally so as to meet the posteroventral sulcus of the lacrimal. The articular facet for the maxilla is proportionally higher, with a relatively wide angle of 65° between its posterodorsal and posteroventral borders, than the anteroposteriorly elongate but dorsoventrally narrow equivalent facet seen in *Bactrosaurus johnsoni* (e.g., SBDE 95E5/7) and *Tanius sinensis* (PMU R240). In dorsal view, the anterior process abruptly becomes mediolaterally narrow posteriorly from the posterior margin of the articular surface for the maxilla to contact the anterior neck.

The anterior neck represents a modest dorsoventral constriction of the jugal, the shallowest region of the neck being about 73% as dorsoventrally deep as the anterior process. The dorsal margin of the anterior neck is slightly concave, forming a bridge from the dorsal carina of the anterior process to the base of the postorbital process. The posterior neck is moderately downturned posteroventrally, forming an angle of 143° with the anterior neck, and the posterior neck is also slightly dorsoventrally shallower than the anterior one. The postorbital process projects dorsally, and is gently tilted posteriorly. The dorsal part of this process, which would have contacted the ventral ramus of the postorbital dorsally and laterally, is not preserved. As in other iguanodontians, the postorbital process is very robust at the base, and gradually tapers dorsally. Much of the posterior process of the jugal, including the quadratojugal flange, is broken away. If intact, the posterior process would have projected posterodorsally and formed the ventral part of the posterior margin of the infratemporal fenestra. The shape of the preserved part of the posterior process suggests that this posterodorsal projection would have been more strongly angled posteriorly than the postorbital process. A small part of the posteroventral margin of the posterior process is preserved. It is very likely that this margin is only very shallowly concave, as in other basal hadrosauroids such as *Bactrosaurus johnsoni* (e.g., AMNH 6396) and *Protohadros byrdi* (SMU 74582). The condition differs significantly from the deep concavity along the posteroventral margin of the jugal posterior process observed in *Gryposaurus monumentensis* (e.g., RAM 6797) and *Kritosaurus navajovius* (e.g., AMNH 5799). The ventral half of the infratemporal fenestra, bounded by the postorbital process, posterior process, and posterior neck of the jugal, clearly has a deep, somewhat U-shaped outline in lateral view. The anteroposterior width of the infratemporal fenestra is slightly less than that of the orbit, which is judged from the lateral profile of the bone. Unlike other basal hadrosauroids such as *Tethyshadros insularis* (SC 57021) and *Eolambia caroljonesa* (e.g., CEUM 52204), the ventral edge of the jugal bears a strong embayment whose depth is equal to about 60% of the minimum height of the posterior neck (Fig. 4A, B).

#### Dentary (XMDFEC V0013)

**Figure 5:**
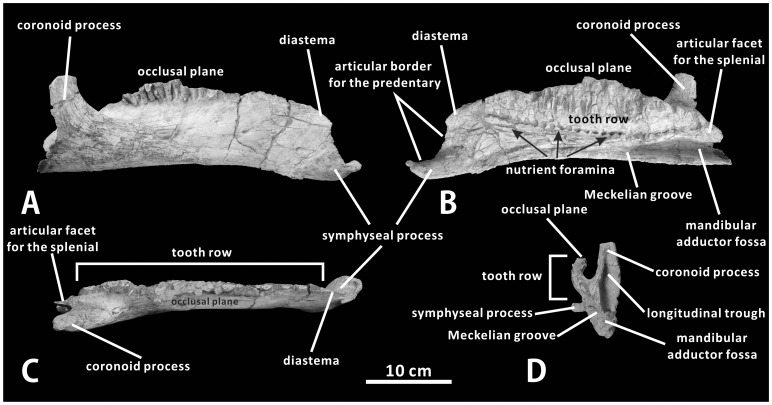
Dentary of *Zhanghenglong yangchengensis*. Right dentary (XMDFEC V0013, holotype) in lateral (A), medial (B), dorsal (C), and posterior (D) views.

The massive right dentary is well preserved and undistorted in XMDFEC V0013. This bone consists of an elongate dentary ramus, a slightly eroded tooth battery, and a nearly vertical coronoid process lacking the dorsal apex (Fig. 5A, B). There are 26 vertical tooth families visible medially along the long axis of the tooth battery. A series of nutrient foramina, located at half height of the medial side of the dentary ramus, are arranged in a slightly convex ventrally row. Each alveolar position strictly corresponds to one of these accessory nutrient foramina at the base of the tooth battery (Fig. 5B). The occlusal surface of the dentary is difficult to thoroughly reconstruct because the teeth are not complete anteriorly. However, it is clearly directed dorsolaterally, and may have had a dorsally convex curvature matching the ventrally concave curvature of the occlusal surface of the maxilla. In contrast, the nearly straight, dorsolaterally-facing occlusal surface of the dentary tooth row is very common among most other iguanodontians such as *Jinzhousaurus yangi* (IVPP V12691) and *Parasaurolophus walkeri* (ROM 768).

Anterior to the first tooth lies a particularly short edentulous region that is the diastema, whose length (42 mm) is only about 17% of the entire length of the tooth battery (247 mm). The diastema gently deflects anteroventrally at a low angle relative to the horizontal posterior part of the dentary ramus. The anterior end of the bone curves lingually and also twists progressively so that the outer surface of the anteriormost part of the dentary is ventrally directed, forming a symphyseal process that would have contacted the predentary anterodorsally and the contralateral dentary along the sagittal plane. The contact between the symphyseal processes of the two dentaries would have represented the posterior part of the mandibular symphysis, and would have appeared ventrally convex in anterior view if the paired dentaries were placed in articulation with the predentary removed. The articular surface for the predentary, located just anteroventral to the diastema, occurs along the anterior margin of the symphyseal process. As in other basal hadrosauroids, the posterodorsal part of the articular border for the predentary forms a rather steep slope in lateral or medial view, as it sharply descends towards the slightly dorsomedially concave platform of the symphyseal process [3]. The angle between this steep slope and the ventral margin of the posterior portion of the dentary ramus is about 70°. The symphyseal process is clearly wider mediolaterally than the main body of the dentary ramus, with the ratio of 1.52 between the mediolateral breadth of the former and that of the latter. In dorsal view, the medial margin of the symphyseal process slightly diverges from the lateral side of the dentary at a relatively low angle of approximately 13° (Fig. 5C). The anteromedial corner of the symphyseal process bears an oval, anteroventrally-facing fossa for articulation with the ventral median process of the predentary [2]. Specifically, the anterior region of the dentary ramus is not strongly deflected anteroventrally, resembling the plesiomorphic condition present in non-hadrosaurid hadrosauriforms such as *Probactrosaurus gobiensis* (PIN 2232/42-1) [12], *Jinzhousaurus yangi* (IVPP V12691) [26], and *Gilmoreosaurus mongoliensis* (AMNH FARB 30654) [53].

The main body of the dentary ramus is pierced laterally by a number of irregularly distributed small foramina of variable size (Fig. 5A). Its lateral surface is gently convex dorsoventrally, and bears numerous anteroposteriorly directed striations. Medially, the Meckelian groove is situated at the posteroventral corner of the dentary ramus, wedging anteriorly along the ventromedial border of the posterior part of the bone. The groove tapers anteriorly and terminates near the anteroposterior midpoint of the ventromedial margin of the dentary ramus (Fig. 5B). The posterior region of the Meckelian groove represents the relatively deep anterior portion of the mandibular adductor fossa. Dorsal to the posterior region of the Meckelian groove and posterior to the tooth battery, the medial surface of the dentary bears a subtriangular articular facet for the splenial. In dorsal view, the long axis of the tooth battery is nearly parallel with the lateral side of the dentary ramus, a feature common among hadrosaurids [3], [53]. However, the long axis of the tooth battery and the lateral side of the dentary ramus diverge from each other towards the coronoid process in most basal hadrosauroids, including *Eolambia caroljonesa* [29], [54] and *Shuangmiaosaurus gilmorei* [23]. The coronoid process is not intact owing to the lack of the dorsal apex, whereas it exhibits typical morphological features of basal hadrosauroids: the coronoid process is nearly perpendicular to the dorsal margin of the dentary ramus; a dorsoventrally elongate, weakly developed ridge occurs on the medial side of the coronoid process; the posteriormost end of the dentary tooth row does not exceed the posterior margin of the coronoid process in medial view. The coronoid process is also well offset laterally from the tooth row, as in other hadrosauroids [2], [3], [12]. There is a transversely broad and dorsoventrally shallow concavity between the tooth row and the base of the coronoid process. In posterior view, the posterior surface of the coronoid process bears a deep, longitudinally elongate trough that extends ventrally to the base of the mandibular adductor fossa (Fig. 5D). The trough probably represented an area of muscle attachment in the living animal.

#### Maxillary teeth (XMDFEC V0013)

**Figure 6:**
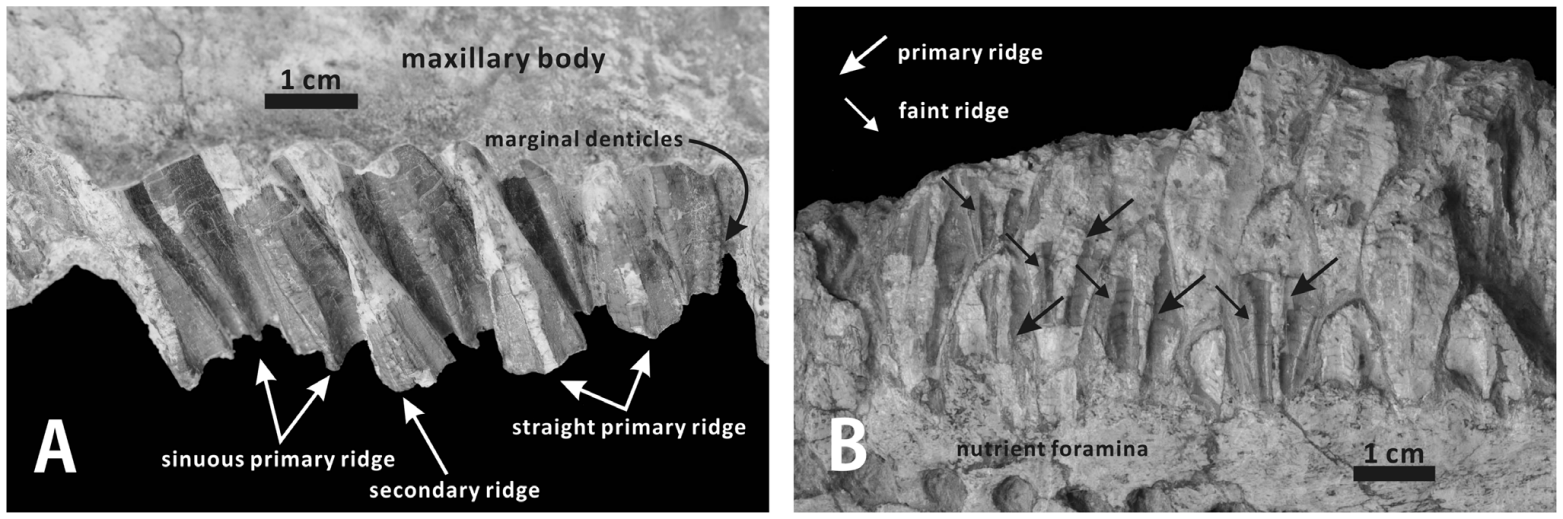
Maxillary and dentary teeth of *Zhanghenglong yangchengensis*. (A) Teeth of the right maxilla (XMDFEC V0013, holotype) in labial view. (B) Teeth of the right dentary (XMDFEC V0013, holotype) in lingual view.

The right maxilla of the holotype retains 27 tooth families, representing the full complement of tooth positions. Some of the tooth families are partially preserved. In lateral view, the enameled labial surface of the crown of each functional tooth is apicobasally elongate, symmetrical and subtriangular, with mesial and distal margins that converge dorsally towards the narrowest base of the crown (Fig. 6A). Unworn replacement teeth are estimated to have measured approximately 35 mm in apicobasal height, although no complete examples are visible along the ventral border of the maxilla. In the middle part of the tooth row, the maximum mesiodistal width of each tooth is about 10 mm. The following features of maxillary teeth observed in XMDFEC V0013 are typical of most hadrosauroids: the tooth crown is labially ornamented with one primary ridge and one weaker secondary ridge; the robust primary ridge is mesiodistally centered and buccally prominent, extending along the entire apicobasal height of the crown; adjacent to the primary ridge, a shortened secondary ridge runs longitudinally throughout the distal half of the labial side of the crown. By contrast, the primary ridges of the maxillary teeth are slightly distally offset in *Equijubus normani* [1], *Xuwulong yueluni* [21], and *Jinzhousaurus yangi* [26]. Interestingly, the median primary ridges of the maxillary tooth crowns in *Zhanghenglong* are nearly straight in some teeth but slightly sinuous in others (Fig. 6A). This condition is very similar to that in some hadrosaurids such as *Corythosaurus intermedius* (e.g., ROM 776) and *Brachylophosaurus canadensis* (e.g., CMN 8893). Mammilliform denticles occur along the mesial and distal margins of the crown (Fig. 6A). These marginal denticles are well-developed and equal in size. As in some derived basal hadrosauroids such as *Shuangmiaosaurus gilmorei* (LPM 0165), each alveolus has only one functional tooth for most of the tooth row, with the sporadic presence of two functional teeth in the middle of the occlusal surface [14], [53]. The worn surface of each functional tooth is slightly concave ventrally and strongly oblique lateroventrally in anterior view.

#### Dentary teeth (XMDFEC V0013)

The dental battery consists of 26 vertical tooth families. For most of the dental battery, two or three teeth are arranged dorsoventrally in each alveolus. There is one exception to the preceding condition: the 14th and 16th alveoli of the tooth row both bear four teeth, namely two replacement teeth and two functional teeth. Like the maxillary dentition, the dentary occlusal surface holds one worn tooth per alveolus in its anterior and posterior regions. Not surprisingly, the number of functional teeth in each alveolus keeps one or increases to two along the midsection of the tooth row, as in other basal hadrosauroids. Significantly, the 17th tooth position of the dental battery shows three functional teeth that contribute to the occlusal surface, similar to the condition seen in more derived hadrosaurids. It also accommodates four teeth. As is common for all hadrosauroids, the enameled lingual side of each unworn tooth crown is diamond-shaped and bisected by a conspicuous primary ridge [2], [4]. The primary ridge is very straight, crossing longitudinally the entire crown (Fig. 6B). For about 40% of all dentary teeth, the primary ridge is located along the midline of the lingual side of the crown, whereas in other dentary teeth, the primary ridge is slightly offset distally from the midline of the lingual side of the crown. The condition in *Zhanghenglong* may represent the intermediate form of the morphological change in the position of the primary ridge of the dentary tooth crown from basal hadrosauroids to more derived hadrosaurids. In most basal hadrosauroids such as *Bactrosaurus johnsoni* (AMNH 6553) and *Tethyshadros insularis* (SC 57026), the primary ridge of each dentary tooth is distally offset from the midline of the corresponding crown [1]–[3]. By contrast, in more derived hadrosaurids, most of the primary ridges of the crowns in the dentary tooth row are mesiodistally centered [2], [3], [47]. Although the enamel layer is incompletely preserved, evidence of faint ridges is apparent on the mesial half of the lingual side of each tooth crown (Fig. 6B). For each discernable tooth, one or two dorsoventrally elongate faint ridges are situated very close to the mesial margin of the crown. Each faint ridge is much thinner and shorter than the primary ridge. Owing to the highly eroded condition, no papillae are identified from the dentary dental battery. In lingual view, the tooth size progressively increases towards the middle region of the dental battery. The dentary tooth crowns have an approximate height/length ratio of 2.36. Similar to the typical growth pattern of the dentary dentition in iguanodontians, the teeth in the same row along the long axis of the dental battery progressively approach the occlusal surface posteriorly, and appear to erupt from back to front; alternate tooth families in the dental battery are subject to the same replacement cycle [55]. Each alveolus is defined anteriorly and posteriorly by two vertical dental laminas.

#### Dorsal vertebrae (XMDFEC V0014)

Five anterior-middle dorsal vertebrae are preserved in the plaster field jackets, containing the first dorsal vertebra (D1) and the fourth to seventh dorsal vertebrae (D4–D7). Linear measurements of these bones are shown in Table 1. D4 possibly underwent slight post-depositional deformation due to transverse crushing, and the measured height of its centrum is likely to be greater than the actual dorsoventral depth of the structure.

**Table 1:** Measurements for the anterior dorsal vertebrae (D1 and D4–D7) of *Zhanghenglong* (in mm).

Dorsal vertebra (XMDFEC V0014)	D1	D4	D5	D6	D7
1. anteroposterior length of the centrum	61	90	83	76	78
2. dorsoventral height of the centrum	70	71	73	75	74
3. mediolateral width of the centrum	78	64	70	72	73
4. proximodistal length of the transverse process	85	98	114	95	106
5. anteroposterior width of the neural spine	33	53	42	40	45
6. dorsoventral height of the neural arch	30	32	36	40	42
7. anteroposterior length of the neural arch	27	50	45	43	44

**Figure 7:**
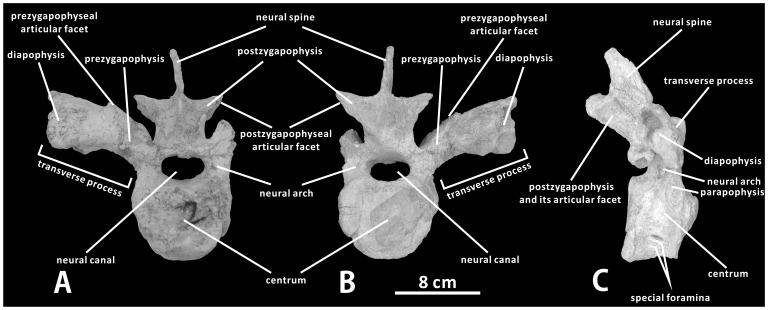
First dorsal vertebra of *Zhanghenglong yangchengensis*. D1 (XMDFEC V0014, paratype) in anterior (A), posterior (B), and right lateral (C) views.

As in some iguanodontian taxa in which the cervical and dorsal series are associated together, D1 shows the intermediate morphology between posterior cervical vertebrae and typical anterior dorsal vertebrae. It cannot be markedly differentiated from posterior cervical vertebrae in the following aspects: the centrum is strongly opisthocoelous in lateral view, and bears dorsoventrally short, oval anterior and posterior articular surfaces; the wide prezygapophysis extends dorsolaterally, and occupies the medial half of the slightly arched transverse process; the elongate postzygapophysis moderately arches posterolaterally and dorsally from the top of the neural arch; the neural spine is relatively short dorsoventrally (Fig. 7A–C). The vertebra also has some osteological features that are common for the dorsal series, such as the large, dorsomedially-facing articular facet of the prezygapophysis that is entirely higher than and located dorsolateral to the neural canal in anterior view, the gently concave parapophysis on the anterolateral side of the base of the neural arch, and the slightly shorter anteroposteriorly centrum (Fig. 7A, C). In anterior view, the centrum of D1 is about 1.6 times as tall as the paired neural arches; its posterior articular surface is slightly wider transversely than the anterior one. The lateral side of the centrum has prominent, broadly arcuate anterior and posterior edges, and is strongly concave anteroposteriorly in ventral view. The ventral half of the lateral side of the centrum is pierced by two small, oval foramina that face lateroventrally. The transversely narrow ventral side of the centrum is essentially flat, lacking any trace of the keel-like ridge. Dorsal to the centrum, the elliptical neural canal is surrounded predominantly by indistinguishably fused neural arches. The pedicles of paired neural arches gradually narrow dorsoventrally towards the sagittal plane, so that their ventromedial corners are triangular in shape and separated by the slightly concave dorsal surface of the centrum in the middle. Each neural arch measures 31 mm in total length, about half the anteroposterior length of the centrum. Only the right transverse process is accessible in D1. It projects dorsolaterally from the dorsolateral corner of the right neural arch to form the prezygapophysis, and nearly keeps horizontal along its lateral half (Fig. 7A, B). The angle between the long axis of the lateral half of the transverse process and that of the medial prezygapophysis is approximately 140°. The robust diapophysis for articulation with the tuberculum of the dorsal rib is located at the lateral extremity of the elongate transverse process. Dorsally, the paired postzygapophyses approach the relatively high neural spine with a short distance, and widely diverge posteriorly from the dorsoventral midpoint of the latter. Each postzygapophysis has a large, oval articular surface that faces ventrolaterally. The hooked neural spine of D1 is mediolaterally compressed and slightly tilted posterodorsally, which is about 1.2 times longer than the postzygapophysis. In contrast, the neural spine of D1 is proportionally shorter in some basal hadrosauroids such as *Eolambia caroljonesa* (CEUM 52173) and *Bactrosaurus johnsoni* (e.g., SBDE 95E5/15). In lateral view, the neural spine of D1 in *Zhanghenglong* tapers dorsally, and terminates into a blunt point (Fig. 7C).

**Figure 8:**
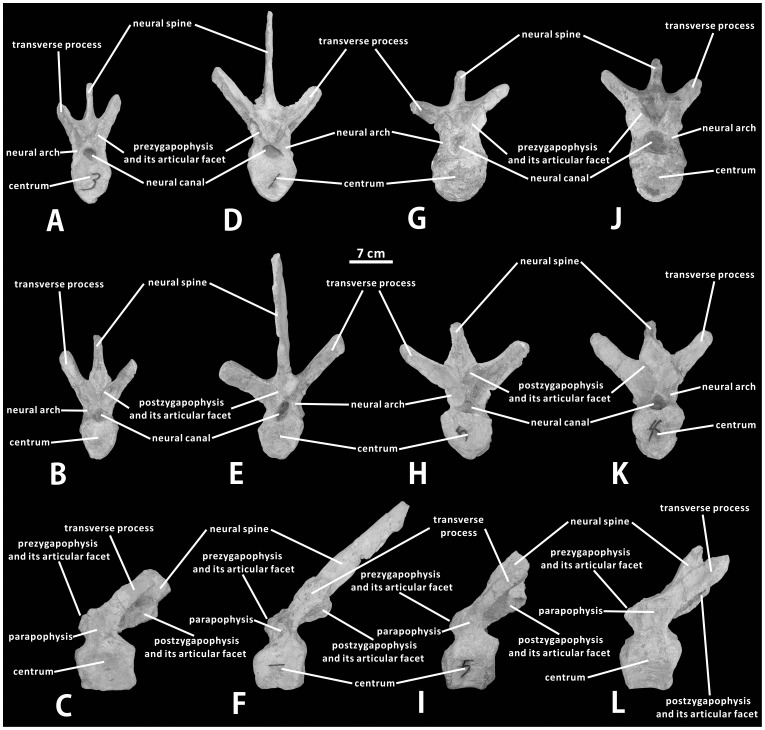
Anterior-middle dorsal vertebrae of *Zhanghenglong yangchengensis*. Four dorsal vertebrae probably representing D4–D7 orderly (XMDFEC V0014, paratype) in anterior (A, D, G, J), posterior (B, E, H, K), and left lateral (C, F, I, L) views.

The other four dorsal vertebrae in the paratype represent an incomplete anterior-middle dorsal series probably referable to D4–D7. The morphological features of these four dorsal vertebrae are very similar but distinct significantly from those of D1 (Fig. 8A–L). The centra are weakly opisthocoelous or nearly amphiplatyan in lateral view. They are anteroposteriorly longer than transversely wide and dorsoventrally high, and remain heart-shaped anteriorly and posteriorly. Similar to the condition observed in D1, two or three special foramina pierce the strongly concave anteroposteriorly lateroventral surface of each centrum. Ventrally, the left and right lateral sides of each centrum gradually converge downwards to form the median keel-shaped prominence. For each one in D4 to D7, vertical neural arches rise dorsally from laterodorsal regions of the centrum and partially enclose the circular neural canal. They are proportionally higher than those of D1. The average dorsoventral height of the paired neural arches in D4 to D7 is approximately 65% the average dorsoventral height of the corresponding centra. The strongly concave parapophysis has migrated dorsally to the dorsolateral side of the neural arch, and is located just below the base of the transverse process. The elongate transverse processes, which no longer bear the prezygapophyses medially, extend posterodorsally and laterally from the neural arch. They are straight and subtriangular in cross-section, and bear relatively blunt distal diapophyses. In lateral view, the base of the transverse process is sandwiched between the reduced prezygapophysis and postzygapophysis. In either one of D4 to D7, the truncated prezygapophyses arise from the anterodorsal region of the fused neural arches, and are placed more medially and anteriorly than those of D1. The elliptical articular facet of each prezygapophysis is still dorsomedially directed and steeply inclined dorsolaterally, with its long axis dorsolaterally-ventromedially oriented and slightly deflected posteriorly. As in other hadrosauroid taxa, the anterior margin of the prezygapophyseal articular facet is continuous with the anterior lamina of the transverse process. The paired postzygapophyses are slightly longer than the corresponding prezygapophyses; they project posterodorsally from the posteroventral corner of the neural spine. Like D1, the articular surfaces of the postzygapophyses in D4 to D7 are also ventrolaterally-facing and oval in shape. No neural spines are completely preserved in D4–D7. These partial neural spines are strongly canted posteriorly and mediolaterally compressed, showing subrectangular lateral profiles. Significantly, the neural spine of D5 is greatly elongate. It is about 4.3 times as tall as the corresponding centrum (Fig. 8D–F). The dorsoventrally high neural spines of anterior dorsal vertebrae can be also observed in some other iguanodontians, such as *Ouranosaurus nigeriensis* [51], *Saurolophus angustirostris* [56], and *Tsintaosaurus spinorhinus* [57]. Except for D1, the preserved anterior-middle dorsal series (D4–D7) reveals the following trends of morphological change in the dorsal series which are very common for other hadrosauroids: the strongly tilted posteriorly neural spine gradually becomes more vertical towards the sacrum; the posterodorsally and laterally directed transverse process progressively tends to project laterally towards the sacrum; the neurocentral suture and parapophysis become progressively higher posteriorly, and are accompanied by the elevation of the postzygapophysis; the slightly opisthocoelous centrum is transformed into the amphiplatyan centrum moving posteriorly (Fig. 8A–L) [57], [58].

#### Scapula (XMDFEC V0014)

**Figure 9:**
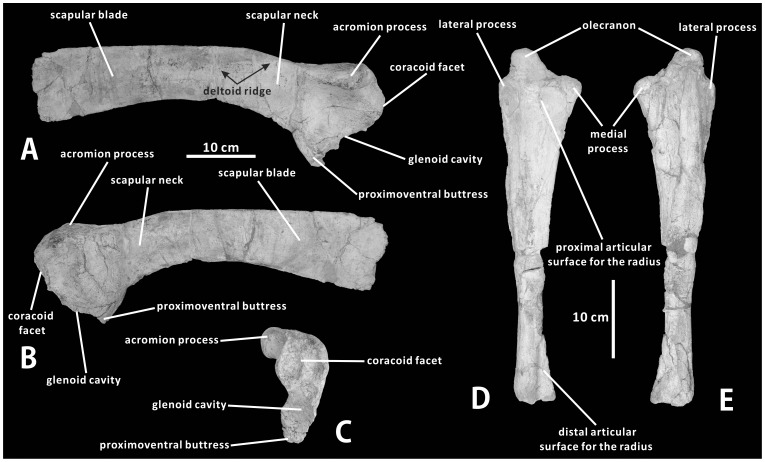
Scapula and ulna of *Zhanghenglong yangchengensis*. Right scapula (XMDFEC V0014, paratype) in lateral (A), medial (B), and proximal (C) views. Right ulna (XMDFEC V0014, paratype) in anterior (D) and posterior (E) views.

The scapula is a proximodistally elongate, blade-like bone that is slightly convex laterally so as to follow the general contour of the lateral side of the rib cage (Fig. 9A, B). The proximal end of the scapula is more expanded mediolaterally and dorsoventrally than the distal blade of the bone, and bears the coracoid facet and glenoid cavity along its anterior margin. The anteriorly-facing coracoid facet is rugose, gently concave and oval in shape. The facet is transversely wider and proximodistally shallower than the glenoid cavity, and is located just anterodorsal and slightly medial to the latter. The lunate glenoid cavity forms a relatively deep embayment that is moderately concave anteroventrally. It is supported ventrally by a stout proximoventral buttress. The enclosed rim of the glenoid cavity is considerably thinner than that of the coracoid facet. The glenoid cavity is 110% as dorsoventrally tall as the coracoid facet; in lateral view, these two articular surfaces meet at an angle of 145°. As in some basal hadrosauroid species such as *Tanius sinensis* (PMU R241) and *Bactrosaurus johnsoni* (e.g., AMNH 6553), the rod-shaped acromion process is anteroposteriorly directed and slightly recurved, where it slightly extends laterally from the proximodorsal border of the scapula (Fig. 9C); the anterior end of the acromion process is very robust and oriented anterodorsally (Fig. 9A). However, in hadrosaurines, the acromion process is unbent and relatively slender along its entire length [47], [59]. The proximal region of the scapula is broadly U-shaped in anterior view (Fig. 9C). Between the acromion process and the proximoventral buttress is a deep, subtriangular depression on the lateral surface of the proximal region of the bone. This depression may have served as the attachment area of the supracoracoideus muscle [60]. The medial surface of the proximal end of the scapula is strongly convex dorsoventrally and marked by fine anteroposteriorly oriented striations.

On the lateral side of the proximal half of the scapula, the acromion process extends posteroventrally to form a poorly defined deltoid ridge that is only incipiently developed. The arcuate deltoid ridge slightly bulges laterally, and gradually descends towards the midpoint of the ventral margin of the scapular blade. The condition is nearly identical to that of some basal hadrosauroids and most lambeosaurines, but differs from all hadrosaurines, in which a prominent deltoid ridge gradually broadens posteroventrally, and is continuous anterodorsally with the acromion process [3]. Proximally, the scapular neck is strongly constricted dorsoventrally. The dorsoventral depth of this constriction is about 50% of the maximum dorsoventral height of the proximal region of the scapula. The scapular blade is mediolaterally compressed and subrectangular in shape. The dorsal margin of the scapular blade is slightly convex, very similar to the condition observed in hadrosaurids. As in hadrosaurines, the dorsal and ventral margins of the distal region of the scapular blade are slightly divergent posteriorly. By contrast, the distal end of the scapula is bounded by nearly parallel dorsal and ventral borders in all lambeosaurine species except *Arenysaurus ardevoli* (MPZ 2008/333a–b), *Tsintaosaurus spinorhinus* (IVPP V725), and *Parasaurolophus cyrtocristatus* (FMNH P27393).

#### Ulna (XMDFEC V0014)

The ulna, which is one of the longest components of the forelimb, does not differ markedly from that seen in other hadrosauroids. The proximal end of the ulna is much more expanded mediolaterally than the distal end of the element. It consists of two pronounced processes and one subconical olecranon (Fig. 9D, E). Just below the olecranon, these two subtriangular processes, together with the proximal fourth of the shaft, partially enclose a mediolaterally wide and anteroposteriorly shallow concavity for reception of the proximoposterior portion of the radius (Fig. 9D). The larger medial process is anteromedially directed, whereas the weakly developed lateral process protrudes anterolaterally. The mediolateral width of the medial process is 55% greater than that of the lateral process. The stout olecranon projects dorsally from the proximoposterior region of the ulna. It is mediolaterally broad and anteroposteriorly constricted, and exhibits an elliptical outline in proximal view. The diaphysis of the ulna is straight in both anterior and posterior views, with a slightly sigmoid lateral or medial profile. The proximal end of the bone has a V-shaped cross-section that opens anteriorly. However, the distal outline of the element is subtriangular and medially tapering. Situated posterior to and partially shrouded by the medial process, a wedge-shaped, proximodistally directed groove occurs along the proximal half of the medial side of the ulnar shaft (Fig. 9E). This distinct groove becomes progressively narrower and shallower towards the mid-shaft, and possibly serves as the attachment area of the pronator quadratus muscle. A shallowly concave, triangular facet for articulation with the posterodistal region of the radius is present on the anteromedial surface of the distal third of the ulna (Fig. 9D). The bone has a smooth and convex distal surface that contacts the carpus. Compared to *Gilmoreosaurus mongoliensis* [53], the ulna of *Zhanghenglong* is relatively slender, with the proximodistal length/mediolateral width at mid-shaft ratio of 8.60.

## Discussion

### Confirmation of the Taxonomic Status in Light of Morphology

*Zhanghenglong yangchengensis* exhibits a series of plesiomorphic features typical of non-hadrosaurid hadrosauroids, including a large anterior foramen limited to the anterior half of the anterodorsal surface of the maxilla, a truncated and relatively low anterodorsal process of the maxilla, less than 30 tooth positions in the maxillary dental battery, a slightly expanded dorsoventrally anterior process of the jugal relative to the anterior neck of the bone, a nearly vertical coronoid process of the dentary, the dentary with less than 30 alveoli, the posterior terminus of the dentary tooth row overlapping the posterior margin of the coronoid process in medial view, one or two functional teeth in each alveolus of the dentary, and a strongly dorsoventrally-constricted scapular neck [1]–[3], [12], [53], [54]. Therefore, most of the material available for this genus and species unambiguously pertains to a non-hadrosaurid hadrosauroid.

*Zhanghenglong yangchengensis* also possesses some features rarely reported in basal hadrosauroids but observed in more derived hadrosaurids: the maxillary body is dorsoventrally tall; the elongate ectopterygoid ridge of the maxilla is straight and well-developed along its entire length; a lunate articular facet for the palatine occurs along the posterior border of the medial side of the jugal anterior process; the ventral edge of the jugal is strongly concave, forming a dorsoventrally deep embayment; the long axis of the dentary tooth row runs parallel with the lateral side of the dentary ramus; the median primary ridge of each maxillary tooth crown is either straight or slightly sinuous; the dentary shows a maximum of 4 teeth for each alveolus; the dorsal margin of the scapular blade is slightly convex [2], [3], [9], [47]. This taxon displays a unique combination of plesiomorphic and derived hadrosauroid features, revealing morphological transitions and the mosaic pattern of trait evolution from basal hadrosauroids to more derived hadrosaurids.

**Figure 10:**
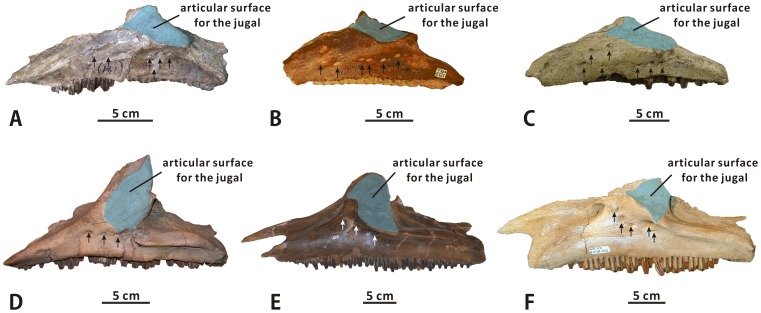
Comparison of the foramina on the lateral sides of the maxillae of some hadrosauroid species (all maxillae in lateral view). (A) *Bactrosaurus johnsoni* (AMNH 6553). (B) *Levnesovia transoxiana* (CCMGE 579/12457). (C) *Gilmoreosaurus mongoliensis* (AMNH FARB 30653, reversed). (D) *Amurosaurus riabinini* (AEHM 1/12). (E) *Edmontosaurus regalis* (CMN 2289). (F) *Brachylophosaurus canadensis* (MOR 1071-8-15-98-573). Positions of maxillary foramina are indicated by arrows.

**Figure 11:**
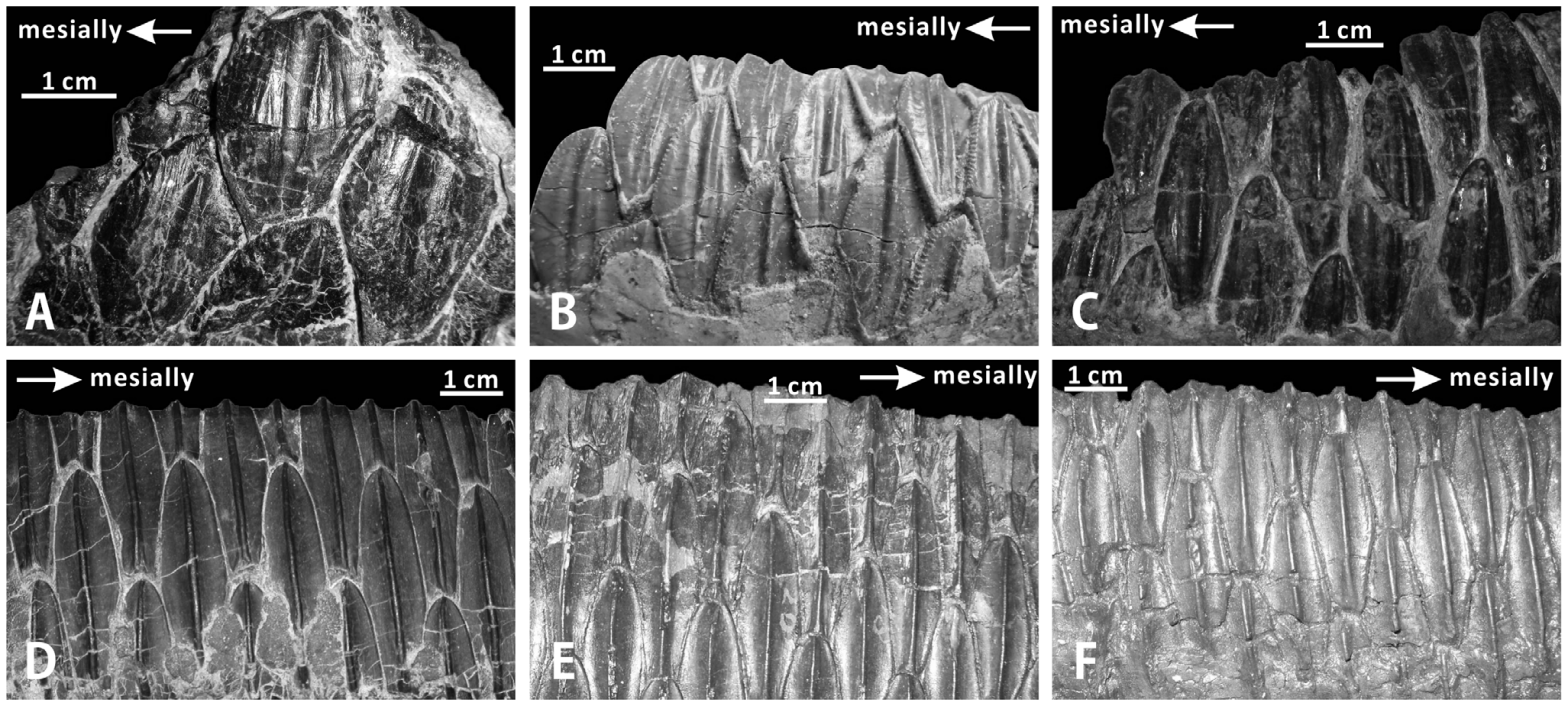
Comparison of the primary ridges of the tooth crowns in the dentary dental batteries of some hadrosauroid species (all dental batteries in lingual view). (A) *Equijubus normani* (IVPP V12534). (B) *Probactrosaurus gobiensis* (PIN 2232/42-1). (C) *Bactrosaurus johnsoni* (AMNH 6553). (D) *Parasaurolophus tubicen* (NMMNH P-25100). (E) *Edmontosaurus regalis* (CMN 2289). (F) *Brachylophosaurus canadensis* (CMN 8893).

Among the diagnostic characters of *Zhanghenglong yangchengensis*, a couple of transitional features between basal hadrosauroids and more derived hadrosaurids are particularly significant, including five maxillary foramina consisting of four small scattered ones arranged anteroposteriorly in a row and a large one close to the ventral extremity of the articular surface for the jugal, as well as dentary tooth crowns ornamented with both median and distally offset primary ridges. For the feature of the maxilla, it is not only reminiscent of basal hadrosauroids in which the maxillary foramina are ventrally positioned and anteroposteriorly aligned discontinuously on the lateral side of the element (Fig. 10A–C), but also apparently similar to hadrosaurids in which the posteriormost one or two of the dorsally positioned, closely aligned anterodorsally maxillary foramina are enlarged and located directly beneath the articular surface for the jugal (Fig. 10D–F). This configuration resembles the condition seen in *Tethyshadros insularis* (SC 57021) and *Telmatosaurus transsylvanicus* (FGGUB R1010). The other feature may represent the intermediate condition between the plesiomorphic and derived characters of hadrosauroids with regard to the position of the primary ridge of the dentary tooth crown. In most basal hadrosauroids, the primary ridge of each tooth in the dentary dental battery is located distal to the midline of the lingual side of the crown (Fig. 11A–C). Nevertheless, the median primary ridge for all or most of the teeth in the dentary dental battery is definitely characteristic of more derived hadrosaurids (Fig. 11D–F). These two conditions define two different character states used in some phylogenetic analyses of Hadrosauroidea (e.g., character 39 in [1], character 6 in [3], and character 7 in [59]).

In summary, *Zhanghenglong yangchengensis* is probably a non-hadrosaurid hadrosauroid based on a series of plesiomorphic features present in this taxon. The new genus and species significantly differs from other known members of Hadrosauroidea in having two distinct autapomorphies and a unique combination of features. Despite the confluence of some plesiomorphic features typical of non-hadrosaurid hadrosauroids, *Zhanghenglong* possesses some derived characters seen in hadrosaurids, as well as two transitional features that are intermediate between the corresponding plesiomorphic and derived characters of hadrosauroids. *Z. yangchengensis* may therefore represent a relatively derived non-hadrosaurid hadrosauroid, and is thought to be one of the closest relatives to Hadrosauridae.

### Statistical Examination using the MCA Method

**Figure 12:**
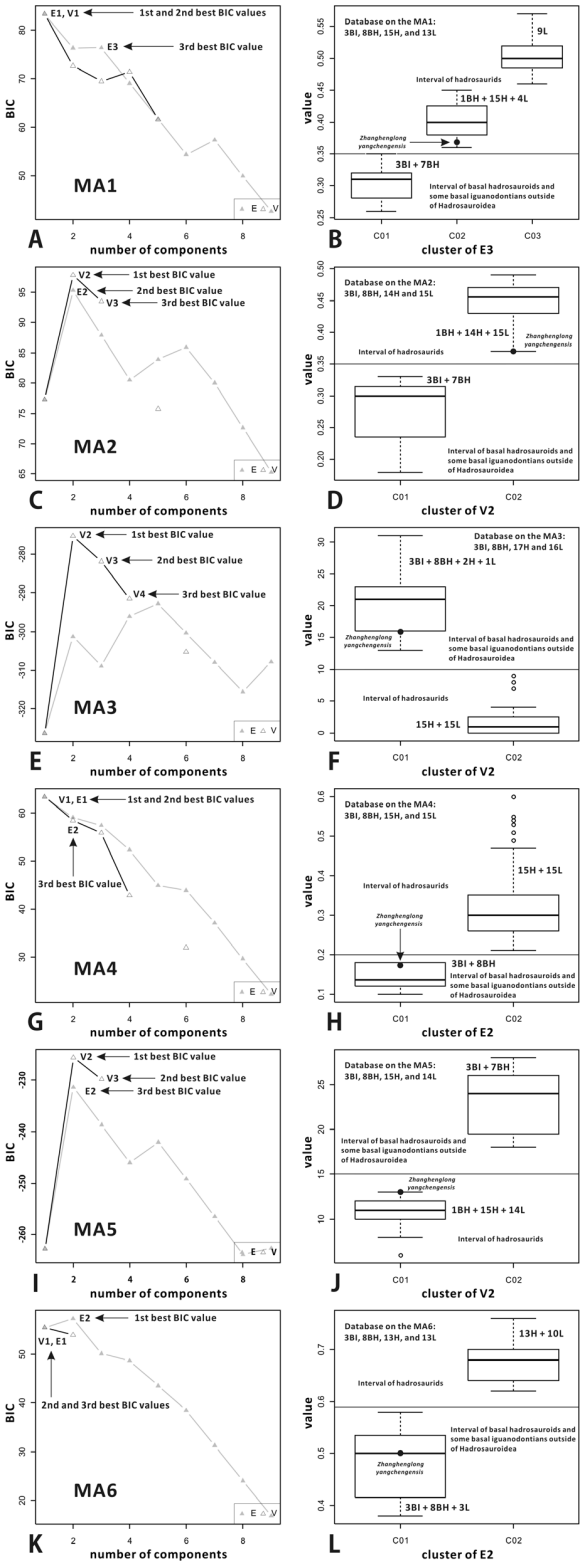
Bayesian Information Criterion (BIC) plots showing the results of model-based cluster analysis (MCA) implemented in Mclust for the datasets consisting of the values of most hadrosauroids and some basal iguanodontians on six selected measurement attributes (MA) derived from some phylogenetic characters, with the corresponding simplified box plots of the datasets showing the detached intervals respectively related to basal hadrosauroids and hadrosaurids. BIC plot (A) and box plot (B) for MA1. BIC plot (C) and box plot (D) for MA2. BIC plot (E) and box plot (F) for MA3. BIC plot (G) and box plot (H) for MA4. BIC plot (I) and box plot (J) for MA5. BIC plot (K) and box plot (L) for MA6. Abbreviations: BH, basal hadrosauroids; BI, basal iguanodontians outside of Hadrosauroidea; H, hadrosaurines; L, lambeosaurines.

To test the potential taxonomic status of *Zhanghenglong yangchengensis*, a quantitative analysis of one-dimensional measurement data of the taxon was carried out by means of measurement attributes (MA) selected from phylogenetic characters of hadrosauroids via MCA (see Supporting Information S2). Approximately 21% of the phylogenetic characters coded here (73 characters in Supporting Information S3) could be confidently considered to be the measurement attributes for MCA. The dataset of each measurement attribute consists of the values of almost all hadrosauroid species except *Z. yangchengensis*, as well as those of three iguanodontians closely related to Hadrosauroidea (*Iguanodon bernissartensis*, *Mantellisaurus atherfieldensis*, and *Ouranosaurus nigeriensis*). Given that *Z. yangchengensis* is inferred to be a derived non-hadrosaurid hadrosauroid, we have chosen some particular measurement attributes for quantitative testing. For each one of these attributes, the subsets yielded by the partition of the database using MCA reveal a distinct quantitative disparity between basal hadrosauroids and more derived hadrosaurids. Some of the selected measurement attributes are available for *Z. yangchengensis*; they are referable to the following phylogenetic characters in Supporting Information S3: the ratio between the maximum dorsoventral height and anteroposterior length of the maxilla (character 111; MA1), ratio between the anteroposterior length of the ectopterygoid shelf and that of the maxillary tooth row (character 112; MA2), angle between the ectopterygoid ridge and the ventral margin of the posterior portion of the maxilla (character 113; MA3), ratio between the anteroposterior length of the edentulous region and that of the tooth row of the dentary (character 38; MA4), angle between the medial border of the dentary symphyseal process and the lateral surface of the dentary ramus (character 44; MA5), and ratio between the dorsoventral depth of the scapular neck and the maximum dorsoventral height of the scapular proximal end (character 261; MA6) (Fig. 12A–L).

The partitioned datasets of selected measurement attributes provide standards for the quantitative evaluation of one-dimensional measurement data on osteological characters of *Zhanghenglong yangchengensis*. In each standard, the two detached intervals of the database on the corresponding measurement attribute are adequately representative of the distribution ranges of the values of non-hadrosaurid iguanodontians and hadrosaurids, respectively. For MA3, MA4, and MA6, the value of *Z. yangchengensis* falls within the interval representing basal hadrosauroids and some iguanodontians outside of Hadrosauroidea. By contrast, for MA1, MA2, and MA5, the value for this species is restricted to the interval that represents hadrosaurids. Consequently, the data of *Z. yangchengensis* on selected measurement attributes straddle the two combinations of intervals of partitioned datasets respectively related to basal hadrosauroids and more derived hadrosaurids (Table 2). This condition is apparently similar to the mosaic pattern of trait evolution from basal hadrosauroids to more derived hadrosaurids present in the specimens of the species, and corroborates the preceding inference made from morphology. Based on the result of the quantitative analysis and evidence from morphology, we argue that *Z. yangchengensis* probably represents a relatively derived non-hadrosaurid hadrosauroid dinosaur closely related to Hadrosauridae.

**Table 2:** Relevant data of *Zhanghenglong* and detached intervals of the datasets consisting of the values of most hadrosauroids and some basal iguanodontians on selected measurement attributes*.

Measurement attribute (XMDFEC V0013 and V0014)	Value of *Zhanghenglong*	Interval related to basal hadrosauroids	Interval related to hadrosaurids
1. ratio between maximum dorsoventral height and anteroposterior lengthof maxilla	0.37	up to 0.35	greater than 0.35
		C01: [0.26, 0.33]	C02+ C03: [0.36, 0.57]
2. ratio between anteroposterior length of ectopterygoid shelf and that ofmaxillary tooth row	0.37	up to 0.35	greater than 0.35
		C01: [0.20, 0.33]	C02: [0.38, 0.49]
3. angle between ectopterygoid ridge and ventral margin of posteriorportion of maxilla	16°	greater than 10°	up to 10°
		C01: [13°, 31°]	C02: [0°, 9°]
4. ratio between anteroposterior length of edentulous region and that oftooth row of dentary	0.17	up to 0.20	greater than 0.20
		C01: [0.10, 0.18]	C02: [0.21, 0.60]
5. angle between medial border of dentary symphyseal process and lateralsurface of dentary ramus	13°	greater than 15°	up to 15°
		C02: [18°, 28°]	C01: [8°, 13°]
6. ratio between dorsoventral depth of scapular neck and maximum dorsoventralheight of scapular proximal end	0.50	up to 0.59	greater than 0.59
		C01: [0.38, 0.58]	C02: [0.60, 0.76]

*the boundary value between the two intervals was estimated to be the average value of the greatest value from the former component with low values and the least value from the latter component with high values.

C, the component resulting from MCA of the database consisting of the sample means of all measured taxa.

### Phylogenetic Analysis

A comprehensive phylogenetic analysis of Hadrosauroidea was conducted in order to assess the systematic position of *Zhanghenglong yangchengensis* and construct an overall tree topology on the phylogeny of basal hadrosauroids. The data matrix used in this analysis includes 61 species-level taxonomic units (4 iguanodontians outside of Hadrosauroidea and 57 hadrosauroids) coded across 346 equally-weighted characters (see Supporting Information S3 and Supporting Information S4). These applicable characters consist of 235 cranial and 111 postcranial components. Most of them were culled or modified from some recently published character lists for phylogenetic analyses of Hadrosauroidea, especially You et al. [1], Horner et al. [2], Evans and Reisz [9], Godefroit et al. [61], Prieto-Márquez and Wagner [47], and Xing et al. [59]. 10 new characters explicated in Supporting Information S3 were also added to the data matrix used here. The non-hadrosauroid iguanodontian *Ouranosaurus nigeriensis* was appointed the single outgroup, due to its strong affinities with Hadrosauroidea. In addition, we have adequately revised the previously published data on the character coding of some hadrosaurid species (including *Charonosaurus jiayinensis*, *Lophorothon atopus*, and *Nipponosaurus sachalinensis*) because the ascription of the available material for each of these taxa is highly contentious (see Supporting Information S4). The databases of *Eolambia caroljonesa* and *Magnapaulia laticaudus* used for the phylogeny were also updated in light of the new morphological information mentioned in relevant literature [54], [62]. Finally, *Hadrosaurus foulkii* is excluded from the data matrix used in this paper because this taxon is considered a *nomen dubium* that lacks any diagnostic characters [63].

The data matrix of 346 characters and 61 taxa was analyzed using the maximum parsimony method in the program TNT version 1.0 [35]. Prior to the traditional search, 10000 trees were set as maximum trees in memory. A heuristic search with 1000 replicates of Wagner trees using random addition sequences was performed, followed by the TBR branch swapping holding 1000 trees per replicate. The support of clades was evaluated by standard absolute frequencies in 1000 replicates of bootstrap analysis and decay indices of Bremer support (bootstrap and Bremer decay values).

**Figure 13:**
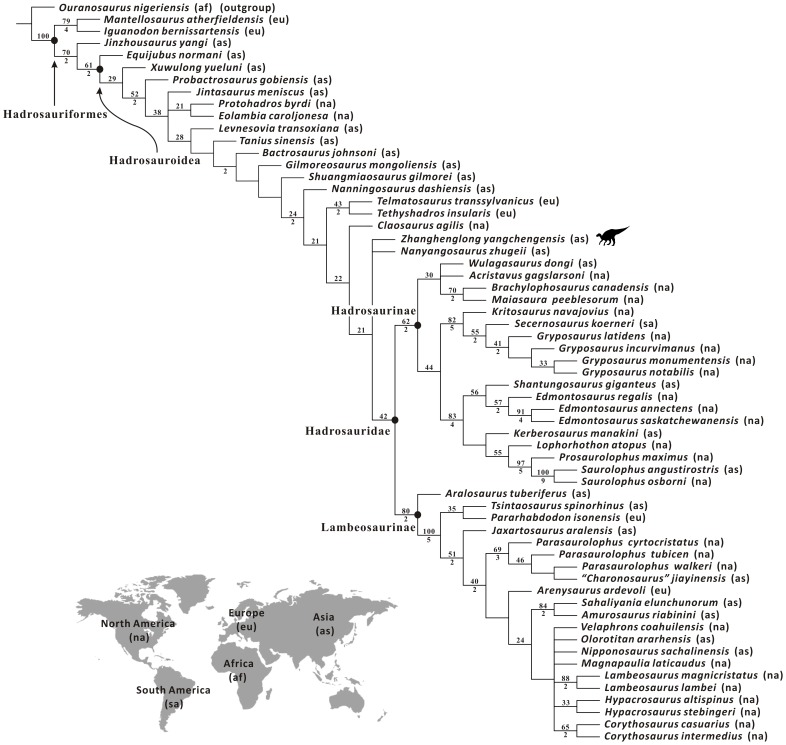
Strict consensus of 54 most parsimonious trees resulting from the maximum parsimony analysis implemented in the software TNT, showing the suggested systematic position of *Zhanghenglong yangchengensis* within Hadrosauroidea. Numbers above nodes represent bootstrap values, whereas those beneath nodes represent Bremer decay values. Bootstrap values lower than 20 and Bremer decay values less than 2 are not shown.

**Figure 14:**
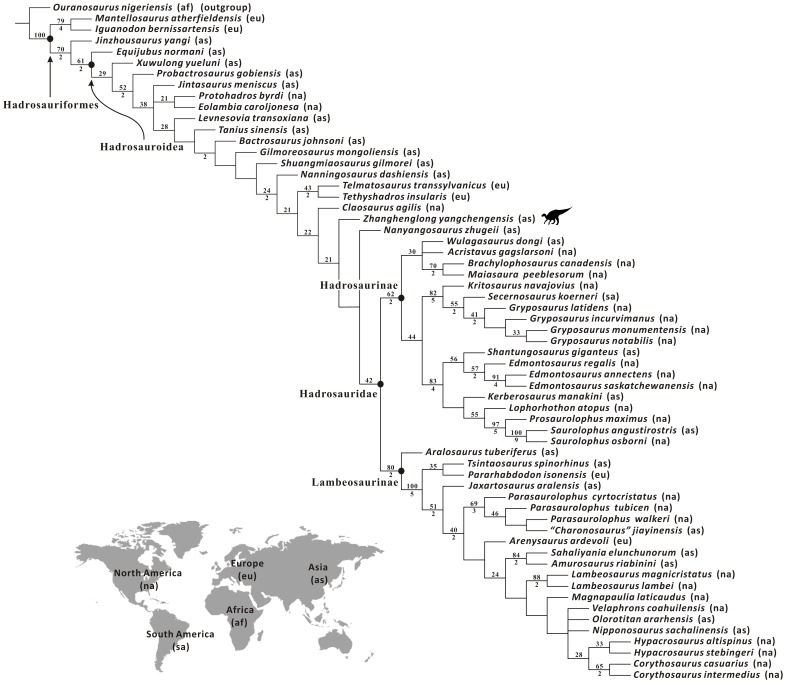
50% majority rule consensus of 54 most parsimonious trees produced by the maximum parsimony analysis implemented in the software TNT, showing the potential phylogenetic position of *Zhanghenglong yangchengensis* within Hadrosauroidea. Numbers above nodes represent bootstrap values, whereas those beneath nodes represent Bremer decay values. Bootstrap values lower than 20 and Bremer decay values less than 2 are not shown.

The maximum parsimony analysis resulted in 54 most parsimonious trees (MPTs), with a tree length of 1004 steps, a consistency index (CI) of 0.495 and a retention index (RI) of 0.863. Fig. 13 shows the strict consensus tree derived from the 54 MPTs. The tree topology of the strict consensus indicates that *Zhanghenglong yangchengensis*, *Nanyangosaurus zhugeii*, and Hadrosauridae together constitute a monophyletic group with an unresolved polytomy, which is weakly supported by the low bootstrap (21%) and Bremer decay (1) values. This clade is diagnosed by five unambiguous synapomorphies, although only three of these features are currently known from *Z. yangchengensis*: the median primary ridges available for all or most of the tooth crowns in the dentary dental battery (character 7^1^), the ratio between the vertical distance from the apex of the maxillary dorsal process to the maxillary ventral margin and the anteroposterior length of the maxillary ventral margin greater than 0.35 and up to 0.45 (character 111^1^), and the arcuate dorsal margin of the scapula in lateral view (character 256^1^) (see Supporting Information S5). *N. zhugeii* and *Z. yangchengensis* are depicted as having the closest affinities with Hadrosauridae. For the 50% majority rule consensus tree, *Z. yangchengensis* is positioned as the sister taxon to the clade consisting of *N. zhugeii* and Hadrosauridae (Fig. 14); the phylogenetic relationships among the three groups are very similar to but slightly more complicated than those of the strict consensus. In all MPTs and the strict consensus tree, *Z. yangchengensis* is placed in a relatively derived position within the paraphyletic group that represents basal hadrosauroids. This phylogenetic hypothesis is very consistent with the inferences drawn from the morphological comparisons and quantitative analysis of measurement data.

The tree topology of basal hadrosauroids in the strict consensus is generally identical to that in the 50% majority rule consensus (Figs. 13 and 14). The former is briefly described as follows. Hadrosauroidea is defined as the least inclusive clade containing *Equijubus normani* and *Parasaurolophus walkeri*, following You et al. [1]. *Xuwulong yueluni* is recovered as slightly more derived than *E. normani*. *Eolambia caroljonesa* and *Protohadros byrdi* together form a North American clade of basal hadrosauroids that is more derived than *Probactrosaurus gobiensis*. These two species are united based on three unequivocal synapomorphies, including the absence of the strongly curved upwards posterior process of the surangular (character 64^0^) and wider infratemporal margin of the jugal relative to the orbital margin of the bone (character 127^2^). There is a polytomy at the base of Hadrosauroidea which involves *Jintasaurus meniscus*, the preceding clade consisting of *E. caroljonesa* and *P. byrdi*, and the least inclusive clade containing *Levnesovia transoxiana* and *Edmontosaurus regalis*. *L. transoxiana* seems to be more basal than *Tanius sinensis* and *Bactrosaurus johnsoni*, although these species share strong similarities in cranial morphology [22]. *Gilmoreosaurus mongoliensis* occupies a more highly nested position than *B. johnsoni*. *Shuangmiaosaurus gilmorei* is recovered as slightly more derived than *G. mongoliensis* but slightly more basal than *Nanningosaurus dashiensis*. Similar to the systematic position of *Claosaurus agilis* presented in the strict consensus tree, *N. dashiensis* is recovered as a derived non-hadrosaurid hadrosauroid. *Telmatosaurus transsylvanicus* is found to be the sister taxon of *Tethyshadros insularis*. These two taxa constitute the single European clade of basal hadrosauroids, which is depicted as slightly more derived than *N. dashiensis* but slightly more basal than *C. agilis*. This European clade is diagnosed by the following unambiguous synapomorphies: bamboo leaf-like marginal denticles along the dorsal half of the dentary tooth crown (character 11°), saw-toothed marginal denticles along the ventral half of the maxillary tooth crown (character 23°), and the angle between the slope of the dentary anterior end which contacts the predentary and the horizontal greater than 130° (character 40°). *C. agilis*, one of the closest relatives to Hadrosauridae, is depicted as more basal than *Zhanghenglong yangchengensis*, *Nanyangosaurus zhugeii*, and Hadrosauridae.

For the tree topology of Hadrosaurinae in the strict consensus, our present analysis does not significantly alter the relevant result argued by Xing et al. [59]. There is one exception to the condition: *Lophorothon atopus* is placed as the sister group to the clade composed of the genera *Prosaurolophus* and *Saurolophus* (Fig. 13), based on the anatomical data of the holotype of this species [64]. The posterior fragments of the paired nasals are well preserved in the holotype of *L. atopus*. Either of the fragments shows evidence of a dorsoventrally low, strongly invaginated circumnarial fossa with a thick, right-angled posterodorsal margin (character 163^2^), which is typically seen in the genera *Prosaurolophus* and *Saurolophus*. However, the solid nasal crest of the species is only incipiently developed, compared with that of the genera *Prosaurolophus* (the relatively short, moderately developed nasal crest bounded posteriorly by the underlying frontal buttress) and *Saurolophus* (the elongate, well developed nasal crest overhanging the occiput). Given the incompletely fused calvarial sutures and relatively small size of the holotype of *L. atopus*, the hypothesis that *L. atopus* may represent the juvenile form of *Prosaurolophus maximus* cannot be rejected.

The strict consensus tree has a large polytomy involving numerous helmet-crested lambeosaurines (Fig. 13). Therefore, the phylogenetic relationships among helmet-crested lambeosaurines are not fully resolved. Within the unresolved polytomy, the monophyly of the genus *Hypacrosaurus* is corroborated again after the work by Evans [48]. In the more complex 50% majority rule consensus tree (Fig. 14), the sister group relationship between the genera *Corythosaurus* and *Hypacrosaurus* is weighed by the relatively low support values (bootstrap value = 28%; Bremer decay value = 1). Furthermore, the presumptive systematic positions of the lambeosaurines *Arenysaurus ardevoli* and *Charonosaurus jiayinensis* in the strict consensus tree also deserve special attention. The European lambeosaurine *A. ardevoli* is placed as the sister group to the clade consisting of all known helmet-crested lambeosaurines. *C. jiayinensis* is nested within the monophyletic genus *Parasaurolophus*. This species is recovered as the sister taxon to *Parasaurolophus walkeri*, and is depicted as more closely related to *Parasaurolophus tubicen* than to *Parasaurolophus cyrtocristatus*. Consequently, *C. jiayinensis* possibly represents the fourth valid species of the genus *Parasaurolophus*.

Although *Hadrosaurus foulkii* is considered a *nomen dubium* herein, Prieto-Márquez [65] regarded this taxon as a valid species with a combination of plesiomorphic and derived appendicular characters, which was placed at the base of Hadrosauridae. We therefore conducted another maximum parsimony analysis including *H. foulkii*, in order to test the systematic postion of *Zhanghenglong yangchengensis* shown in Fig. 13. Rerunning the analysis after the participation of *H. foulkii* produced 486 MPTs of 1012 steps. This analysis does not significantly alter the phylogenetic position of *Z. yangchengensis* (see Supporting Information S5, Fig. 2). In the strict consensus tree derived from the 486 MPTs, *H. foulkii* is recovered as more basal than *Z. yangchengensis* and *Nanyangosaurus zhugeii*.

**Figure 15:**
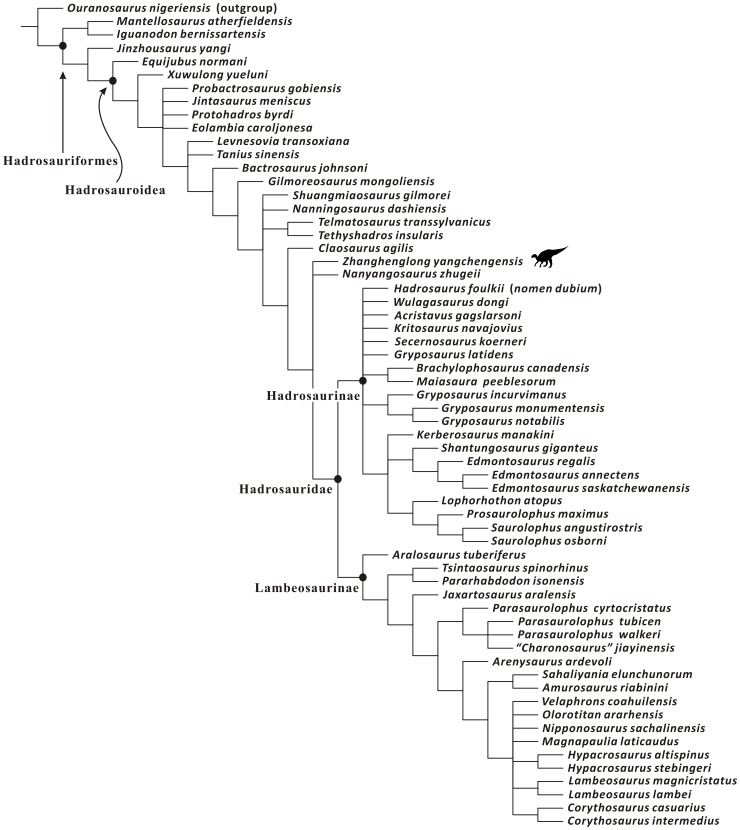
Strict consensus of 405 most parsimonious trees recovered from the cladistic analysis of Hadrosauroidea, with the participation of *Hadrosaurus foulkii* (the characters associated with the laterodistal corner of the deltopectoral crest were coded as polymorphic for *Brachylophosaurus*, *Shantungosaurus*, and *Edmontosaurus*).

The material of *Hadrosaurus foulkii* consists of several poorly preserved fragments of the cranium and a partial postcranial skeleton [4], [63]. Most of the phylogenetically informative characters of the taxon come from the postcranial elements, including the coracoid, humerus, ilium, and ischium. Except for the wide arcuate laterodistal corner of the deltopectoral crest, the vast majority of the features in *H. foulkii* are typical of *Edmontosaurus* and *Shantungosaurus* (see Supporting Information S4). Prieto-Márquez [65] discussed the wide arcuate laterodistal corner of the deltopectoral crest seen in *H. foulkii* and basal hadrosauroids, and considered it to be plesiomorphic for Hadrosauroidea, in comparison with the angular laterodistal corner of the deltopectoral crest common among most hadrosaurids. However, this configuration is also observed in a few humeri from the hadrosaurid genera *Brachylophosaurus* (e.g., CMN 8893), *Shantungosaurus* (e.g., GMV 1780-48), and *Edmontosaurus* (e.g., USNM 2414), which could be interpreted as intraspecific variation. Here we argue that the wide arcuate laterodistal corner of the deltopectoral crest may disturb the accurate character coding of *H. foulkii*, such as the measurement taken for the proximodistal length of the deltopectoral crest in character 269. In fact, the coding of the four characters associated with the laterodistal corner of the deltopectoral crest (characters 267, 269, 271, and 272) plays an important role in placing *H. foulkii* outside of Hadrosauridae (see Supporting Information S5, Fig. 2). When these four characters are coded as polymorphic for *Brachylophosaurus*, *Shantungosaurus*, and *Edmontosaurus*, the alternate phylogeny indicates that *H. foulkii* is definitely a member of the monophyletic group that consists of all non-lambeosaurine hadrosaurids (Fig. 15; Supporting Information S5, Fig. 3). Therefore, *H. foulkii* is considered here a *nomen dubium* owing to a lack of diagnostic characters, and may represent an enigmatic member of the monophyletic group that consists of all non-lambeosaurine hadrosaurids (i.e. Hadrosaurinae). This is why we adhere to the traditional taxonomy of Hadrosauridae argued by Horner et al. [2], where Hadrosauridae is divided into two major clades: Hadrosaurinae and Lambeosaurinae.

### Biogeographic Implications

In current iguanodontian research, the issue of the geographic origin of Hadrosauridae has long been debated, and refers to two proposed hypotheses, an Asian or North American origin of Hadrosauridae [66]. Our cladogram indicates that the two Asian basal hadrosauroids *Zhanghenglong yangchengensis* and *Nanyangosaurus zhugeii* represent the closest relatives to Hadrosauridae (Fig. 13). Until now, the first appearance of lambeosaurine species in the stratigraphic record is represented by *Jaxartosaurus aralensis* from the middle Santonian to earliest Campanian Syuk-Syuk Formation [67], [68]. The stratigraphic age of the oldest hadrosaurine *Gryposarus latidens* was estimated to be the late Santonian to early Campanian [49]. Therefore, the most recent common ancestor of Hadrosauridae is very likely to have split into Lambeosaurinae and Hadrosaurinae no later than the early Santonian. This temporal interval is quite close to the stratigraphic age of *Z. yangchengensis*, which is thought to be the middle Santonian [31], [33]. The other basal hadrosauroid closely related to Hadrosauridae, *N. zhugeii*, was recovered from the early to middle Coniacian lower portion of the Xiaguan Formation in the Xiaguan-Gaoqiu Basin of southwestern Henan Province [69]. The stratigraphic age of this species is slightly older than the deadline of the presumptive temporal interval when the split of Hadrosauridae into Hadrosaurinae and Lambeosaurinae occurred. Thus, these two basal hadrosauroids, both from the Upper Cretaceous of southwestern Henan Province, could be considered to be the known closest relatives of Hadrosauridae, supporting the Asian origin hypothesis.

Because most basal lambeosaurines (*Aralosaurus tuberiferus* [70], *Tsintaosaurus spinorhinus* [57], and *Jaxartosaurus aralensis* [71]) are found in Asia, the geographic origin of Lambeosaurinae is probably restricted to this continent. On the other hand, three of the four major groups within Hadrosaurinae have the first divergent Asian species (Fig. 13), including *Wulagasaurus dongi* [61], *Shantungosaurus giganteus* [72], and *Kerberosaurus manakini* [73]. Except for the first divergent Asian species, each of these three groups includes at least three North American species. This phenomenon could be interpreted as the dispersal of Asian hadrosaurine clades to North America for further radiation. However, the possibility that Hadrosaurinae originated in North America and subsequently migrated to Asia cannot be ruled out. Combining the phylogenetic and stratigraphic data on *Zhanghenglong yangchengensis* with the biogeographical inferences on Hadrosaurinae and Lambeosaurinae has yielded support for the hypothesis that Hadrosauridae might have originated in Asia. Although the discovery of *Z. yangchengensis* provides direct evidence for the Asian origin of Hadrosauridae, the preceding hypothesis still requires further testing.

## Supporting Information

Supporting Information S1 **Measurements of selected skeletal elements of** ***Zhanghenglong yangchengensis*** **and some hadrosaur relatives.**
(DOC)

Supporting Information S2 **Datasets on the selected measurement attributes for model-based clustering.**
(DOC)

Supporting Information S3 **Characters used in the phylogenetic analysis of Hadrosauroidea.**
(DOC)

Supporting Information S4 **Character-taxon matrix used in the phylogenetic analysis of Hadrosauroidea.**
(DOC)

Supporting Information S5 **Original information in the analytical process of the program TNT, with the strict consensus tree and list of synapomorphies.**
(DOC)
