# Supplementary material for: A New Basal Hadrosauroid Dinosaur (Dinosauria: Ornithopoda) with Transitional Features from the Late Cretaceous of Henan Province, China
Source: PLoS One. 2014 Jun 5;9(6):e98821. doi: 10.1371/journal.pone.0098821 (PMC4047018; doi:10.1371/journal.pone.0098821)
Supplement: Supporting Information S1 — Measurements of selected skeletal elements of Zhanghenglong yangchengensis and some hadrosaur relatives. (DOC) [file pone.0098821.s001.doc]

**Supporting Information S1**

**Measurements of Selected Skeletal Elements of *Zhanghenglong yangchengensis* and Some Hadrosaur Relatives**

Here we present a set of measurement and observation data on some selected elements of the holotype and paratype of *Zhanghenglong yangchengensis* which contains the maxilla, dentary, dentary teeth, and scapula. The measurement data are referable to both linear and angular measurements for the relevant bones. Most of the linear measurements are used to perform ratio calculations so as to conduct better mathematical comparisons of osteological features among *Zhanghenglong* and some hadrosauroid relatives. A small number of raw data were obtained from plates in the literature and digital images from academic exchanges, rather than personal examinations and measurements. They are clearly annotated in boldface type. The accuracy of these data might be problematic, which is probably attributed to small errors yielded by mathematical conversions using the scale bar.

**Institutional abbreviations**. **AEHM**, Amur Natural History Museum, Blagoveschensk, Russia; **AMNH**, American Museum of Natural History, New York, USA; **CEUM**, College of Eastern Utah Prehistoric Museum, Price, USA; **GMH**, Geological Museum of Heilongjiang Province, Harbin, China; **IRSNB**, Institut Royal des Sciences Naturelles de Belgique, Brusels, Belgium; **IVPP**, Institute of Vertebrate Paleontology and Paleoanthropology, Beijing, China; **MOR**, Museum of the Rockies, Bozeman, Montana, USA; **NMMNH**, New Mexico Museum of Natural History and Science, Albuquerque, USA; **PIN**, Paleontological Institute of the Russian Academy of Sciences, Moscow, Russia; **PMU**, Museum of Evolution, Uppsala University, Uppsala, Sweden; **SMU**, Shuler Museum of Paleontology, Southern Methodist University, Dallas, USA; **XMDFEC**, Xixia Museum of Dinosaur Fossil Eggs of China, Xixia, China.

Table 1 Measurements of the maxilla in *Zhanghenglong* and some other hadrosauriforms (mm)*

| Maxilla | *Zhanghenglong yangchengensis* | *Parasaurolophus tubicen* | *Brachylophosaurus canadensis* | *Protohadros*  *byrdi* | *Gilmoreosaurus mongoliensis* |
| --- | --- | --- | --- | --- | --- |
|  | XMDFEC V0013 | NMMNH P-25100 | MOR 1071-8-15-98-573 | SMU 74582 | AMNH 6551 |
| 1. anteroposterior length | 230.0 | 358.0 | 309.0 | **378.0** | 272.0 |
| 2. dorsoventral height | 85.0 | 205.5 | 111.0 | **117.3** | 87.0 |
| 3. length of the ectopterygoid ridge | 82.5 | 157.0 | 142.0 | **113.5** | 79.5 |
| 4. length of the dorsal margin of the anteroventral process | 138.0 | 251.0 | 148.5 | **215.4** | 153.0 |
| 5. horizontal distance from the apex of the dorsal ramus to the anteriormost end of the maxilla | 163.0 | 247.0 | 158.0 | **257.0** | 174.0 |
| ratio |  |  |  |  |  |
| (1) 2:1 | 0.37 | 0.57 | 0.36 | 0.31 | 0.32 |
| (2) 3:1 | 0.36 | 0.44 | 0.46 | 0.30 | 0.29 |
| (3) 4:1 | 0.60 | 0.70 | 0.48 | 0.57 | 0.56 |
| (4) 5:1 | 0.71 | 0.69 | 0.51 | 0.68 | 0.64 |
| angle |  |  |  |  |  |
| (1) angle between the dorsal margin of the anteroventral process and the level | 32º | 33º | 34º | **33º** | 32º |
| (2) angle between the level and the ectopterygoid ridge | 16º | 1º | 9º | **22º** | 17º |

*The boldface fonts indicate the measurement data from the plates in the literature.

Table 2 Measurements of the dentary in *Zhanghenglong* and some other hadrosauriforms (mm)*

| Dentary | *Zhanghenglong yangchengensis* | *Sahaliyania elunchunorum* | *Prosaurolophus maximus* | *Eolambia caroljonesa* | *Bactrosaurus johnsoni* |
| --- | --- | --- | --- | --- | --- |
|  | XMDFEC V0013 | GMH W451 | MOR 447 | CEUM 34357 | AMNH 6553 |
| 1. anteroposterior length | 364.0 | 399.0 | 519.0 | **220.0** | 224.0 |
| 2. length of the dental battery | 247.0 | 270.0 | 372.0 | **164.0** | 176.0 |
| 3. length of the diastema | 42.0 | 84.0 | 98.0 | **30.0** | 28.0 |
| ratio |  |  |  |  |  |
| (1) 2:1 | 0.68 | 0.68 | 0.72 | 0.75 | 0.79 |
| (2) 3:1 | 0.12 | 0.21 | 0.19 | 0.14 | 0.13 |
| (3) 3:2 | 0.17 | 0.31 | 0.26 | 0.18 | 0.16 |
| angle |  |  |  |  |  |
| (1) inclination angle of the diastema relative to the level | 152º | 140º | 154º | **146º** | 153º |
| (2) angle of the downward deflection of the dentary rostral end | 15º | 38º | 22º | **16º** | 18º |
| (3) angle between the medial margin of the dentary symphysis and the lateral surface of the dentary body | 13º | 11º | 12º | **25º** | 22º |
| (4) angle between the slope of the dentary rostral region and the horizontal | 113º | 113º | 134º | **117º** | 109º |
| (5) anterior inclination angle of the coronoid process relative to the level | 90º | 76º | 74º | **90º** | 90º |

*The boldface fonts indicate the measurement data from the plates in the literature.

Table 3 Measurements and comparisons of the dentary teeth in *Zhanghenglong* and some other hadrosauriforms (mm)*

| Dentary teeth | *Zhanghenglong yangchengensis* | *Equijubus*  *normani* | *Gryposaurus latidens* | *Edmontosaurus regalis* | *Iguanodon bernissartensis* |
| --- | --- | --- | --- | --- | --- |
|  | XMDFEC V0013 | IVPP V12534 | AMNH 5465 | CMN 2289 | IRSNB 1561 |
| 1. apicobasal height of the crown | 35.0 | 29.0 | 30.5 | 34.0 | **41.0** |
| 2. mesiodistal width of the crown | 14.8 | 18.0 | 16.5 | 11.5 | **27.0** |
| ratio |  |  |  |  |  |
| 1:2 | 2.36 | 1.60 | 1.85 | 2.95 | 1.52 |
| angle |  |  |  |  |  |
| (1) angle between the mesial and distal margins of the dorsal half of the crown | 26º | 45º | 31º | 24º | **50º** |
| (2) angle between the occlusal plane of the dental battery and horizontal | 17º | 15º | 20º | 19º | **10º** |
| character |  |  |  |  |  |
| (1) number of teeth per cm | 0.68 | 0.56 | 0.61 | 0.87 | **0.37** |
| (2) number of primary ridges (a), secondary ridges (b), and faint ridges (c) of one crown | 1a+2c | 1a+1b+3c | 1a | 1a | **1a+1b+3c** |
| (3) maximum number of the teeth per alveolus | 4 | 3 | 5 | 6 | **2** |
| (4) maximum number of the functional teeth on the occlusal plane per alveolus | 2 | 2 | 3 | 3 | **1** |

*The boldface fonts indicate the measurement data from the plates in the literature.

Table 4 Measurements of the scapula in *Zhanghenglong* and some other hadrosauriforms (mm)*

| Scapula | *Zhanghenglong yangchengensis* | *Probactrosaurus gobiensis* | *Tanius*  *sinensis* | *Olorotitan arharensis* | *Tsintaosaurus spinorhinus* |
| --- | --- | --- | --- | --- | --- |
|  | XMDFEC V0014 | PIN 2232/1-50 | PMU R241 | AEHM 2/495 | IVPP V725 |
| 1. dorsoventral depth of the scapular proximal end | 187.0 | 122.0 | **172.0** | 207.0 | 232.0 |
| 2. distance from the anterior end of the acromial process to the distal margin of the scapular blade | 557.0 | 384.0 | **592.0** | 998.0 | 935.0 |
| 3. dorsoventral height of the scapula neck | 93.0 | 55.0 | **91.0** | 128.0 | 120.0 |
| 4.maximum dorsoventral depth of the scapular blade | 116.0 | 79.0 | **121.0** | 210.0 | 240.0 |
| 5. distance from the anterior margin of the coracoid facet to the anteriormost end of the acromial process | 40.0 | 39.0 | **35.4** | 34.0 | 72.0 |
| ratio |  |  |  |  |  |
| (1) 2:1 | 2.98 | 3.15 | 3.44 | 4.82 | 4.03 |
| (2) 3:1 | 0.50 | 0.45 | 0.53 | 0.62 | 0.52 |
| (3) 4:1 | 0.62 | 0.65 | 0.70 | 1.01 | 1.03 |
| (4) 5:1 | 0.21 | 0.32 | 0.21 | 0.16 | 0.31 |
| angle |  |  |  |  |  |
| angle between the dorsal and ventral margins of the distal portion of the scapular blade | 13º | 19º | **18º** | 2º | 15º |

*The boldface fonts indicate the measurement data from the plates in the literature.
