# Supplementary material for: A New Basal Hadrosauroid Dinosaur (Dinosauria: Ornithopoda) with Transitional Features from the Late Cretaceous of Henan Province, China
Source: PLoS One. 2014 Jun 5;9(6):e98821. doi: 10.1371/journal.pone.0098821 (PMC4047018; doi:10.1371/journal.pone.0098821)
Supplement: Supporting Information S2 — Datasets on the selected measurement attributes for model-based clustering. (DOC) [file pone.0098821.s002.doc]

**Supporting Information S2**

**Datasets on the Selected Measurement Attributes for Model-based Clustering**

All measurement attributes (MA) used here are derived from the characters for the phylogeny of hadrosauroids (see Supporting Information S3), including the ratio between the maximum dorsoventral height and anteroposterior length of the maxilla (character 111; MA1), ratio between the anteroposterior length of the ectopterygoid shelf and that of the maxillary tooth row (character 112; MA2), angle between the ectopterygoid ridge and the ventral margin of the posterior portion of the maxilla (character 113; MA3), ratio between the anteroposterior length of the edentulous region and that of the tooth row of the dentary (character 38; MA4), angle between the medial border of the dentary symphyseal process and the lateral surface of the dentary ramus (character 44; MA5), and ratio between the dorsoventral depth of the scapular neck and the maximum dorsoventral height of the scapular proximal end (character 261; MA6). The one-dimensional dataset of each measurement attribute consists of the direct measurements or ratio calculations on all hadrosauroid species except *Zhanghenglong yangchengensis* and some iguanodontian relatives outside of Hadrosauroidea. Its general distribution could be clearly displayed in the box plot. The Bayesian information criterion (BIC) plot shows the result of model-based cluster analysis (MCA) for each dataset, followed by raw information of the whole analytical process in the operation interface of the package Mclust. In order to clearly address the partition of the database of each MA, the selected basal iguanodontian species were temporarily ascribed to basal hadrosauroids and regarded as the extension of this paraphyletic group.

**Taxonomic Abbreviations:**

**Acg** *Acristavus gagslarsoni*

**Amr** *Amurosaurus riabinini*

**Art** *Aralosaurus tuberiferus*

**Baj** *Bactrosaurus johnsoni*

**Brc** *Brachylophosaurus canadensis*

**Cla** *Claosaurus agilis*

**Coc** *Corythosaurus casuarius*

**Coi** *Corythosaurus intermedius*

**Eda** *Edmontosaurus annectens*

**Edr** *Edmontosaurus regalis*

**Eds** *Edmontosaurus saskatchewanensis*

**Eoc** *Eolambia caroljonesa*

**Eqn** *Equijubus normani*

**Gim** *Gilmoreosaurus mongoliensis*

**Gri** *Gryposaurus incurvimanus*

**Grl** *Gryposaurus latidens*

**Grm** *Gryposaurus monumentensis*

**Grn** *Gryposaurus notabilis*

**Hya** *Hypacrosaurus altispinus*

**Hys** *Hypacrosaurus stebingeri*

**Igb** *Iguanodon bernissartensis*

**Kem** *Kerberosaurus manakini*

**Krn** *Kritosaurus navajovius*

**Lal** *Lambeosaurus lambei*

**Lam** *Lambeosaurus magnicristatus*

**Let** *Levnesovia transoxiana*

**Maa** *Mantellisaurus atherfieldensis*

**Mal** *Magnapaulia laticaudus*

**Mam** *Mandschurosaurus amurensis* (*Charonosaurus jiayinensis*)

**Map** *Maiasaura peeblesorum*

**Nad** *Nanningosaurus dashiensis*

**Ola** *Olorotitan ararhensis*

**Oun** *Ouranosaurus nigeriensis*

**Pac** *Parasaurolophus cyrtocristatus*

**Pai** *Pararhabdodon isonensis*

**Pat** *Parasaurolophus tubicen*

**Paw** *Parasaurolophus walkeri*

**Prb** *Protohadros byrdi*

**Prg** *Probactrosaurus gobiensis*

**Prm** *Prosaurolophus maximus*

**Saa** *Saurolophus angustirostris*

**Sae** *Sahaliyania elunchunorum*

**Sao** *Saurolophus osborni*

**Sek** *Secernosaurus koerneri*

**Shg** *Shantungosaurus giganteus*

**Tas** *Tanius sinensis*

**Tei** *Tethyshadros insularis*

**Tet** *Telmatosaurus transsylvanicus*

**Tss** *Tsintaosaurus spinorhinus*

**Vec** *Velaphrons coahuilensis*

**Wud** *Wulagasaurus dongi*

**Xuy** *Xuwulong yueluni*

**MA1, the ratio between the maximum dorsoventral height and anteroposterior length of the maxilla**


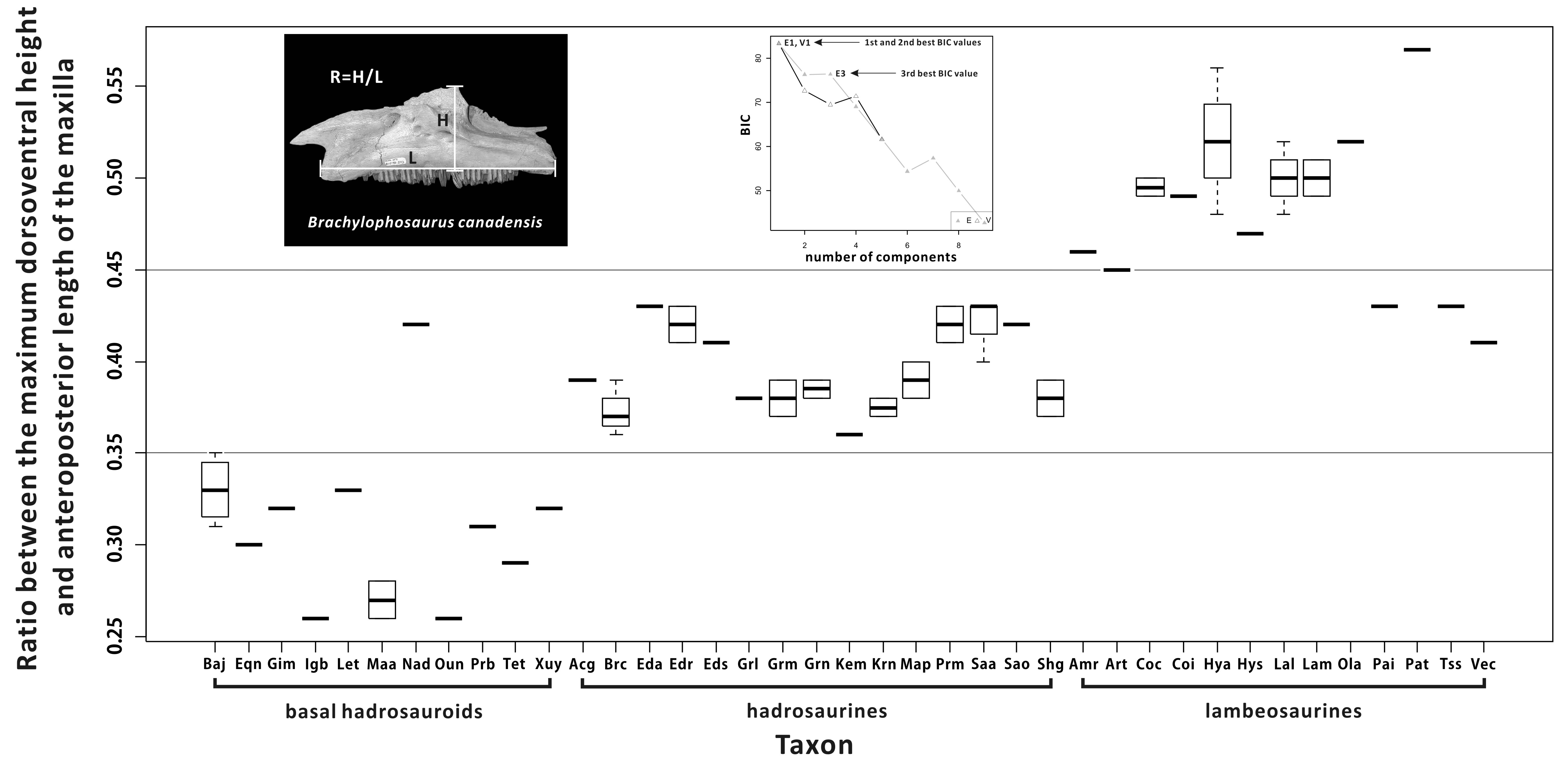


Figure 1. Box plot showing the distribution of the dataset of MA1 on most hadrosauroid taxa and some basal iguanodontian relatives, with the related BIC plot.

**Raw data of model-based clustering on the MA1**

> a <- read.csv("D:/MA1.csv")

> a

X01 X02 X03 X04 X05 X06 X07 X08 X09 X10 X11 X12 X13 X14 X15

0.33 0.30 0.32 0.26 0.33 0.27 0.42 0.26 0.31 0.29 0.32 0.39 0.37 0.43 0.42

X16 X17 X18 X19 X20 X21 X22 X23 X24 X25 X26 X27 X28 X29 X30

0.41 0.38 0.38 0.39 0.36 0.38 0.39 0.42 0.42 0.42 0.38 0.46 0.45 0.50 0.49

X31 X32 X33 X34 X35 X36 X37 X38 X39

0.52 0.47 0.50 0.50 0.52 0.43 0.57 0.43 0.41

> aBIC <- mclustBIC(a)

> aSummary <- summary(aBIC, data = a)

> aSummary

classification table:

1

39

best BIC values:

V,1 E,1 E,3

83.32923 83.32923 76.37013

> aBIC

BIC:

E V

1 83.32923 83.32923

2 76.30104 72.62726

3 76.37013 69.47826

4 69.03813 71.36595

5 61.71035 61.61452

6 54.38394 NA

7 57.34388 NA

8 50.01616 NA

9 42.68920 NA

> aSummary <- summary(aBIC, data = a, G = 3, modelName = "E")

> aSummary

classification table:

1 2 3

10 20 9

BIC value:

E,3

76.37013

For MA1, the first and second best BIC values (83.32923) do not allow the dataset to be partitioned, and represent the variable and equal variances, respectively. In contrast, the dataset could be subdivided into three components by MCA, when the third best BIC value (76.37013) was gained from the equal variance (see text, Fig. 12A, B). In the case of the third best BIC value, the component with a low average value (C01) consists of the values of all measured basal hadrosauroids except *Nanningosaurus dashiensis*, as well as those of the three iguanodontians outside of Hadrosauroidea. It ranges from 0.26 to 0.33. The middle component (C02) represents the closed interval between 0.36 and 0.45, including the values of *Nanningosaurus dashiensis*, all measured hadrosaurines, and four lambeosaurines. The last component (C03) ranges from 0.46 to 0.57, and refers to nine measured lambeosaurines. More specifically, C01 ([0.26, 0.33]) is adequately representative of the dataset which is composed of the values of basal hadrosauroid species and some iguanodontian relatives; C02 plus C03 ([0.36, 0.57]) is generally equivalent to the distribution range of the values of more derived hadrosaurids. The boundary value between the two intervals on MA1 was artificially defined as 0.35. The value of *Zhanghenglong yangchengensis* on MA1 is 0.37. It falls within the interval of hadrosaurids.

**MA2, the ratio between the anteroposterior length of the ectopterygoid shelf and that of the maxillary tooth row**


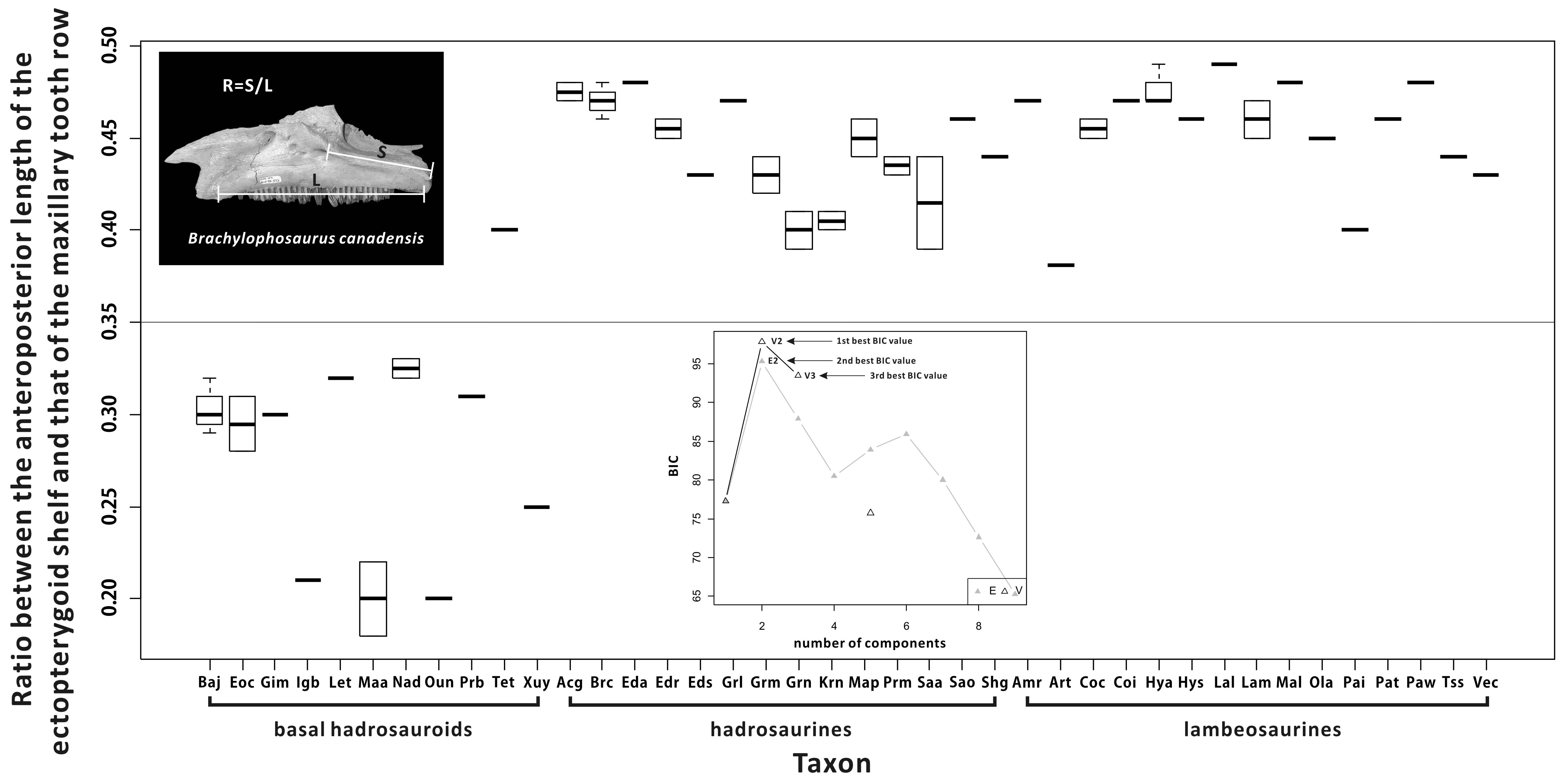


Figure 2. Box plot showing the distribution of the dataset of MA2 on most hadrosauroid taxa and some basal iguanodontian relatives, with the related BIC plot.

**Raw data of model-based clustering on the MA2**

> a <- read.csv("D:/MA2.csv")

> a

X01 X02 X03 X04 X05 X06 X07 X08 X09 X10 X11 X12 X13 X14 X15

0.30 0.30 0.30 0.21 0.32 0.20 0.33 0.20 0.31 0.40 0.25 0.48 0.47 0.48 0.46

X16 X17 X18 X19 X20 X21 X22 X23 X24 X25 X26 X27 X28 X29 X30

0.43 0.47 0.43 0.40 0.41 0.45 0.44 0.42 0.46 0.44 0.47 0.38 0.46 0.47 0.48

X31 X32 X33 X34 X35 X36 X37 X38 X39 X40

0.46 0.49 0.46 0.48 0.45 0.40 0.46 0.48 0.44 0.43

> aBIC <- mclustBIC(a)

> aSummary <- summary(aBIC, data = a)

> aSummary

classification table:

1 2

10 30

best BIC values:

V,2 E,2 V,3

97.83486 95.26882 93.47850

> aBIC

BIC:

E V

1 77.29946 77.29946

2 95.26882 97.83486

3 87.89026 93.47850

4 80.51270 NA

5 83.90857 75.74005

6 85.89652 NA

7 80.00667 NA

8 72.62712 NA

9 65.24834 NA

> aSummary <- summary(aBIC, data = a, G = 2, modelName = "V")

> aSummary

classification table:

1 2

10 30

BIC value:

V,2

97.83486

MCA favored partitioning the dataset on MA2 into two components, when the first best BIC value (97.83486) with respect to the variable variance occurred (see text, Fig. 12C, D). In the case of the first best BIC value, one component (C01) ranges from 0.20 to 0.33, and is composed of the values of all selected basal hadrosauroid species except *Telmatosaurus transsylvanicus* and the three iguanodontians outside of Hadrosauroidea; the other component (C02) reveals the distribution range of the values of all measured hadrosaurids and *Telmatosaurus transsylvanicus*, which varies from 0.38 to 0.49. For MA2, the boundary value between the two intervals was artificially defined as 0.35. The value of *Zhanghenglong yangchengensis* on MA2 is 0.37, which is restricted to the interval of hadrosaurids.

**MA3, the angle between the ectopterygoid ridge and the ventral margin of the posterior portion of the maxilla**


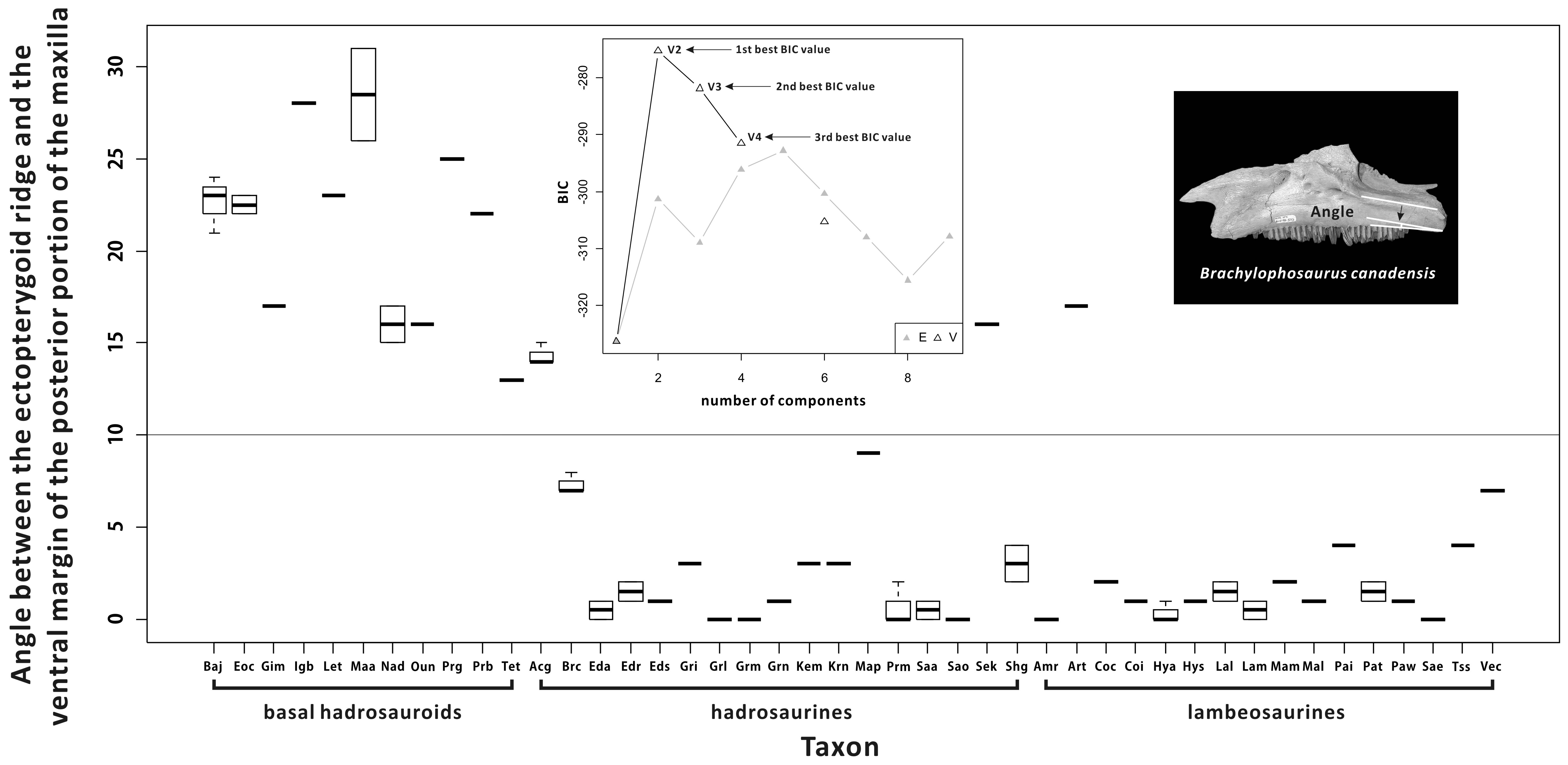


Figure 3. Box plot showing the distribution of the dataset of MA3 on most hadrosauroid taxa and some basal iguanodontian relatives, with the related BIC plot.

**Raw data of model-based clustering on the MA3**

> a <- read.csv("D:/MA3.csv")

> a

X01 X02 X03 X04 X05 X06 X07 X08 X09 X10 X11 X12 X13 X14 X15

23 23 17 28 23 29 16 16 25 22 13 14 7 1 2

X16 X17 X18 X19 X20 X21 X22 X23 X24 X25 X26 X27 X28 X29 X30

1 3 0 0 1 3 3 9 1 1 0 16 3 0 17

X31 X32 X33 X34 X35 X36 X37 X38 X39 X40 X41 X42 X43 X44

2 1 0 1 2 1 2 1 4 2 1 0 4 7

> aBIC <- mclustBIC(a)

> aSummary <- summary(aBIC, data = a)

> aSummary

classification table:

1 2

30 14

best BIC values:

V,2 V,3 V,4

-275.3110 -281.9267 -291.5319

> aBIC

BIC:

E V

1 -326.2804 -326.2804

2 -301.3632 -275.3110

3 -308.9318 -281.9267

4 -296.1316 -291.5319

5 -292.8538 NA

6 -300.4234 -305.3085

7 -307.9915 NA

8 -315.5655 NA

9 -307.8404 NA

> aSummary <- summary(aBIC, data = a, G = 2, modelName = "V")

> aSummary

classification table:

1 2

30 14

BIC value:

V,2

-275.3110

In the case of the first best BIC value (-275.3110) on MA3, MCA has split the dataset into two components on the basis of the variable variance (see text, Fig. 12E, F). The component with a high average value of the data (C01) ranges from 13° to 31°, and contains the values of all selected basal hadrosauroids, the three iguanodontians outside of Hadrosauroidea, *Acristavus gagslarsoni*, *Aralosaurus tuberiferus*, and *Secernosaurus koerneri*. In these hadrosaurids, the ectopterygoid ridge is slightly oblique posteroventrally relative to the ventral border of the posterior portion of the maxilla. The condition differs from the nearly horizontal ectopterygoid ridge seen in other hadrosaurids. The other component (C02) consists of the values of all measured hadrosaurid species except *Acristavus gagslarsoni*, *Aralosaurus tuberiferus*, and *Secernosaurus koerneri*. It constitutes a closed interval between 0° and 9°. C01 ([13°, 31°]) generally represents the dataset composed of the values of basal hadrosauroids and some iguanodontian relatives, although it is confounded by the values from three hadrosaurid species; C02 ([0°, 9°]) reveals the distribution range of the values of most hadrosaurid species. The boundary value between the two intervals on MA3 was artificially defined as 10°. The value of *Zhanghenglong yangchengensis* on MA3 is 16°. It falls within the interval that represents basal hadrosauroids and some iguandontians outside of Hadrosauroidea.

**MA4, the ratio between the anteroposterior length of the edentulous region and that of the tooth row of the dentary**


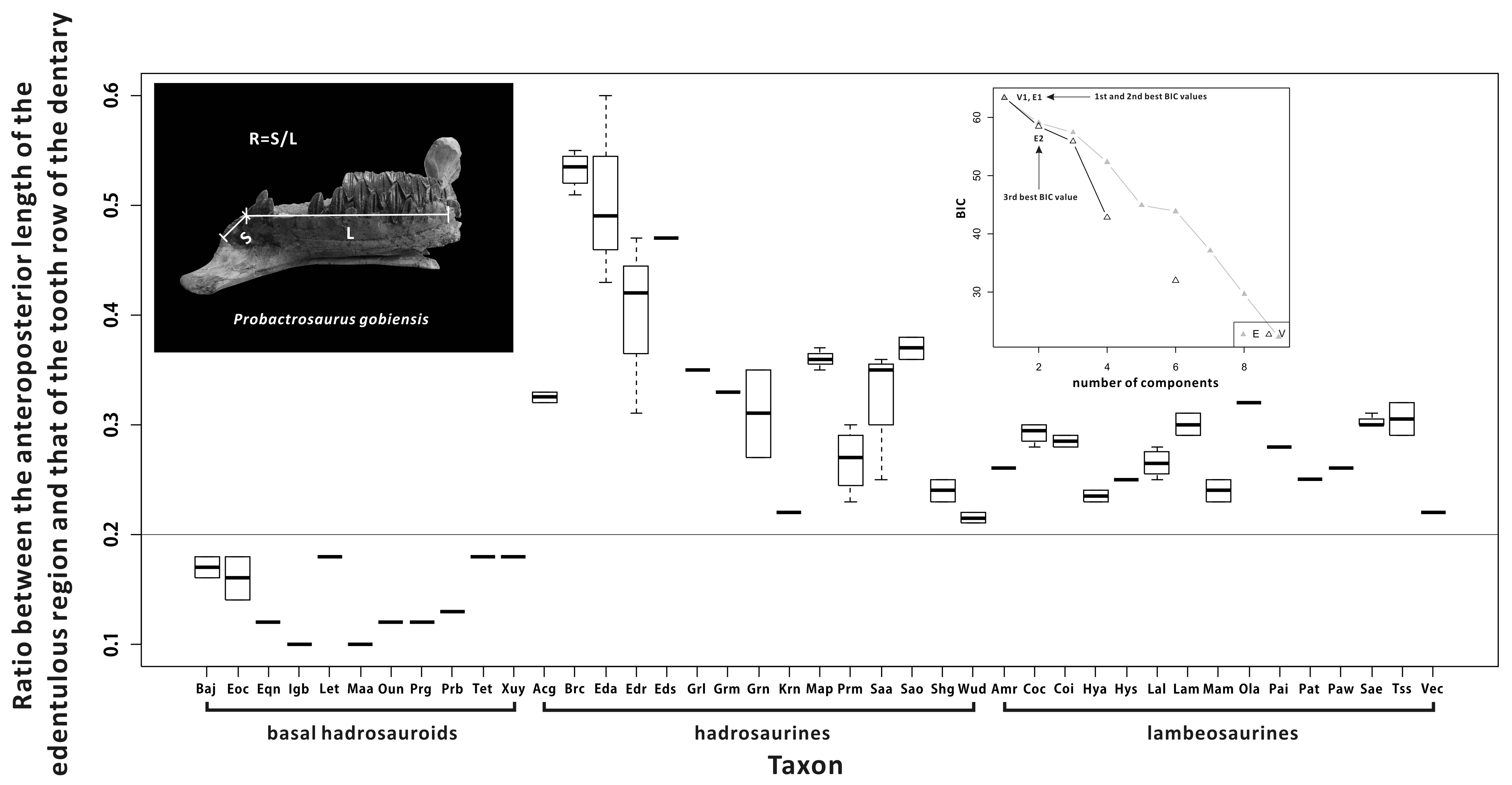


Figure 4. Box plot showing the distribution of the dataset of MA4 on most hadrosauroid taxa and some basal iguanodontian relatives, with the related BIC plot.

**Raw data of model-based clustering on the MA4**

> a <- read.csv("D:/MA4.csv")

> a

X01 X02 X03 X04 X05 X06 X07 X08 X09 X10 X11 X12 X13 X14 X15

0.17 0.16 0.12 0.10 0.18 0.10 0.12 0.12 0.13 0.18 0.18 0.33 0.53 0.51 0.40

X16 X17 X18 X19 X20 X21 X22 X23 X24 X25 X26 X27 X28 X29 X30

0.47 0.35 0.33 0.31 0.22 0.36 0.27 0.32 0.37 0.24 0.22 0.26 0.30 0.29 0.24

X31 X32 X33 X34 X35 X36 X37 X38 X39 X40 X41

0.25 0.27 0.30 0.24 0.32 0.28 0.25 0.26 0.30 0.31 0.22

> aBIC <- mclustBIC(a)

> aSummary <- summary(aBIC, data = a)

> aSummary

classification table:

1

41

best BIC values:

V,1 E,1 E,2

63.38351 63.38351 59.06589

> aBIC

BIC:

E V

1 63.38351 63.38351

2 59.06589 58.46314

3 57.40604 55.87161

4 52.29429 42.77094

5 44.86658 NA

6 43.86039 31.96721

7 37.07645 NA

8 29.63981 NA

9 22.21641 NA

> aSummary <- summary(aBIC, data = a, G = 2, modelName= "E")

> aSummary

classification table:

1 2

11 30

BIC value:

E,2

59.06589

The first and second best BIC values (63.38351) obtained from MCA of the dataset on MA4 do not allow partitioning of the dataset. When the third best BIC value (59.06589) was calculated, the dataset on MA4 could be partitioned into two clusters in the model of the equal variance (see text, Fig. 12G, H). In this specific model, the cluster ranging from 0.10 to 0.18 (C01) contains the values of all measured basal hadrosauroids and the three iguanodontian taxa outside of Hadrosauroidea; the other one (C02) can be regarded as the closed interval between 0.21 and 0.60, which is referable to all measured hadrosaurid species. The boundary value between the two intervals on MA4 was artificially defined as 0.20. The value of *Zhanghenglong yangchengensis* on MA4 is 0.17. It falls within the interval representing basal hadrosauroids and some iguandontians outside of Hadrosauroidea.

**MA5, the angle between the medial border of the dentary symphyseal process and the lateral surface of the dentary ramus**


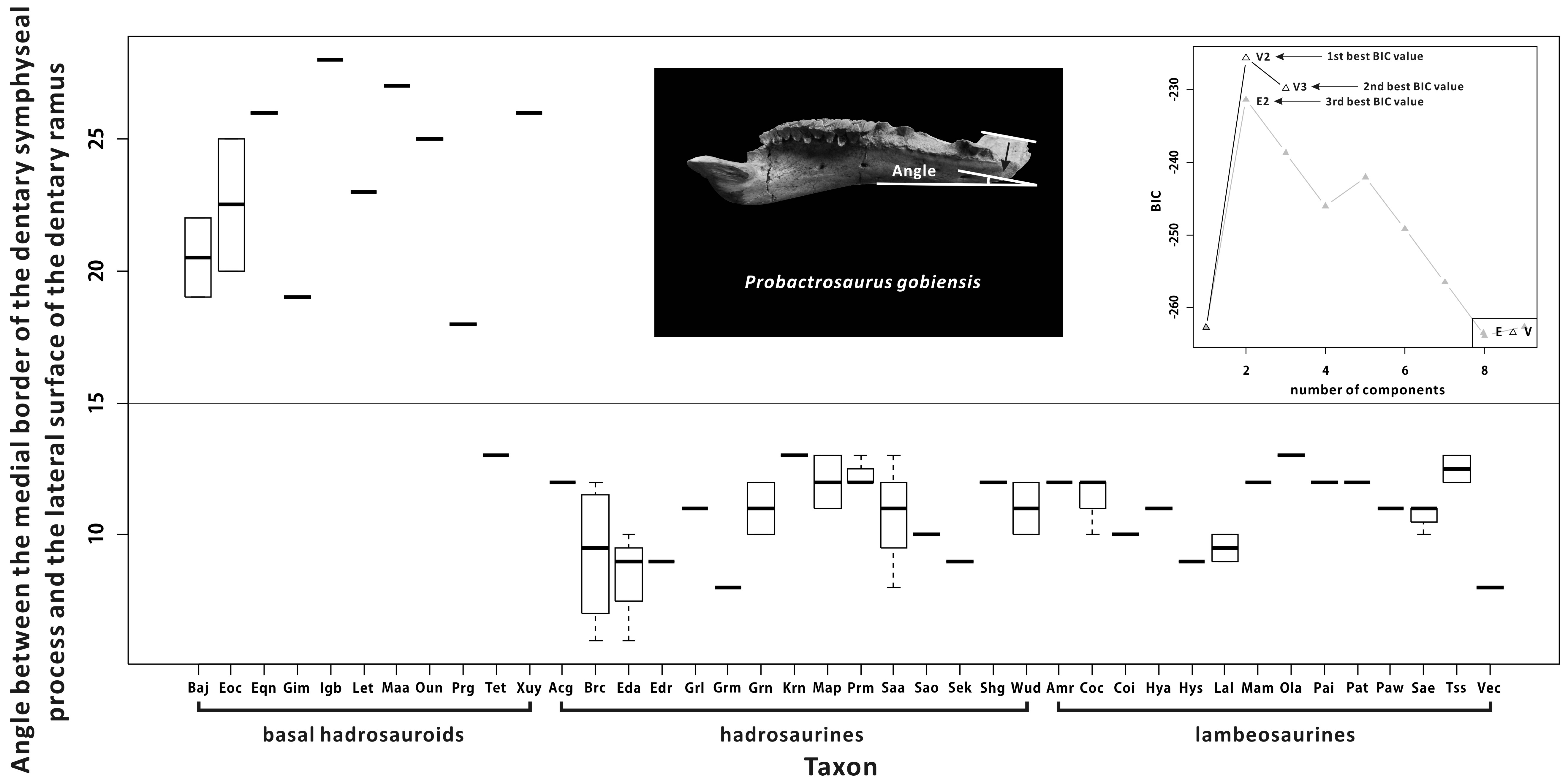


Figure 5. Box plot showing the distribution of the dataset of MA5 on most hadrosauroid taxa and some basal iguanodontian relatives, with the related BIC plot.

**Raw data of model-based clustering on the MA5**

> a <- read.csv("D:/MA5.csv")

> a

X01 X02 X03 X04 X05 X06 X07 X08 X09 X10 X11 X12 X13 X14 X15

21 23 26 19 28 23 27 25 18 13 26 12 9 8 9

X16 X17 X18 X19 X20 X21 X22 X23 X24 X25 X26 X27 X28 X29 X30

11 8 11 13 12 12 11 10 9 12 11 12 11 10 11

X31 X32 X33 X34 X35 X36 X37 X38 X39 X40

9 10 12 13 12 12 11 11 13 8

> aBIC <- mclustBIC(a)

> aSummary <- summary(aBIC, data = a)

> aSummary

classification table:

1 2

30 10

best BIC values:

V,2 V,3 E,2

-225.5681 -229.7913 -231.2989

> aBIC

BIC:

E V

1 -262.8171 -262.8171

2 -231.2989 -225.5681

3 -238.6776 -229.7913

4 -246.0556 NA

5 -242.0501 NA

6 -249.1873 NA

7 -256.5717 NA

8 -263.9444 NA

9 -262.6742 NA

> aSummary <- summary(aBIC, data = a, G = 2, modelName = "V")

> aSummary

classification table:

1 2

30 10

BIC value:

V,2

-225.5681

For MA5, the first best BIC value (-225.5681), which was calculated from the variable variance using MCA, allows the partition of the dataset into two components (see text, Fig. 12I, J). The component with a low average value (C01) is composed of the values of all measured hadrosaurid species and *Telmatosaurus transsylvanicus*. It ranges from 8° to 13°. The values of all selected basal hadrosauroids except *Telmatosaurus transsylvanicus* plus the three iguanodontians outside of Hadrosauroidea constitute the other component (C02), which is interpreted as the closed interval between 18° and 28°. These two subsets (C01 and C02) reveal the quantitative disparity in MA5 between basal hadrosauroids and more derived hadrosaurids. The boundary value between the two intervals on MA5 was artificially defined as 15°. The value of *Zhanghenglong yangchengensis* on MA5 is 13°, which is restricted to the interval of hadrosaurids.

**MA6, the ratio between the dorsoventral depth of the scapular neck and the maximum dorsoventral height of the scapular proximal end**


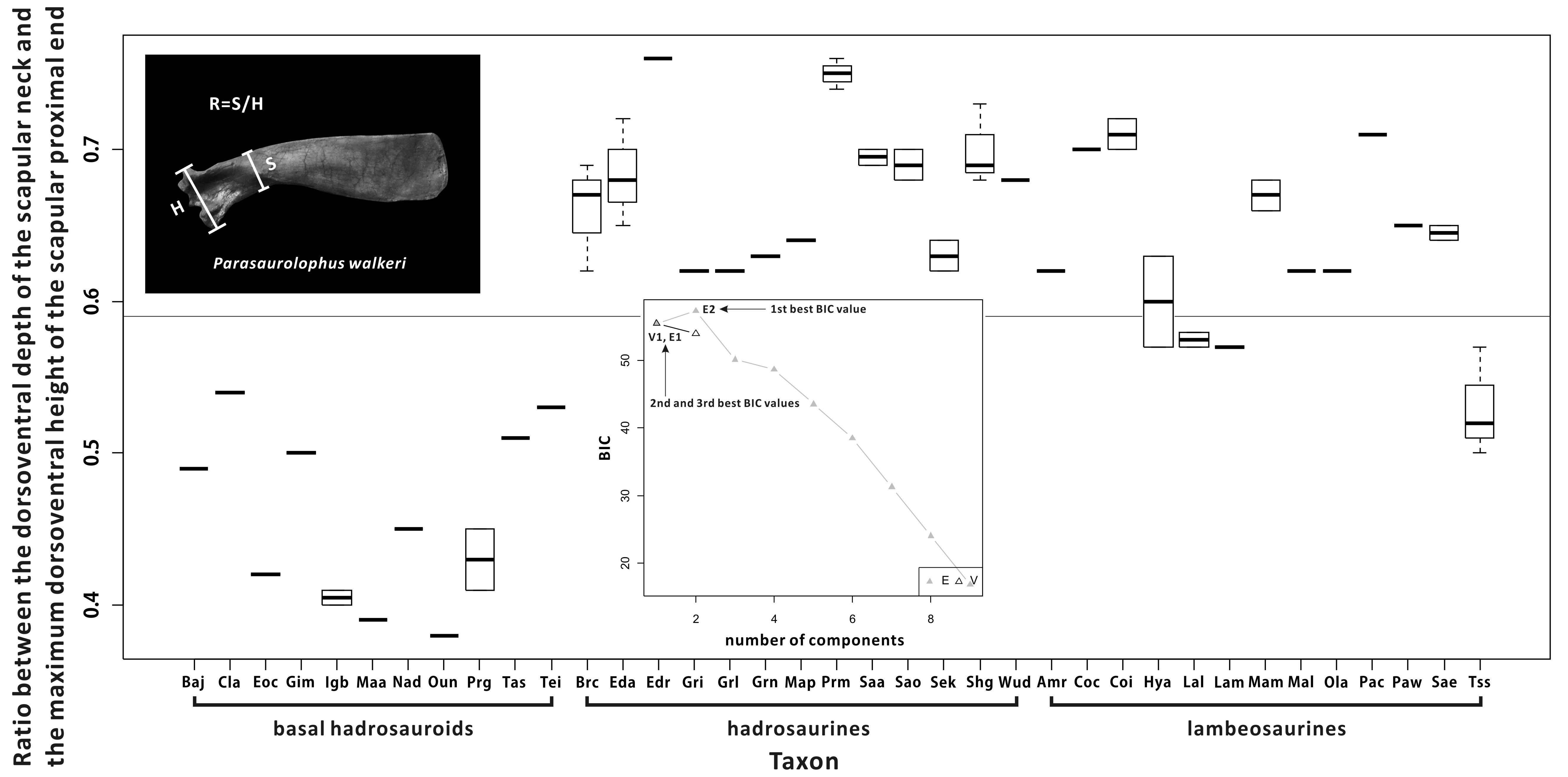


Figure 6. Box plot showing the distribution of the dataset of MA6 on most hadrosauroid taxa and some basal iguanodontian relatives, with the related BIC plot.

**Raw data of model-based clustering on the MA6**

> a <- read.csv("D:/MA6.csv")

> a

X01 X02 X03 X04 X05 X06 X07 X08 X09 X10 X11 X12 X13 X14 X15

0.49 0.54 0.42 0.50 0.41 0.39 0.45 0.38 0.43 0.51 0.53 0.66 0.68 0.76 0.62

X16 X17 X18 X19 X20 X21 X22 X23 X24 X25 X26 X27 X28 X29 X30

0.62 0.63 0.64 0.75 0.70 0.69 0.63 0.70 0.68 0.62 0.70 0.71 0.60 0.58 0.57

X31 X32 X33 X34 X35 X36 X37

0.67 0.62 0.62 0.71 0.65 0.65 0.53

> aBIC <- mclustBIC(a)

> aSummary <- summary(aBIC, data = a)

> aSummary

classification table:

1 2

13 24

best BIC values:

E,2 V,1 E,1

57.29318 55.40313 55.40313

> aBIC

BIC:

E V

1 55.40313 55.40313

2 57.29318 53.91273

3 50.06293 NA

4 48.62499 NA

5 43.49756 NA

6 38.46910 NA

7 31.24711 NA

8 24.02418 NA

9 16.80162 NA

> aSummary <- summary(aBIC, data = a, G = 2, modelName = "E")

> aSummary

classification table:

1 2

13 24

BIC value:

E,2

57.29318

The optimal partition model of the database on MA6 is subdividing the raw data into two clusters with the maximum BIC value (57.29318) calculated from the equal variance using MCA (see text, Fig. 12K, L). The cluster with a low average value (C01) ranges from 0.38 to 0.58. It consists of the values of all selected basal hadrosauroids, the three iguanodontians outside of Hadrosauroidea, the genus *Lambeosaurus*, and *Tsintaosaurus spinorhinus*. The other cluster (C02) could be regarded as the closed interval between 0.60 and 0.76, which is composed of the values of most measured hadrosaurid species. The boundary value between these two clusters was artificially defined as 0.59. Interestingly, the data sample of *Hypacrosaurus altispinus* on MA6 (0.57 and 0.63) spans the preceding two intervals respectively related to basal hadrosauroids and hadrosaurids, although its mean falls within C02. The value of *Zhanghenglong yangchengensis* on MA6 is 0.50, which is restricted within the interval that represents basal hadrosauroids and some iguandontians outside of Hadrosauroidea.
