# Supplementary material for: A New Basal Hadrosauroid Dinosaur (Dinosauria: Ornithopoda) with Transitional Features from the Late Cretaceous of Henan Province, China
Source: PLoS One. 2014 Jun 5;9(6):e98821. doi: 10.1371/journal.pone.0098821 (PMC4047018; doi:10.1371/journal.pone.0098821)
Supplement: Supporting Information S3 — Characters used in the phylogenetic analysis of Hadrosauroidea. (DOC) [file pone.0098821.s003.doc]

**Supporting Information S3**

**Character List**

**Dental Features**

1. Maximum number of dental alveoli (tooth positions) in the dentary dental battery (modified from Horner et al., 2004 character 1): (0) 30 or fewer; (1) 31 to 45; (2) more than 45.
2. Number of dentary teeth per centimeter of the dental battery (modified from Prieto-Marquez and Wagner, 2009 character 2): (0) less than 0.7 tooth/cm for the dental battery; (1) 0.7 or more teeth/cm for the dental battery.
3. Maximum number of teeth per alveolus of the dentary in adults (modified from Horner et al., 2004 character 2): (0) two; (1) three; (2) four to six; (3) seven or more.
4. Maximum number of the functional teeth on the dentary occlusal plane (modified from Prieto-Marquez and Wagner, 2009 character 4): (0) only one functional tooth; (1) one functional tooth on the rostral and caudal portions of the occlusal plane, and up to two near the middle part of the dental battery; (2) two functional tooth on the rostral and caudal portions of the occlusal plane, and up to three near the middle part of the dental battery; (3) three functional teeth throughout most of the occlusal plane (in some cases, with the sporadic presence of four functional teeth), decreasing to two at the rostral and caudal ends of the dental battery.
5. Height/width ratio of the dentary tooth crown in lingual view (corresponding with Prieto-Marquez and Wagner, 2009 character 5): (0) ratio less than 1.95; (1) ratio from 1.95 to 2.70; (2) ratio greater than 2.70 and up to 3.30; (3) ratio greater than 3.30.
6. Maximum number of ridges on the enameled surface of the dentary tooth crown in lingual view (modified from Horner et al., 2004 character 6): (0) five or more in total (a primary and pronounced ridge ranging from the dorsal to the ventral end of the tooth crown, a slightly short secondary ridge and several subsidiary and less developed ridges); (1) three or four in total (a primary and pronounced ridge, a slightly short secondary ridge and one or two subsidiary and less developed ridges); (2) two or three in total (a primary and pronounced ridge and one or two subsidiary and less developed ridges); (3) only one primary ridge.
7. Position of the primary ridge on the enameled surface of the dentary tooth crown (corresponding with Prieto-Marquez and Wagner, 2009 character 7): (0) caudal to the midline of the tooth crown; (1) median for most teeth and maybe caudal to the midline of the tooth crown for some teeth.
8. Shape of the primary ridge of the dentary tooth crown (modified from Godefroit et al., 2000 character 23): (0) straight in all teeth; (1) straight for some teeth and slightly curved for others.
9. Angle between crown and root of dentary teeth (modified from Godefroit et al., 2000 character 24): (0) not more than 110º; (1) more than 110º and at most 130º; (2) more than 130º.
10. General profile of the root of dentary teeth in rostral view (corresponding with Xing et al., 2012 character 10): (0) curved labially; (1) basically straight and slightly inclined lingually.
11. General shape of the marginal denticles on the lingual enameled crowns of dentary teeth (modified from Prieto-Marquez and Wagner, 2009 character 10): (0) bamboo leaf-like denticles along the dorsal half of the crown; (1) slightly big mammilliform denticles along the dorsal half of the crown; (2) relatively small mammilliform denticles along the dorsal half of the crown.
12. Composition of the marginal denticles of dentary teeth (modified from Prieto-Marquez and Wagner, 2009 character 11): (0) each denticle consisting of several small and rounded knobs; (1) each denticle consisting of one rounded knob.
13. Shape of septa that separate the dentary alveoli (corresponding with Prieto-Marquez and Wagner, 2009 character 13): (0) thick and stick-like septa; (1) thin and sheet-like septa.
14. Morphological character of dental alveoli in the dentary (modified from Prieto-Marquez and Wagner, 2009 character 14): (0) short and tongue-like alveoli; (1) long, narrow and upright alveoli which is parallel with each other.
15. Distribution of the enamel of dentary crowns (corresponding with Norman, 2002 character 30): (0) presence of a thin veneer labially, thick lingually; (1) only present lingually.
16. Maximum number of dental alveoli (tooth positions) in the maxillary dental battery (modified from Horner et al., 2004 character 1): (0) 32 or fewer; (1) 33 to 45; (2) more than 45.
17. Number of teeth per centimeter of maxillary dental battery (corresponding with Prieto-Marquez and Wagner, 2009 character 17): (0) less than 1.25 teeth/cm of dental battery; (1) 1.25 or more teeth/cm of dental battery.
18. Comparison between the number of maxillary alveoli (N_m_) and dentary ones (N_d_) in adult specimens (corresponding with Prieto-Marquez and Wagner, 2009 character 18): (0) N_m_ ≤ N_d_; (1) N_m_ > N_d_.
19. Maximum number of functional teeth exposed on the maxillary occlusal plane (modified from Horner et al., 2004 character 3): (0) only one functional tooth; (1) one functional tooth for most of the occlusal plane, and up to two casually near the middle part of the maxilla; (2) two functional teeth throughout the most part of the occlusal plane, decreasing to one at the rostral and caudal ends of the maxilla.
20. Maximum number of ridges on the enameled surface of the maxillary tooth crown in labial view (modified from Horner et al., 2004 character 7): (0) four or more in total (a primary and pronounced ridge ranging from the dorsal to the ventral end of the tooth crown and three or more subsidiary and less developed ridges); (1) one or two in total (a primary and pronounced ridge and at most one subsidiary and less developed ridge);
21. Position of the primary ridge on the enameled surface of the maxillary tooth crown (modified from You et al., 2003 character 36): (0) caudal to the midline of the tooth crown; (1) median for most teeth and maybe caudal to the midline of the tooth crown for the minority of teeth.
22. Shape of the primary ridge of the maxillary tooth crown (modified from Godefroit et al., 2000 character 23): (0) straight for all teeth; (1) straight for some teeth and slightly curved for others.
23. General shape of the marginal denticles on the labial enameled crowns of maxillary teeth (modified from Prieto-Marquez and Wagner, 2009 character 23): (0) saw-toothed denticles along the ventral half of the crown; (1) slightly big mammilliform denticles along the ventral half of the crown; (2) relatively small mammilliform denticles along the ventral half of the crown.

**Mandible**

**Predentary**

1. Ratio between the maximum mediolateral width and maximum rostrocaudal length of the predentary (corresponding with Prieto-Marquez and Wagner, 2009 character 24): (0) less than 1.20; (1) 1.20 to 1.75; (2) more than 1.75.
2. Ratio between the dorsoventral depth of rostral end and the length of lateral process of the predentary (corresponding with Prieto-Marquez and Wagner, 2009 character 25): (0) more than 0.38; (1) 0.38 or less.
3. Shape of the denticles of the predentary rostral margin (modified from Horner et al., 2004 character 13): (0) triangular; (1) trapeziform.
4. Angle between the rostroventral margin of the rostral end and the dorsal margin of the lateral process of the predentary in lateral view (corresponding with Prieto-Marquez and Wagner, 2009 character 26): (0) 75º or greater; (1) 56º to 74º; (2) 40º to 55º; (3) less than 40º.
5. Spacing between two adjacent denticles of the predentary in adult individuals (corresponding with Prieto-Marquez and Wagner, 2009 character 28): (0) the spacing between two adjacent denticles is longer than or equal to the width of each denticle; (1) the spacing between two adjacent denticles is shorter than the width of each denticle but longer than 25% of its width; (2) no obvious spacing between two adjacent denticles, tightly connecting with each other.
6. Number of the denticles on the rostral surface of the half predentary in adults, not including the middle one in the count (modified from Prieto-Marquez and Wagner, 2009 character 29): (0) up to five; (1) six; (2) more than six.
7. Extension of the predentary denticles (corresponding with Prieto-Marquez and Wagner, 2009 character 30): (0) denticles extending to the lateral process of the predentary; (1) denticles only limited to the rostral surface of the predentary.
8. Shape of the joint between the rostral part and the lateral process of the predentary (modified from Horner et al., 2004 character 13): (0) smoothly rounded, forming an bowed predentary in dorsal view; (1) subsquare; (2) almost square, with the presence of a rostrolateral projection.
9. Lateral shelf of the predentary lateral process (corresponding with Xing et al., 2012 character 32): (0) absent; (1) present.
10. Morphological character of lateral shelf on the lateral surface of the predentary lateral process (modified from Prieto-Marquez and Wagner, 2009 character 32): (0) absent, presence of a rostrocaudally short and shallow depression at the caudal end of the lateral process; (1) presence of a short and less developed lateral shelf along the laterocaudal margin of the lateral process; (2) presence of a short, well incised and mediolaterally broad lateral shelf along the rostrolateral margin of the lateral process; (3) presence of a long and well incised lateral shelf throughout the lateral margin of the lateral process, forming a mediolaterally broad plane; (4) presence of a long, well incised and mediolaterally narrow lateral shelf throughout the lateral margin of the lateral process.
11. Strong posterior extension of the caudolateral corner of the predentary lateral process (corresponding with Prieto-Marquez and Wagner, 2009 character 33): (0) absent, the lingual margin of the lateral process is slightly longer or nearly equal to the labial margin of the lateral process; (1) present, the labial margin of the lateral process is much longer than the lingual margin of the lateral process.
12. Development of the dorsal median process of the predentary (modified from Prieto-Marquez and Wagner, 2009 character 34): (0) presence of a faint dorsal median process that is slightly protrudent caudodorsally; (1) presence of a well developed dorsal median process without a prominent ridge; (2) presence of a well developed dorsal median process with a prominent ridge on its lingual side, obviously extending caudally from the caudal margin of the predentary rostral region.
13. Comparison between the rostrocaudal length of the ventral median process (L) and the dorsoventral depth (D) of the rostral end of the predentary (modified from Prieto-Marquez and Wagner, 2009 character 35): (0) L > D; (1) L ≤ D.
14. Rostrocaudal length of the split of the bilobate ventral median process relative to the undivided portion in ventral view (modified from Prieto-Marquez and Wagner, 2009 character 36): (0) short split and relatively long undivided portion; (1) long split and relatively short undivided portion.

**Dentary**

1. Ratio between the length of the edentulous slope anterior to the dentary dental battery (not including the rostralmost portion which articulates with the predentary) and the horizontal distance between the first tooth position and the caudal end of the coronoid process (corresponding with Prieto-Marquez and Wagner, 2009 character 37): (0) less than 0.20; (1) 0.20 to 0.31; (2) greater than 0.31 and up to 0.45; (3) greater than 0.45.
2. Angle between the edentulous slope of dentary rostral portion and the level (corresponding with Prieto-Marquez and Wagner, 2009 character 38): (0) less than 150º; (1) 150º or greater.
3. Angle between the slope of the dentary rostral region which articulates with the predentary and the horizontal (modified from Prieto-Marquez and Wagner, 2009 character 39): (0) greater than 130º; (1) 115º to 130º; (2) less than 115º.
4. Degree of downward deflection of the rostral part of the dentary which articulates with the predentary (modified from Prieto-Marquez and Wagner, 2009 character 40): (0) faintly curved ventrally, with the angle which is less than 17º; (1) moderately curved ventrally, with the angle between 17º to 25º; (2) markedly curved ventrally, with the angle which is greater than 25º.
5. Ratio between horizontal distance from the caudal margin of the coronoid process to the caudalmost end of the deflected ventral margin of the dentary and horizontal distance from the caudal margin of the coronoid process to the rostralmost alveolus (corresponding with Prieto-Marquez and Wagner, 2009 character 41): (0) greater than 0.78; (1) 0.66 to 0.78; (2) less than 0.66.
6. Ratio between the maximum mediolateral width of the dentary symphysial region and the minimum breadth of the dentary caudal to the dentary symphysial region in dorsal view (modified from Prieto-Marquez and Wagner, 2009 character 42): (0) up to 1.65; (1) greater than 1.65 and up to 2.60; (2) more than 2.60.
7. Angle between the medial surface of dentary symphysis and the lateral surface of the rostral region of the dentary in ventral view (modified from Prieto-Marquez and Wagner, 2009 character 43): (0) greater than 15º; (1) up to 15º.
8. General profile of the dorsal margin of the dentary rostral region which articulates with the predentary in medial or lateral view (corresponding with Prieto-Marquez and Wagner, 2009 character 45): (0) a smooth and gradually descending dorsal margin in the rostrodorsal region of the dentary; (1) a relatively steep dorsal margin, forming a prominent depression in the dentary symphysial region.
9. Degree of the lingual curvature of the dentary symphysial region in rostral view (corresponding with Prieto-Marquez and Wagner, 2009 character 44): (0) markedly curved lingually, forming a nearly 90º lateroventral profile in rostral view; (1) gently curved lingually, forming a sloping, wide arcuate lateroventral profile in rostral view.
10. General profile of the dentary ventral margin under the coronoid process in lateral view (modified from Prieto-Marquez and Wagner, 2009 character 46): (0) a slightly bowed ventral margin ventral to the coronoid process; (1) a well developed bowed ventral margin ventral to the coronoid process.
11. Coronoid process (modified from Godefroit et al., 2008 character 39): (0) basically vertical to the dorsal margin of the dentary or inclined caudally; (1) slightly inclined rostrally, with an angle between 70º and 85º; (2) obviously inclined rostrally, with an angle less than 70º.
12. Shape of the apex of the coronoid process in adult individuals (modified from Horner et al., 2004 character 17): (0) only slightly expanded rostrally, with less developed rostral and caudal margins; (1) markedly expanded rostrocaudally, with well developed rostral and caudal margins; (2) markedly expanded rostrocaudally, with well developed rostral and caudal margins, bearing a more pronounced caudal margin.
13. Medial wall of the triangular depression on the caudal side of the coronoid process in caudal view (**new character**): (0) not extending to the base of the dorsal apex, whose width is about one-quarter of the maximum mediolateral width of the apex; (1) extending to the base of the dorsal apex, whose width is about one-third of the maximum mediolateral width of the apex.
14. A sharp projection on the caudodorsal surface of the coronoid process of the dentary (corresponding with Prieto-Marquez and Wagner, 2009 character 49): (0) absent; (1) present.
15. Development of the ridge on the medial surface of the coronoid process of the dentary (corresponding with Prieto-Marquez and Wagner, 2009 character 50): (0) presence of a faint, projecting medially ridge on the medial surface of the coronoid process; (1) presence of a well developed and expanded caudally ridge, forming the mediodorsal boundary of a depressed facet which contacts the rostrodorsal process of the surangular.
16. Angle between the lateral surface of the dentary portion rostral to the coronoid process and that of the dentary portion rostroventral to the coronoid process in dorsal or ventral view (corresponding with Prieto-Marquez and Wagner, 2009 character 51): (0) the lateral surface of the dentary portion ventral to the coronoid process is only slightly expanded laterally, with an angle greater than 165º; (1) the lateral surface of the dentary portion ventral to the coronoid process is remarkably extended laterally, with an angle up to 165º.
17. Orientation of the longitudinal axis of the dentary occlusal plane relative to the lateral margin of the dentary in dorsal view (corresponding with Prieto-Marquez and Wagner, 2009 character 52): (0) presence of a relatively inclined longitudinal axis, forming an angle of about 20º with the lateral margin of the dentary; (1) presence of a longitudinal axis running parallel with the lateral margin of the dentary.
18. Lingual curvature of the longitudinal axis of the dentary occlusal plane in dorsal view (corresponding with Prieto-Marquez and Wagner, 2009 character 53): (0) present; (1) absent.
19. Position of the coronoid process relative to the dentary dental battery (modified from Horner et al., 2004 character 10): (0) the caudal margin of the coronoid process is caudal to the caudal end of the dental battery; (1) the caudal margin of the coronoid process is overlapped with the caudal end of the dental battery; (2) the caudal margin of the coronoid process is rostral to the caudal end of the dental battery.
20. Degree of separation between the dentary dental battery and the coronoid process (corresponding with Prieto-Marquez and Wagner, 2009 character 55): (0) the coronoid process is not obviously separate from the dentary dental battery; (1) the coronoid process is obviously separate from the dentary dental battery, with the presence of a depressed facet separating the base of coronoid process from the dental battery.
21. Lateral profile of the dentary in adult individuals (corresponding with Xing et al., 2012 character 57): (0) greatly elongated and dorsoventrally narrow, forming a relatively slender dentary in lateral view; (1) moderately long and dorsoventrally deep, forming a robust dentary in lateral view.

**Surangular**

1. Surangular foramen (corresponding with Norman, 2002 character 27): (0) present; (1) absent.
2. Surangular accessory foramen on the rostrolateral surface of the surangular (corresponding with Kobayashi and Azuma, 1999 character 15): (0) present; (1) absent.
3. Articulating facet of the surangular rostrodorsal process which contacts the coronoid process of the dentary (corresponding with Xing et al., 2012 character 60): (0) the rostral surface of the surangular rostrodorsal process, with the presence of the articulation with the caudal surface of the coronoid process; (1) the lateral surface of the surangular rostrodorsal process, with the presence of the articulation with the labial surface of the caudal depression of the coronoid process, owing to the insertion of the surangular rostrodorsal process into the caudal triangular depression of the coronoid process.
4. Position of the apex of the surangular rostrodorsal process (corresponding with Xing et al., 2012 character 61): (0) located at two-thirds of the height of the coronoid process; (1) located at one-third of the height of the coronoid process.
5. Participation of the surangular in the ventral side of the mandibular caudal end (modified from Prieto-Marquez and Wagner, 2009 character 59): (0) absent, surangular only participating in the lateral side of the mandibular caudal end; (1) present, surangular facing more laterally than ventrally; (2) present, surangular facing more ventrally than laterally.
6. Strong upward curvature of the caudal process of the surangular (corresponding with Prieto-Marquez and Wagner, 2009 character 60): (0) absent; (1) present.
7. Lateral profile of the surangular caudoventral corner ventral to the retroarticular process (**new character**): (0) broadly arcuate; (1) a nearly 90º angle.
8. General shape of the retroarticular process of the surangular (**new character**): (0) relatively robust in lateral view, forming a round, blunt caudodorsal extremity; (1) subtriangular in lateral view, forming a caudodorsally tapering extremity.
9. Angle between the ventromedial margin of the rostral part of the surangular and that of the surangular caudal process (modified from Prieto-Marquez and Wagner, 2009 character 61): (0) 150º or greater; (1) less than 150º.

**Angular**

1. Position of the angular of the mandible (corresponding with Weishampel et al., 1993 character 26): (0) the angular exposed in lateral view, with a ventral and slightly lateral position; (1) the angular not exposed in lateral view, with a medial position.

**Coronoid**

1. Coronoid bone (corresponding with Wagner, 2001 character 28): (0) present; (1) absent.

**Prearticular**

1. Prearticular bone (corresponding with Prieto-Marquez and Wagner, 2009 character 64): (0) present; (1) absent.

**Splenial**

1. Position of the mandibular splenial relative to the dentary dental battery in medial view (corresponding with Xing et al., 2012 character 68): (0) the splenial ventral to the dental battery; (1) the splenial caudal to dental battery.

**Other Viscerocranial Bones**

**Premaxilla**

1. Shape of the premaxillary oral margin in dorsal view (corresponding with Horner et al., 2004 character 22): (0) horseshoe-shaped, forming a continuous semicircle that curves smoothly to postoral constricted region; (1) broadly arcuate in the oral margin and abruptly constricted behind the oral margin.
2. Degree of transverse expansion of the premaxilla in the oral region (ratio between the maximum premaxillary width and the minimum width of the premaxilla at posterior contractive region) (modified from Prieto-Marquez and Wagner, 2009 character 65): (0) faintly expanded, with the ratio less than 1.70; (1) moderately expanded, with the ratio between 1.70 and 2.00; (2) markedly expanded, with the ratio greater than 2.00.
3. Degree of downward deflection of the rostral end of the premaxilla relative to the occlusal plane of the dentition (corresponding with Norman, 2002 character 2): (0) slightly deflected ventrally from the occlusal plane; (1) strongly deflected ventrally from the occlusal plane, almost covering the predentary in lateral view.
4. Morphological character of the rostral portion of the premaxillary oral margin (modified from Prieto-Marquez and Wagner, 2009 character 67): (0) presence of a relatively narrow, dorsoventrally thick and slightly deflected ventrally oral margin rostral to the circumnarial fossa; (1) presence of a moderately expanded breadthwise, dorsoventrally thin and strongly deflected ventrally oral margin, forming a smooth and gentle slope from the circumnarial fossa to the premaxillary oral margin; (2) presence of a moderately expanded transversely, dorsoventrally thin and strongly deflected ventrally oral margin which is slightly recurved and rostral to the premaxillary accessory narial fossa; (3) presence of a moderately expanded breadthwise, dorsoventrally thick and strongly deflected ventrally oral margin which is lip-shaped and markedly recurved.
5. Shape of the rostrolateral corner of the premaxillary oral margin (corresponding with Prieto-Marquez and Wagner, 2009 character 68): (0) rounded; (1) triangular.
6. Premaxillary foramen in the rostral region of external naris that opens onto the palatine (corresponding with Horner et al., 2004 character 23): (0) absent; (1) present.
7. Premaxillary accessory foramen entering into the premaxillary accessory narial fossa, located rostral to the premaxillary foramen (corresponding with Horner et al., 2004 character 24): (0) absent; (1) present, sharing a common chamber with the premaxillary foramen.
8. Premaxillary accessory narial fossa located rostral to the circumnarial fossa (corresponding with Horner et al., 2004 character 26): (0) absent; (1) present.
9. Premaxillary oral margin with a “double layer” morphology consisting of a dorsal denticle-bearing layer and a ventral layer which is offset slightly from the oral margin and separated from the denticular layer by a deep sulcus bearing vascular foramina (corresponding with Horner et al., 2004 character 25): (0) absent; (1) present.
10. At least one oval foramen on the anteromedial surface of the premaxillary lip (corresponding with Prieto-Marquez, 2010 character 287): (0) absent; (1) present.
11. Premaxillary additional accessory fossa located lateral to the premaxillary accessory narial fossa and rostral to the circumnarial fossa (corresponding with Prieto-Marquez and Wagner, 2009 character 73): (0) absent; (1) present.
12. The structure of the external naris rostral to the orbit (modified from Horner et al., 2004 character 27): (0) the external naris consists of the premaxilla and the nasal, and premaxillary caudodorsal process does not meet premaxillary caudoventral process posterior to the external naris; (1) the external naris only consists of the premaxilla, and premaxillary caudodorsal and caudoventral processes elongate and meet posterior to the external naris, forming the caudal margin of the external naris and excluding the nasal.
13. Degree of the development of premaxillary caudodorsal process in adult individuals (modified from Prieto-Marquez and Wagner, 2009 character 74): (0) relatively short, forming the dorsal margin of the external naris with the nasal; (1) moderately elongate caudodorsally, forming the dorsal margin of the external naris sorely but not participating in the hollow supracranial crest; (2) markedly elongate caudodorsally and long, forming the rostral part of the hollow supracranial crest; (3) markedly elongate caudodorsally and very long, forming the rostral and caudodorsal portions of the hollow supracranial crest; (4) markedly elongate caudodorsally and very long, forming the rostral and most caudal portions of the hollow supracranial crest.
14. Degree of the development of premaxillary caudoventral process in adult individuals (modified from Prieto-Marquez and Wagner, 2009 character 76): (0) relatively short, the caudal end of premaxillary caudoventral process is located rostrodorsal to the lacrimal; (1) moderately elongate caudodorsally, the caudal end of premaxillary caudoventral process is located rostrodorsal to the prefrontal; (2) remarkably elongate caudodorsally and long, the caudal end of premaxillary caudoventral process is located dorsal or caudodorsal to the prefrontal, forming the rostroventral part of the hollow supracranial crest.
15. Premaxillary caudoventral process articulating with the nasal (corresponding with Xing et al., 2012 character 82): (0) present; (1) absent.
16. Vertical groove on the caudoventral process of the premaxilla, located rostral to the dorsal process of the maxilla (corresponding with Evans and Reisz, 2007 character 5): (0) absent; (1) present.
17. Morphological character of the caudal region of the premaxillary caudoventral process in adult individuals (modified from Prieto-Marquez and Wagner, 2009 character 77): (0) mediolaterally compressed, arrowhead-shaped and rostral to the orbit; (1) arrowhead-shaped, rostrocaudally expanded and dorsal to the orbit; (2) triangular, dorsoventrally expanded and dorsal to the orbit.
18. The bone caudal to the premaxillary caudoventral process (corresponding with Xing et al., 2012 character 85): (0) lacrimal; (1) nasal.
19. Premaxillary caudodorsal process with an accessory rostroventral flange that overlaps the lateral surface of the nasal in the rostral region of the crest (corresponding with Evans and Reisz, 2007 character 18): (0) absent; (1) present.
20. Shape of the external naris (modified from Evans and Reisz, 2007 character 4): (0) basically spindle-shaped or elliptical; (1) triangular, the caudodorsal and caudoventral margins of the external naris gradually constricted caudodorsally in the caudal region; (2) relatively short and teardrop-shaped, the caudodorsal and caudoventral margins of the external naris abruptly constricted caudodorsally in the caudal region.
21. Ratio between the rostrocaudal length and the maximum mediolateral width of the external naris of derived lambeosaurine dinosaurs in dorsolateral view (modified from Prieto-Marquez and Wagner, 2009 character 81): (0) less than 4; (1) 4 or greater.
22. Deflection angle of the dorsal margin of the premaxillary caudodorsal process rostral to the lacrimal in the adult individuals of lambeosaurine dinosaurs (modified from Prieto-Marquez and Wagner, 2009 character 194): (0) greater than 170º; (1) 100º to 170º; (2) less than 100º.
23. A well developed dorsolateral flange which is sheet-shaped in the middle of mediolaterally compressed premaxillary caudoventral process in non-lambeosaurine iguanodontoidean dinosaurs (corresponding with Prieto-Marquez and Wagner, 2009 character 82): (0) absent; (1) present.

**Maxilla**

1. Rostrodorsal process of the maxilla (corresponding with Horner et al., 2004 character 42): (0) presence of the maxillary rostrodorsal process extending the region medial to the premaxillary caudoventral process and forming a part of ventral margin of the external naris; (1) absence of the maxillary rostrodorsal process, the rostrodorsal portion of the maxilla forming a sloping shelf which underlies the premaxilla.
2. Lateral exposure of the rostrodorsal process of the maxilla (corresponding with Gates and Sampson, 2007 character 45): (0) absent, the rostrodorsal process cannot be observed through external naris in lateral view; (1) present, the rostrodorsal process can be seen through external naris in lateral view.
3. Anterior extension of the rostrodorsal process of the maxilla (**new character**): (0) the rostrodorsal process is relatively truncated and low, and does not exceed the anteriormost end of the rostroventral process; (1) the rostrodorsal process is elongated and elevated, and exceeds the anteriormost end of the rostroventral process.
4. Strongly downward curvature of the rostroventral process of the maxilla (corresponding with Prieto-Marquez and Wagner, 2009 character 94): (0) present; (1) absent.
5. Angle between the dorsal margin of the maxillary rostroventral process and the ventral margin of the maxillary rostral portion where the dental battery begins to appear (modified from Prieto-Marquez and Wagner, 2009 character 95): (0) less than 25º; (1) 25º to 40º; (2) greater than 40º.
6. General shape of the maxillary lateral surface rostroventral to the maxillary facet articulating with the jugal (modified from Prieto-Marquez and Wagner, 2009 character 96): (0) trapeziform or subrectangular; (1) triangular and rostrocaudally compressed.
7. Ratio between the distance from the apex of the maxillary lateral surface rostroventral to jugal articular surface to the rostral end of the maxilla and the rostrocaudal length of the maxilla (corresponding with Prieto-Marquez and Wagner, 2009 character 98): (0) more than 0.57; (1) 0.47 to 0.57; (2) 0.35 to 0.46; (3) less than 0.35.
8. Location of the apex of the adult maxilla in lateral view (corresponding with Horner et al., 2004 character 47): (0) markedly caudal to the midline of the maxilla; (1) at or rostral to the midline of the maxilla.
9. Shape of the dorsal process (dorsal ramus) of the maxilla in lateral view (modified from Horner et al., 2004 character 48): (0) triangular and the width is greater than the height, bearing a slightly round apex; (1) triangular and the height is greater than the width, bearing a peaked and caudally inclined apex.
10. Morphological character of the jugal articular surface in the dorsolateral region of the maxilla (modified from Prieto-Marquez and Wagner, 2009 character 100): (0) presence of a finger-shaped and laterally offset jugal articular surface located at two-thirds of the length of the maxilla; (1) presence of a subtriangular and laterally inclined jugal articular surface located rostrodorsal to the ectopterygoid shelf; (2) presence of an irregular diamond-shaped jugal articular surface at the midline of the maxilla, bearing a dorsolaterally facing ventral half which is slightly inclined caudally and rostral to the ectopterygoid shelf in lateral view; (3) presence of a regular diamond-shaped jugal articular surface at the midline of the maxilla, bearing a dorsolaterally facing ventral half rostral to the ectopterygoid shelf; (4) presence of a subrectangular and dorsally elevated jugal articular surface rostrodorsal to the ectopterygoid shelf.
11. Arrangement of the maxillary foramina rostroventral to the jugal articulating facet (corresponding with Prieto-Marquez and Wagner, 2009 character 101): (0) the maxillary foramina are arranged rostrocaudally and scattered on the lateral surface of the maxilla; (1) the maxillary foramina are arranged in a row and oriented rostrodorsally.
12. Number of the maxillary foramina rostroventral to the jugal articulating facet excluding the large rostral foramen (modified from Prieto-Marquez and Wagner, 2009 character 102): (0) seven or more; (1) at most six.
13. One or two large maxillary foramina immediately adjacent to the ventral extremity of the jugal articulation of the maxilla (**new character**): (0) absent; (1) present.
14. Location of large rostral maxillary foramen (modified from Evans and Reisz, 2007 character 22): (0) located on the rostral half of the maxillary rostrolateral surface and exposed in lateral view; (1) located on the dorsal half of the maxillary rostrolateral surface and exposed in lateral view; (2) located on the maxillary rostrodorsal surface along the maxilla-premaxilla articular surface and not exposed in lateral view.
15. Lateral exposure of the antorbital fenestra (corresponding with You et al., 2003 character 4): (0) present; (1) absent.
16. Lateral exposure of the maxilla-lacrimal contact (corresponding with Evans and Reisz, 2007 character 23): (0) present in lateral view; (1) absent in lateral view and laterally covered by the jugal-premaxilla contact.
17. Ratio between the vertical distance from the apex of the maxillary dorsal process to the maxillary ventral margin and the maximum length of the maxillary ventral margin (modified from Weishampel et al., 1993 character 20): (0) up to 0.35; (1) greater than 0.35 and up to 0.45; (2) greater than 0.45.
18. Ratio between the length of the ectopterygoid shelf and the rostrocaudal length of the maxillary dental battery (corresponding with Prieto-Marquez and Wagner, 2009 character 105): (0) up to 0.25; (1) greater than 0.25 and at most 0.35; (2) more than 0.35.
19. Degree of the inclination of the ectopterygoid shelf (angle between the lateral margin of the ectopterygoid shelf and the rostrocaudal axis of the maxillary dental battery in lateral view) (modified from Prieto-Marquez and Wagner, 2009 character 106): (0) presence of a steeply inclined caudoventrally ectopterygoid shelf with an angle greater than 20º; (1) presence of a markedly inclined caudoventrally ectopterygoid shelf with an angle from 10º to 20º; (2) presence of a slightly inclined caudoventrally ectopterygoid shelf with an angle greater than 5º and less than 10º; (3) presence of a almost horizontal ectopterygoid shelf with an angle up to 5º.
20. Degree of the development of the lateral ridge of the ectopterygoid shelf (modified from Godefroit et al., 2000 character 14): (0) presence of a less developed and dorsoventrally thin ridge on the lateral surface of the maxillary caudal portion; (1) presence of a faint ridge which is dorsoventrally thin rostrally and dorsoventrally thick caudally along the lateral margin of the maxillary caudal segment; (2) presence a pronounced and dorsoventrally thick ridge on the lateral surface of the maxillary caudal portion.
21. Location of the middle part of the special foramina (neurovascular alveolar foramina) on the medial side of the maxilla relative to half dorsoventral height of the maxilla excluding the height of the dorsal process (corresponding with Prieto-Marquez and Wagner, 2009 character 108): (0) ventral to or at the half height of the maxilla; (1) dorsal to the half height of the maxilla.

**Jugal**

1. Morphological character of the caudodorsal margin of the jugal rostral process (modified from Weishampel et al., 1993 character 15): (0) dorsoventrally narrow and slightly curved ; (1) dorsoventrally deep and markedly recurved.
2. Rostral apex of the rostral process of the jugal (modified from Prieto-Marquez and Wagner, 2009 character 111): (0) presence of a very long, wedge-shaped and sharp rostral apex located at the middle point of dorsoventral depth of the rostral process; (1) presence of a long, wedge-shaped and sharp rostral apex located near the dorsal half of the rostral process; (2) presence of a short, mammilla-shaped and blunt rostral apex near the dorsal half of the rostral process; (3) presence an extremely short, less developed and blunt rostral apex near the dorsal half of the rostral process; (4) absence of the rostral apex.
3. General shape of the ventral margin of the jugal rostral process (corresponding with Prieto-Marquez and Wagner, 2009 character 113): (0) bowed or curved; (1) inverted triangular, with the width approximately equal to the height; (2) inverted triangular and often recurved caudally, with the height greater than the width.
4. Position of palatine articulating facet on the medial surface of the jugal rostral process (corresponding with Xing et al., 2012 character 113): (0) dorsal to the maxillary articular surface; (1) caudal to the maxillary articular surface and dorsal to the maxillary process of the jugal.
5. Degree of the inclination of palatine articular surface of the jugal (angle between the rostrocaudal axis of the jugal and the long axis of palatine articular surface) (modified from Prieto-Marquez and Wagner, 2009 character 116): (0) extremely inclined rostrally and almost horizontal, with an angle of approximately 180º; (1) obviously inclined rostrally, with an angle between 110º and 130º; (2) slightly inclined rostrally and almost vertical, with an angle less than 110º.
6. Location of the ventral apex of the jugal rostral process relative to the caudal margin of the lacrimal process of the jugal(the longitudinal axis of the rostral process represents horizontal) (corresponding with Prieto-Marquez and Wagner, 2009 character 114): (0) located caudoventral to the caudal margin of the lacrimal process; (1) located ventral to the caudal margin of the lacrimal process.
7. Morphological character of concave ventral margin of the jugal (modified from Norman, 2002 character 16): (0) relatively shallow and wide; (1) relatively deep and narrow.
8. Ratio between maximum dorsoventral depth of the jugal caudoventral portion ventral to the infratemporal fenestra and minimum vertical distance of the caudal contractive portion of the jugal (modified from Prieto-Marquez and Wagner, 2009 character 118): (0) up to 1.35; (1) greater than 1.35 and up to 1.55; (2) more than 1.55.
9. General profile of the caudal process of the jugal in lateral view (corresponding with Prieto-Marquez and Wagner, 2009 character 119): (0) auricular in shape and relatively slender, with a nearly straight caudal margin; (1) auricular in shape and relatively wide rostrocaudally, with a convex caudal margin and a short blunt apex along the dorsal margin of the caudal process; (2) auricular in shape and relatively wide rostrocaudally, with a convex caudal margin and a tall pointed apex along the dorsal margin of the caudal process; (3) regular fan-shaped, with a nearly straight or slightly convex caudal margin.
10. Morphological character of the concavity of the caudoventral margin of the jugal (modified from Weishampel et al., 1993 character 18): (0) relatively short and shallow; (1) relatively long and deep.
11. Ratio between minimum vertical distance of the caudal contractive portion of the jugal ventral to the infratemporal fenestra and the distance from the lowest point of the orbital rim to the lowest point of the rim of the infratemporal fenestra (modified from Weishampel et al., 1993 character 13): (0) up to 0.5; (1) greater than 0.5 and less than 0.6; (2) 0.6 to 0.8; (3) greater than 0.8.
12. Width of the orbital margin of the jugal relative to that of the infratemporal margin of the bone (corresponding with Prieto-Marquez and Wagner, 2009 character 123): (0) wider orbital margin; (1) the orbital margin and infratemporal margin are almost equally wide; (2) wider infratemporal margin.
13. Ectopterygoid articulating facet on the medial side of the jugal (corresponding with Godefroit et al., 2001 character 12): (0) present; (1) absent.

**Quadratojugal**

1. General shape of the quadratojugal in lateral view (corresponding with Xing et al., 2012 character 123): (0) L-shaped; (1) trapeziform.

**Quadrate**

1. Degree of the curvature of the quadrate in lateral view (angle between the long axis of the dorsal portion of the quadrate and the one of the ventral portion of the quadrate) (modified from Wagner, 2001 character 35): (0) slightly curved caudally, with an angle more than 150º; (1) strongly curved caudally, with an angle up to 150º.
2. Position of the quadratojugal notch relative to the dorsoventral height of the quadrate (corresponding with Prieto-Marquez and Wagner, 2009 character 125): (0) the midpoint of the quadratojugal notch is located near the half dorsoventral height of the quadrate; (1) the midpoint of the quadratojugal notch is located ventral to the half dorsoventral height of the quadrate.
3. Angle between the dorsal margin of the quadratojugal notch and the long axis of the quadrate (modified from Prieto-Marquez and Wagner, 2009 character 126): (0) greater than 40º; (1) up to 40º.
4. General shape of the quadratojugal notch of the quadrate (modified from Weishampel and Horner, 1986): (0) semicircular and relatively deep rostrocaudally, with the ventral margin of the notch which is slightly recurved and oriented rostrally; (1) wide arcuate and relatively shallow rostrocaudally, with the ventral margin of the notch which is smooth and oriented rostroventrally; (2) wide arcuate and relatively shallow rostrocaudally, with the ventral margin of the notch which is slightly recurved and oriented rostrally.
5. Squamosal buttress (dorsocaudal protuberance) on the caudal surface of the dorsal end of the quadrate (modified from Weishampel and Horner, 1986): (0) presence of a well developed protuberant buttress; (1) absence of the buttress or presence of a less developed faint buttress.
6. Ratio between the rostrocaudal length of the lateral condyle and the mediolateral width of the ventral end of the quadrate (modified from Weishampel et al., 1993 character 22): (0) up to 0.75; (1) more than 0.75.
7. Degree of the elevation of the medial condyle of the quadrate in rostral view (modified from Prieto-Marquez and Wagner, 2009 character 129): (0) the medial condyle is slightly elevated upwards relative to the lateral condyle; (1) the medial condyle is remarkably elevated upwards relative to the lateral condyle.
8. Lateral exposure of the quadratojugal notch of the quadrate (modified from Prieto-Marquez and Wagner, 2009 character 198): (0) present; (1) absent.

**Neurocranial Bones**

**Nasal**

1. Hollow supracranial crest (corresponding with Godefroit et al., 2008 character 6): (0) absent; (1) present.
2. Solid supracranial crest (modified from Horner et al., 2004 character 38): (0) absent; (1) present but not laterally excavated by caudal expansion of the circumnarial fossa; (2) present but laterally excavated by caudal expansion of the circumnarial fossa.
3. Supracranial crest in iguanodontoidean dinosaurs (modified from Horner et al., 2004 character 40): (0) absent; (1) present, composed of nasals; (2) present, composed of frontals and nasals; (3) present, composed of nasals and premaxillae.
4. Participation of the nasal in the hollow crest (modified from Godefroit et al., 2008 character 19): (0) absent, with the presence of the nasal rostral to the orbit forming a part of the margin of the external naris; (1) present, with the presence of the nasal rostral to the orbit forming a part of the margin of the external naris and bearing a less developed crest-like structure which is hollow; (2) present, with the presence of the nasal dorsal to the orbit forming the entire hollow crest which is well developed; (3) present, with the presence of the well developed nasal dorsal to the orbit participating in half part of the hollow crest and forming the caudal half of the hollow crest; (4) present, with the presence of the well developed nasal dorsal to the orbit participating in a small part of the hollow crest and excluded from the caudodorsal region of the hollow crest.
5. Position of the nasal cavity (common median chamber inside the crest) in the adult skull (modified from Horner et al., 2004 character 33): (0) restricted to an almost horizontal area rostromedial to the orbit; (1) restricted to a vertical tube-shaped area rostrodorsal to the orbit; (2) restricted to an ellipsoid-shaped area dorsal to the orbit.
6. General shape of the caudal part of the nasal in non-lambeosaurine iguanodontoidean dinosaurs (corresponding with Xing et al., 2012 character 137): (0) presence of a relatively flat caudal part without any promontory or crest; (1) presence of a low dome-shaped protuberance on the dorsal surface of the caudal part; (2) presence of a pronounced promontory in the caudodorsal region of the nasal; (3) presence a paddle-like caudal portion extending to the region dorsal to the braincase; (4) presence of a long and stick-shaped caudal portion which bears a middle ridge and is triangular in transverse cross section, extending to the region dorsal to the occiput; (5) presence of a recurved and abruptly elevated caudodorsally caudal portion forming a sharp protuberance with the frontal; (6) presence of a gradually elevated caudodorsally and laterally depressed caudal part dorsal to the rostral part of the frontal.
7. The nasal inserting under the premaxillary caudoventral process (corresponding with Xing et al., 2012 character 138): (0) absent; (1) present.
8. Morphological feature of the rostral end of the nasal articulating with premaxillary caudodorsal process in lateral view (modified from Horner et al., 2004 character 34): (0) presence of a slender and wedge-shaped rostral end, gradually decreasing rostrally in width and depth; (1) presence of a relatively thick and subrectangular rostral end, abruptly and markedly decreasing rostrally in depth near the rostralmost end; (2) presence of a long and finger-shaped rostral end without any prominent change in depth; (3) presence of a particular rostral end bearing a vertical tube-shaped structure with a relatively thick base articulating with premaxillary caudodorsal process; (4) presence of a relatively slender rostral end fitting along the ventral margin of premaxillary caudodorsal process; (5) presence of a thick and sheet-shaped rostral end with bilobate rostral margin meeting premaxilla in a complex W-shaped interfingering suture.
9. Morphological feature of caudoventral portion of the nasal articulating with premaxillary caudoventral process in non-lambeosaurine iguanodontoidean dinosaurs (corresponding with Prieto-Marquez and Wagner, 2009 character 86): (0) the nasal forms a subrectangular flange, exposed dorsal to the premaxillary caudoventral process; (1) the nasal forms a large long hook-like rostroventral process, exposed dorsal to the premaxillary caudoventral process; (2) the nasal forms a greatly shortened and dorsoventrally narrow hook-like rostroventral process, exposed dorsal to the premaxillary caudoventral process.
10. Caudalmost apex of the external naris (corresponding with Godefroit et al., 2008 character 16): (0) consisting of nasal and premaxilla; (1) consisting entirely of nasal; (2) consisting entirely of premaxilla.
11. Participation of the rostral end of the nasal in the rostral margin of the external naris (modified from Prieto-Marquez and Wagner, 2009 character 87): (0) absent, the rostral end of the nasal is located at one-third of the length of the external naris; (1) absent, the rostral end of the nasal participating in hollow supracranial crest is far away from the rostral margin of the external naris; (2) present, the rostral end of the nasal reaches the rostral margin of the external naris.
12. Caudal end of the nasals forming a pair of processes along the frontonasal suture of the skull (modified from Gates and Sampson, 2007 character 65): (0) absent; (1) present, the caudal end of the nasals forms a pair of divergent processes lying on top of the frontals; (2) present, the caudal end of the nasals forms a pair of conjunctive processes inserted into the frontals.
13. A pronounced promontory in the caudodorsal region of the nasal anterior to the orbit (modified from Prieto-Marquez and Wagner, 2009 character 91): (0) absent; (1) present, the summit of the promontory is located dorsal to the caudal margin of the external naris; (2) present, the summit of the promontory is located caudodorsal to the caudal margin of the external naris.
14. Lateral profile of the dorsal margin of the rostrum dorsal to the external naris (modified from Prieto-Marquez and Wagner, 2009 character 176): (0) slightly convex and arcuate; (1) highly convex and arcuate; (2) basically straight.
15. Angle between the long axis of the external naris and the maxillary dental battery (modified from Prieto-Marquez and Wagner, 2009 character 177): (0) less than 30º; (1) 30º to 40º; (2) more than 40º.
16. Lateral exposure of the circumnarial fossa (nasal vestibule) (corresponding with Xing et al., 2012 character 147): (0) present, with the presence of the circumnarial fossa surrounding the external naris; (1) absent, with the presence of the circumnarial fossa enclosed within the caudodorsal and caudoventral premaxillary processes and internalized.
17. The roof of the nasal passage in the rostral region of the skull (modified from Horner et al., 2004 character 27): (0) formed by the nasal; (1) formed by premaxillary caudodorsal process owing to the nasal passages completely enclosed by tubular premaxilla.
18. Nasal passage divided into two parts (corresponding with Xing et al., 2012 character 149): (0) absent, presence of only one nasal passage connecting with cavum nasi proprium; (1) present, presence of two nasal passages (left and right ones) connecting with lateral diverticulum and common medial chamber.
19. Caudal extension of the nasal passage (modified from Evans and Reisz, 2007 character 9): (0) absent, the nasal passage restricted to the region rostral to the orbit; (1) present, the nasal passage extending to the region dorsal to the orbit; (2) present, the nasal passage extremely extending to the region caudodorsal to the occipital.
20. The narial foramen which is external opening of the nasal (corresponding with Xing et al., 2012 character 151): (0) presence of the narial foramen which is equivalent to the external naris when the nasal is located rostral to the orbit; (1) absence of the narial foramen when the nasal is located above the orbit and becomes a part of hollow supracranial crest.
21. General shape of the narial foramen in lateral view (corresponding with Prieto-Marquez and Wagner, 2009 character 179): (0) broad dorsoventrally and elliptic; (1) narrow dorsoventrally and subelliptic; (2) extremely narrow dorsoventrally and slit-shaped.
22. Premaxilla-nasal fontanelle surrounded by the nasal and the premaxilla on the lateral surface of the supracranial crest (modified from Norman, 2002 character 5): (0) absent; (1) present, the nasal passage is incompletely closed on the lateral surface of the crest because of the premaxilla-nasal fontanelle persisting into the late ontogenetic stage; (2) present, the nasal passage is completely closed on the lateral surface of the crest because of the premaxilla-nasal fontanelle disappearing in the adult stage.
23. Ratio between the length of the external naris and the length of the lateroventral margin of the rostrum of the skull in non-lambeosaurine iguanodontoidean dinosaurs (corresponding with Prieto-Marquez and Wagner, 2009 character 181): (0) up to 0.40; (1) greater than 0.40 but less than 0.60; (2) 0.60 to 0.65; (3) greater than 0.65.
24. Caudal margin of the circumnarial fossa caudodorsal to the narial foramen (corresponding with Horner et al., 2004 character 31): (0) absent, the circumnarial fossa does not reach the caudal margin of the narial foramen; (1) present, the circumnarial fossa reaches the caudal margin of the narial foramen.
25. Morphological character of the caudal region of the circumnarial fossa along the caudal margin of the external naris (corresponding with Horner et al., 2004 character 32): (0) absent; (1) present, slightly incised into nasal and premaxilla, often poorly demarcated; (2) present, deeply incised into nasal and premaxilla, well demarcated, and usually invaginated.
26. Strongly invaginated, excavated circumnarial fossa enclosing the entire external naris (**new character**): (0) absent; (1) present, the circumnarial fossa is dorsoventrally tall along its entire length, with a prominent, arched caudodorsal margin; (2) present, the circumnarial fossa is unusually low dorsoventrally in its middle and caudal regions, with a thick, right-angled caudodorsal margin.
27. Strong elevation or specialized development of the nasal (corresponding with Prieto-Marquez and Wagner, 2009 character 190): (0) absent; (1) present.
28. Morphological feature and evolutionary stage of the hollow supracranial crest in lambeosaurine dinosaurs (modified from Horner et al., 2004 character 36): (0) the nasal anterior to the orbit forms the caudodorsal margin of the external naris, bearing a less developed crest-like structure which is hollow and protuberant; (1) the nasal, which extends dorsally owing to the caudodorsal expansion of the premaxilla, forms exclusively a vertical tube-shaped hollow crest dorsal to the orbit; (2) the premaxilla extends caudodorsally to facilitate the backward growth and development of the nasal, forming a long curved tubular hollow crest dorsal to the occiput with the nasal; (3) the premaxilla extends caudodorsally to facilitate the backward growth and development of the nasal, forming a thin plate-like or cockscomb-shaped hollow crest dorsal to the occiput with the nasal.
29. Position of the caudalmost end of the nasal of adult lambeosaurine dinosaurs in lateral view (corresponding with Prieto-Marquez and Wagner, 2009 character 195): (0) rostrodorsal to the orbit; (1) dorsal to the caudal margin of the orbit; (2) dorsal to the occiput.
30. S-loop in the nasal passage (nasal vestibule) completely enclosed by premaxilla rostral to the orbit (corresponding with Evans and Reisz, 2007 character 8): (0) absent; (1) present.
31. Lateral diverticulum connecting with common medial chamber of nasal cavity (corresponding with Xing et al., 2012 character 161): (0) absence of the lateral diverticulum; (1) presence of the lateral diverticulum as a part of the cavum nasi proprium (the lateral diverticulum and common median chamber are together homologous with the cavum nasi proprium).
32. Position of the lateral diverticulum relative to the common median chamber (corresponding with Weishampel, 1981): (0) caudodorsal to the common median chamber; (1) lateral to the common median chamber; (2) rostral to the common median chamber.
33. Connection between the nasal passage and the lateral diverticulum inside the hollow crest (corresponding with Weishampel, 1981 and Evans, 2006): (0) absent, the nasal passage completely enclosed by the premaxilla connects directly with the common median chamber without the participation of the lateral diverticulum; (1) present, the nasal passage connects with the lateral diverticulum which communicates with the common median chamber in succession.

**Lacrimal**

1. General shape of the lacrimal of the adult individuals in lateral view (modified from Prieto-Marquez and Wagner, 2009 character 109): (0) subquadrangular or trapeziform, with a shortened rostroventral corner; (1) trapeziform and rostrocaudally elongate, with a long rostral process; (2) triangular and rostrocaudally elongate, with a straight and dorsoventrally thick rostral process; (3) triangular and rostrocaudally elongate, with a curved and relatively slender rostral process.

**Prefrontal**

1. Participation of the prefrontal in the lateral border of the hollow supracranial crest (corresponding with Godefroit et al., 2008 character 22): (0) absent; (1) present.
2. A well-developed and oriented caudodorsally ridge on the dorsal surface of the prefrontal as the medial wall of the rostral platform supporting the base of the hollow crest (modified from Prieto-Marquez and Wagner, 2009 character 130): (0) absence of the prefrontal ridge; (1) presence of the prefrontal ridge not extending caudally to the prefrontofrontal suture; (2) presence of the prefrontal ridge extending caudally over the dorsal surface of the frontal.
3. General shape of the rostrodorsal margin of the prefrontal along the orbital rim in lateral view (modified from Horner et al., 2004 character 50): (0) arcuate or smoothly curved; (1) nearly right-angled.
4. Morphological character of the laterally exposed prefrontal rostral portion articulating with the lacrimal (corresponding with Prieto-Marquez and Wagner, 2009 character 132): (0) mediolaterally wide and relatively robust in lateral view; (1) mediolaterally narrow and relatively slender in lateral view.
5. Eversion of the rostrodorsal orbital margin of the prefrontal (corresponding with Horner et al., 2004 character 49): (0) absent, the prefrontal lies flush with surrounding elements; (1) present, the prefrontal flares dorsolaterally to form a thin, everted, wing-shaped margin around rostrodorsal orbital rim.
6. Degree of the lateral exposure of the prefrontal-nasal contact (modified from Wagner, 2001 character 37): (0) the prefrontal-nasal contact is completely exposed in lateral view; (1) only the caudal region of the prefrontal-nasal contact is visible in lateral view, owing to the covering of the premaxilla along the laterodorsal margin of the prefrontal.

**Palpebral**

1. Palpebral (supraorbital) bone (corresponding with Norman, 2002 character 13): (0) present; (1) absent or fused to the orbital margin.

**Postorbital**

1. Dorsal promontorium on the dorsal surface of the postorbital rostral process in adult specimens (corresponding with Godefroit et al., 2004 character 17): (0) absent; (1) present.
2. A prominent depression on the dorsal surface of the postorbital above the ventral process of the element (corresponding with Xing et al., 2012 character 173): (0) absent, the dorsal surface of the postorbital is flat or slightly concave; (1) present, the dorsal surface of the postorbital is deeply depressed.
3. Degree of the curvature of the caudodorsal orbital margin of the postorbital (modified from Xing et al., 2012 character 174): (0) presence of a slightly concave rostroventrally or almost straight orbital margin obliquely bridging the rostral and ventral processes of the postorbital; (1) presence of a strongly concave rostroventrally orbital margin that is often semicircular or deeply arcuate.
4. Morphological character of the triangular ventral process of the postorbital (modified from Prieto-Marquez and Wagner, 2009 character 138): (0) presence of a relatively robust ventral process in lateral view, strongly expanding rostroventrally along the rostral margin of its dorsal half; (1) presence of a slender ventral process that is gradually tapering ventrally; (2) presence of a rostrocaudally broad ventral process in lateral profile, strongly expanded along its rostral and caudal margins.
5. Strongly bulgy laterally main body of the postorbital bearing a deep inner cavity located anterior to the medial ridge of the ventral process of the postorbital (modified from Prieto-Marquez and Wagner, 2009 character 138): (0) absent; (1) present.
6. Morphological character of the caudal process (squamosal process) of the postorbital (corresponding with Evans and Reisz, 2007 character 36): (0) presence of a relatively long caudal process forming a broad dorsal margin of the infratemporal fenestra; (1) presence of a relatively short and dorsoventrally deep caudal process forming a constricted dorsal margin of the infratemporal fenestra.
7. Bifurcation of the caudal process of the postorbital (corresponding with Evans and Reisz, 2007 character 35): (0) absent; (1) present.
8. General length of the ventral process of the postorbital relative to maximum rostrocaudal width of the orbit (modified from Prieto-Marquez and Wagner, 2009 character 139): (0) presence of a relatively short ventral process which is shorter than the rostrocaudal width of the orbit; (1) presence of a relatively long vental process which is approximately equal to the rostrocaudal width of the orbit.
9. Position of the caudal end of the postorbital caudal process relative to the quadrate cotylus of the squamosal (corresponding with Prieto-Marquez and Wagner, 2009 character 141): (0) located rostral to the quadrate cotylus; (1) located above the rostral half of the quadrate cotylus; (2) located dorsal to the caudal end of the quadrate cotylus.

**Squamosal**

1. Dorsoventral expansion of the main body of the squamosal in lateral view (modified from Horner et al., 2004 character 64): (0) slightly expanded dorsoventrally above the quadrate cotylus; (1) markedly expanded dorsoventrally and elevated above the quadrate cotylus.
2. Degree of the elongation of the precotyloid process of the squamosal (ratio between the length of the precotyloid process and the width of the quadrate cotylus of the squamosal) (corresponding with Prieto-Marquez and Wagner, 2009 character 142): (0) presence of a relatively short precotyloid process, with the ratio less than 1.00; (1) presence of a moderately long precotyloid process, with the ratio from 1.00 to 1.25; (2) presence of a very long precotyloid process, with the ratio greater than 1.25.
3. Rostromedially oriented strong curvature of the medial ramus of the squamosal (corresponding with Prieto-Marquez and Wagner, 2009 character 145): (0) present; (1) absent.
4. Separation of the squamosals along the occipital margin of the skull roof in adult specimens (corresponding with Horner et al., 2004 character 63): (0) present, the squamosals are widely separated by the parietal; (1) present, the squamosals approach the midline of the skull and are separated by a narrow band of the parietal; (2) absent, the medial rami of the squamosals closely contact with each other.

**Frontal**

1. Rostroventrally sloping frontal platform (nasal articular surface of the frontal) for supporting the supracranial crest (corresponding with Xing et al., 2012 character 184): (0) absent; (1) present.
2. Deeply excavated rostral platform composed of the prefrontal and the frontal (corresponding with Godefroit et al., 2008 character 8): (0) absent; (1) present, the rostral platform is inclined rostroventrally and occupies the rostral part of the frontal in adults; (2) present, the rostral platform markedly extends caudodosally and overhangs the parietal in adults.
3. Angle between the dorsal surface of the frontal rostral part and the level (corresponding with Xing et al., 2012 character 186): (0) up to 10º; (1) greater than 10º and less than 25º; (2) 25º to 35º; (3) more than 35º.
4. Median cleft on the nasal articular surface of the frontal (corresponding with Evans and Reisz, 2007 character 40): (0) absent; (1) present.
5. Frontal doming on the dorsal surface of the braincase in subadult or adult individuals (modified from Horner et al., 2004 character 58): (0) absent; (1) present.
6. Participation of the frontal in the dorsal margin of the orbit (corresponding with Horner et al., 2004 character 57): (0) present, the frontal forms part of the orbital margin; (1) absent, the frontal is excluded by prefrontal-postorbital contact.
7. Ratio between the rostrocaudal length of the interfrontal suture of the frontal ectocranial surface and the maximum mediolateral width of the frontal ectocranial surface in dorsal view (modified from Godefroit et al., 2008 character 7): (0) presence of a relatively long ectocranial surface, with the ratio greater than 0.8; (1) presence of a relatively short ectocranial surface because of the extension of the nasal articular surface of the frontal, with the ratio from 0.4 to 0.8; (2) presence of an extremely short ectocranial surface by reason of the thickening of the nasal articular surface of the frontal which obviously extends caudodosally and overhangs the parietal, with the ratio less than 0.4.
8. Fontanelle of the skull roof in juvenile or subadult individuals (corresponding with Xing et al., 2012 character 191): (0) presence of the nasal-frontal fontanelle surrounded by the nasal and the frontal; (1) presence of the premaxilla-nasal fontanelle enclosed by the nasal and the premaxilla; (2) absence of the fontanelle of the skull roof.
9. Nasal-frontal fontanelle on the dorsal surface of the braincase (modified from Prieto-Marquez and Wagner, 2009 character 147): (0) present, the nasal-frontal fontanelle is still well developed and large in subadults or young adults; (1) absent, the nasal-frontal fontanelle is strongly contractive and entirely closed in subadults or young adults; (2) absent, the nasal-frontal fontanelle cannot be identified owing to the development of the supracranial crest.
10. Morphological character of annular ridge on the ventral side of the frontal that defines the rostral extent of the cerebral fossa (corresponding with Evans and Reisz, 2007 character 43): (0) relatively wide and shallow; (1) relatively narrow and sharp.

**Parietal**

1. Ratio between the rostrocaudal length of the parietal along the sagittal plane and the width of the parietal at half the length in subadult and adult individuals (modified from Godefroit et al., 2008 character 2): (0) less than 2.2; (1) 2.2 or greater.
2. Downward curvature of the parietal dorsal surface in lateral view (modified from Horner et al., 2004 character 69): (0) absent, the parietal dorsal surface is nearly straight relative to the skull roof; (1) absent, the parietal dorsal surface is gradually ascending caudally relative to the skull roof; (2) present, the parietal dorsal surface is slightly downwarped along its length; (3) present, the parietal dorsal surface is strongly downwarped relative to the skull roof.
3. Morphological character of the median anterior process of the parietal along the frontoparietal suture (modified from Prieto-Marquez and Wagner, 2009 character 157): (0) arcuate or subtriangular, rostrocaudally short and mediolaterally wide; (1) finger-shaped or subtriangular, rostrocaudally long and mediolaterally narrow.
4. Sagittal crest on the dorsal surface of the parietal (modified from Horner et al., 2004 character 70): (0) present, the sagittal crest only extends along the rostral half of the parietal, and splits into two secondary crests on the dorsal surfaces of paired parietal caudolateral processes; (1) present, the sagittal crest extends along the entire length of the parietal, and its rostral half is relatively wide mediolaterally, gradually narrowing and sharpening caudally; (2) present, the sagittal crest extends along the entire length of the parietal, and keeps narrow and sharp all the while; (3) present, the sagittal crest only extends along the caudal half of the parietal, and the rostral half of the parietal dorsal surface is relatively flat.
5. Articulation of the parietal caudolateral process for the medial ramus of squamosal (**new character**): (0) the parietal caudolateral process meets the medial ramus of squamosal caudomedially; (1) the parietal caudolateral process contacts the medial ramus of squamosal dorsolaterally.

**Supraoccipital**

1. Orientation of the caudal surface of the supraoccipital (corresponding with Horner et al., 2004 character 65): (0) nearly vertical and oriented caudally; (1) markedly inclined rostrally and oriented caudodorsally.
2. Lateroventral portion of the supraoccipital deeply inserted into the exoccipital, with two short flanges along the supraoccipital-exoccipital suture in caudal view (corresponding with Horner et al., 2004 character 66): (0) absent; (1) present.
3. Degree of the caudal extension of the supraoccipital-exoccipital shelf relative to the foramen magnum in ventral view (modified from Godefroit et al., 2008 character 26): (0) slightly elongate caudally; (1) moderately extended caudally; (2) extremely extended caudally.

**Exoccipital**

1. Orientation of the distal end of the paroccipital process (corresponding with Horner et al., 2004 character 62): (0) rostroventrally directed; (1) ventrally directed.

**Basioccipital**

1. Participation of the basioccipital in the ventral margin of the foramen magnum (corresponding with Weishampel et al., 1993 character 24): (0) absent, the paired exoccipital condyles completely exclude the basioccipital from the ventral margin of the foramen magnum; (1) present, the exoccipital condyles are well separated and allow the basioccipital to form the ventral margin of the foramen magnum.
2. Shallow groove of the occipital condyle along the exoccipital-basioccipital junction in caudal view (modified from Prieto-Marquez and Wagner, 2009 character 160): (0) absent; (1) present.
3. Degree of the constriction of the basioccipital portion between the occipital condyle and the spheno-occipital tubercles (corresponding with Prieto-Marquez and Wagner, 2009 character 161): (0) strongly constricted, forming a relatively long constricted portion of the basioccipital; (1) faintly constricted, forming a relatively short constricted portion of the basioccipital.

**Basisphenoid**

1. Length of the basipterygoid process of the basisphenoid (corresponding with Godefroit et al., 2001 character 2): (0) very short; (1) relatively long and markedly extended below the ventral border of the occipital condyle.
2. Ratio between the ventral margin of the paired basipterygoid processes of the basisphenoid (modified from Prieto-Marquez and Wagner, 2009 character 162): (0) greater than 100º; (1) up to 100º.
3. Degree of the development of the alar process of the basisphenoid (corresponding with Prieto-Marquez and Wagner, 2009 character 163): (0) moderately developed and relatively small in size; (1) well developed and relatively large in size.
4. Ventral transverse ridge between the basipterygoid processes of the basisphenoid (corresponding with Gates and Sampson, 2007 character 78): (0) present; (1) absent.
5. Ventral median process between the basipterygoid processes of the basisphenoid (corresponding with Gates and Sampson, 2007 character 79): (0) present; (1) absent.
6. Ratio between the maximum width across the spheno-occipital tubercles and the minimum width of the rostral constriction of the basisphenoid (modified from Prieto-Marquez.2009 character 166): (0) less than 1.5; (1) 1.5 to 1.9; (2) greater than 1.9.

**Laterosphenoid**

1. Complete closure of the forward sulcus as the passage of the ramus ophthalmicus of the trigeminal nerve (V_1_) on the lateral surface of the laterosphenoid (corresponding with Evans and Reisz, 2007 character 51): (0) absent; (1) present.
2. Great reduction of the length of the postorbital process of the laterosphenoid (corresponding with Prieto-Marquez and Wagner, 2009 character 168): (0) absent; (1) present.

**Pterygoid**

1. Dorsal expansion of the caudodorsal border of the pterygoid palatine ramus forming a small flange (corresponding with Prieto-Marquez and Wagner, 2009 character 173): (0) absent; (1) present.
2. Ventral expansion of the lamina restricted by two ventrally oriented ridges along the medial surface of the ectopterygoid process and the quadrate ramus of the pterygoid (corresponding with Prieto-Marquez and Wagner, 2009 character 174): (0) moderately extended ventrally, relatively large portions of the ectopterygoid process and the quadrate ramus are located below the ventral margin of the lamina; (1) markedly extended ventrally, only relatively small portions of the ectopterygoid process and the quadrate ramus are located below the ventral margin of the lamina.

**Regional Cranial Characters**

1. General shape of the orbit in lateral view (modified from Prieto-Marquez and Wagner, 2009 character 197): (0) almost circular; (1) elliptic, the rostrocaudal width of the orbit is less than the dorsoventral height of this element.
2. Rostrocaudal width of the orbit relative to that of the infratemporal fenestra (modified from Prieto-Marquez and Wagner, 2009 character 199): (0) the rostrocaudal width of the orbit is approximately equal to that of the infratemporal fenestra; (1) the rostrocaudal width of the orbit is greater than that of the infratemporal fenestra.
3. Position of the dorsal margin of the orbit relative to that of the infratemporal fenestra (corresponding with Prieto-Marquez and Wagner, 2009 character 201): (0) the dorsal margin of the orbit and the dorsal margin of the infratemporal fenestra are basically at the same level and height; (1) the dorsal margin of the orbit is obviously lower than that of the infratemporal fenestra; (2) the dorsal margin of the orbit is slightly higher than that of the infratemporal fenestra.
4. General shape of the infratemporal fenestra in lateral view (corresponding with Xing et al., 2012 character 218): (0) subrectangular in shape; (1) subtriangular in shape.
5. Rostrocaudal width of the dorsal margin relative to that of the ventral margin of the infratemporal fenestra (corresponding with Prieto-Marquez and Wagner, 2009 character 200): (0) the dorsal margin of the infratemporal fenestra is approximately as wide as the ventral margin of this element; (1) the dorsal margin of the infratemporal fenestra is narrower than the ventral margin of this element.
6. General shape of the supratemporal fenestra in dorsal view (modified from Prieto-Marquez and Wagner, 2009 character 202): (0) subrectangular, with the long axis directed rostrally; (1) oval, with the long axis oriented rostrolaterally.
7. Length/height ratio of the skull (the length of the skull is measured from the caudal margin of the quadrate to the rostralmost end of the premaxilla, and the height of the skull is measured from the ventral margin of the quadrate to the dorsal margin of the squamosal) (modified from You et al., 2003 character 1): (0) up to 2; (1) greater than 2.
8. Ratio between the length of the external naris and that of the skull(the length of the skull is measured from the caudal margin of the quadrate to the rostralmost end of the premaxilla) (modified from Horner et al., 2004 character 28): (0) up to 0.25; (1) greater than 0.25 but less than 0.40; (2) 0.40 or greater.
9. Ratio between the maximum mediolateral width of the braincase across the postorbitals and the mediolateral width of the occiput across the quadrate cotyli of the squamosals (corresponding with Prieto-Marquez and Wagner, 2009 character 204): (0) more than 1.25; (1) up to 1.25.
10. Shape of the occiput in caudal view (modified from Horner et al., 2004 character 68): (0) rectangular; (1) trapezoidal.
11. Angle of ventral deflection of the occipital condyle relative to the horizontal line (**new character**): (0) up to 155º; (1) greater than 155º.
12. Basisphenoid participating in the rostroventral margin of the foramen for the trigeminal nerve (**new character**): (0) present; (1) absent.

**Trunk Skeleton**

**Cervical Vertebrae**

1. Number of the cervical vertebrae (corresponding with Horner et al., 2004 character 72): (0) 11 or fewer; (1) 12 or more.
2. Morphological character of the dorsal margin of the axis (corresponding with Prieto-Marquez and Wagner, 2009 character 206): (0) presence of a convex dorsal margin of the neural spine extending to the region above the postzygapophyses; (1) presence of a concave dorsal margin in the caudal region of the axial neural spine.
3. Ratio between the craniocaudal length of the postzygapophyseal process and the craniocaudal width of the neural arch of the cranial and middle cervical vertebrae (modified from Prieto-Marquez and Wagner, 2009 character 207): (0) less than 3; (1) 3 or greater.
4. Morphological character of the postzygapophyseal processes of the cervical vertebrae (corresponding with Horner et al., 2004 character 74): (0) low and relatively short; (1) elevated and relatively long.

**Dorsal Vertebrae**

1. Number of the dorsal vertebrae, not including the last dorsal vertebra (the dorsosacral vertebra) incorporated into the sacrum (corresponding with Xing et al., 2012 character 229): (0) 16 or fewer; (1) 17 or more.
2. Slightly elongated neural spines of the cranial dorsal vertebrae (corresponding with Prieto-Marquez and Wagner, 2009 character 210): (0) absent; (1) present.
3. Ratio between the height and the length of the neural spines of the middle dorsal vertebrae (modified from Norman, 2002 character 41): (0) greater than 2.5 but less than 4.0; (1) 4.0 or greater.
4. Neural spine height greater than 4 times centrum height in the middle dorsal vertebrae (corresponding with Evans and Reisz, 2007 character 67): (0) absent; (1) present.

**Sacral Vertebrae**

1. Number of the sacral vertebrae, including the dorsosacral and caudosacral vertebrae fused to the sacrum (corresponding with Godefroit et al., 2000 character 27): (0) 7 or fewer; (1) 8 or more.
2. Ratio between the height of the neural spine and that of the centrum of the tallest sacral vertebra (modified from Prieto-Marquez and Wagner, 2009 character 209): (0) up to 2.0; (1) greater than 2.0 and up to 3.5; (2) greater than 3.5.
3. Participation of the caudal vertebrae (the caudosacral vertebrae) in the sacrum (corresponding with Xing et al., 2012 character 234): (0) absent; (1) present.

**Caudal Vertebrae**

1. Length of the chevrons relative to that of the neural spines in the cranial region of the caudal vertebrae (corresponding with Wagner, 2001 character 40): (0) the chevrons are shorter than or nearly as long as the neural spines; (1) the chevrons are longer than the neural spines.

**Sternum**

1. Length of the distal handle-shaped process (caudolateral process) relative to that of the proximal plate (craniomedial plate) of the sternum (corresponding with Prieto-Marquez and Wagner, 2009 character 213): (0) the distal handle-shaped process is slightly shorter than or as long as the proximal plate; (1) the distal handle-shaped process is longer than the proximal plate.

**Shoulder Girdle**

**Coracoid**

1. Coracoid size relative to the length of the scapula (corresponding with Horner et al., 2004 character 77): (0) relatively large in size; (1) reduced in size relative to the scapula.
2. Morphological character of the craniodorsal margin of the coracoid (modified from Horner et al., 2004 character 78): (0) straight or convex in lateral view; (1) concave in lateral view.
3. Degree of the development of the biceps tubercle in the cranial region of the coracoid (corresponding with Godefroit et al., 2008 character 47): (0) presence of a relatively small and slightly projecting biceps tubercle; (1) presence of a relatively large and markedly projecting laterally biceps tubercle.
4. Ratio between the length of the lateral margin of the scapular articular surface and that of the lateral margin of the glenoid (modified from Prieto-Marquez and Wagner, 2009 character 215): (0) greater than 1.3; (1) 1.0 to 1.3; (2) less than 1.0.
5. Angle between the lateral margin of the scapular articular surface and the lateral margin of the glenoid of the coracoid (corresponding with Prieto-Marquez and Wagner, 2009 character 216): (0) greater than 115º; (1) up to 115º.
6. Morphological character of the hook-shaped ventral process of the coracoid (corresponding with Horner et al., 2004 character 79): (0) relatively short and directed ventrally; (1) relatively long, recurved and directed caudoventrally.
7. Ratio between the dorsoventral height and the craniocaudal width of the hook-shaped ventral process of the coracoid (modified from Prieto-Marquez and Wagner, 2009 character 218): (0) less than 0.6; (1) 0.6 to 0.8; (2) more than 0.8.

**Scapula**

1. General profile of the dorsal margin of the scapula in lateral view (modified from Sereno, 1986): (0) nearly straight craniocaudally from the cranial margin of the acromial process to the caudal margin of the scapular blade; (1) obviously curved from the cranial margin of the acromial process to the caudal margin of the scapular blade.
2. General profile of the ventral margin along the caudal half of the scapular blade (corresponding with Prieto-Marquez and Wagner, 2009 character 222): (0) almost straight or slightly convex ventrally; (1) remarkably convex ventrally.
3. Orientation of the dorsal margin of the scapular caudal end relative to the ventral margin of this element (modified from Horner et al., 2004 character 81): (0) the dorsal margin of the scapular caudal end is markedly sloping caudodorsally and divergent relative to the ventral margin of this element; (1) the dorsal margin of the scapular caudal end is slightly divergent relative to the ventral margin of this element; (2) the dorsal margin of the scapular caudal end is nearly parallel to the ventral margin of this element, so that they are in the same direction.
4. Ratio between the maximum dorsoventral depth of the scapular cranial end and the distance from the cranial end of the acromial process to the caudal margin of the scapular blade (modified from Prieto-Marquez and Wagner, 2009 character 221): (0) greater than 0.25; (1) up to 0.25.
5. Ratio between the maximum dorsoventral depth of the scapular blade and that of the cranial end of the scapula (corresponding with Prieto-Marquez and Wagner, 2009 character 223): (0) less than 1; (1) 1 or greater.
6. Ratio between the dorsoventral depth of the scapular neck and the maximum dorsoventral depth of the scapular cranial end (corresponding with Prieto-Marquez and Wagner, 2009 character 224): (0) up to 0.6; (1) more than 0.6.
7. Ratio between the distance from the cranial margin of the coracoid facet to the cranialmost end of the acromial process and the maximum dorsoventral height of the scapular cranial end (corresponding with Prieto-Marquez and Wagner, 2009 character 227): (0) less than 0.45; (1) 0.45 or greater.
8. Morphological character of the acromial process of the scapula (modified from Horner et al., 2004 character 80): (0) strongly recurved, with the cranial end of the acromial process oriented dorsally; (1) slightly recurved, with the cranial end of the acromial process oriented craniodorsally; (2) almost straight, with the cranial end of the acromial process directed cranially.
9. Degree of the development of the deltoid ridge on the lateral surface of the scapular cranial portion (corresponding with Prieto-Marquez and Wagner, 2009 character 228): (0) presence of a poorly developed deltoid ridge which is dorsoventrally narrow and relatively faint; (1) presence of a well developed deltoid ridge which is dorsoventrally deep and relatively sharp.

**Forelimb and Manus**

**Humerus**

1. Morphological character of the humerus in caudal view (modified from Weishampel et al., 1993 character 36): (0) relatively long and gracile; (1) relatively short and robust.
2. Degree of the development of the deltopectoral crest in adult individuals (modified from Godefroit et al., 2000 character 26): (0) presence of a poorly developed deltopectoral crest moderately expanded craniolaterally and ventrally; (1) presence of a well developed deltopectoral crest markedly expanded craniolaterally and ventrally.
3. Position of the maximum lateral expansion of the deltopectoral crest relative to the midshaft of the humerus (modified from Horner et al., 2004 character 83): (0) located above the midshaft of the humerus; (1) located at or below the midshaft of the humerus.
4. Strong constriction of the distal half of the humerus below the deltopectoral crest (corresponding with Xing et al., 2012 character 256): (0) present; (1) absent.
5. Ratio between the distance from the dorsal margin of the lateral tuberosity to the maximum lateral expansion of the deltopectoral crest and the proximodistal length of the humerus (corresponding with Prieto-Marquez and Wagner, 2009 character 229): (0) less than 0.48; (1) 0.48 to 0.55; (2) greater than 0.55.
6. Ratio between the width of the humerus at the distal third of the deltopectoral crest and the width of the distal constriction of the humerus (corresponding with Prieto-Marquez and Wagner, 2009 character 230): (0) less than 1.65; (1) 1.65 to 1.90; (2) greater than 1.90.
7. Angle between the craniolateral margin and the ventral margin of the humeral deltopectoral crest (modified from Weishampel et al., 1993 character 37): (0) greater than 130º; (1) 110º to 130º; (2) less than 110º.
8. Ratio between the width of the humerus at the midshaft and the proximodistal length of the humerus in caudal view (corresponding with Xing et al., 2012 character 260): (0) up to 0.20; (1) greater than 0.20 but less than 0.25; (2) 0.25 or greater.
9. Torsion between the proximal and distal ends of the humerus (corresponding with Xing et al., 2012 character 261): (0) moderate, with the angle up to 20º; (1) very strong, with the angle more than 20º.

**Ulna**

1. Ratio between the proximodistal length of the ulna and the craniocaudal width of this element at the midshaft (corresponding with Prieto-Marquez and Wagner, 2009 character 233): (0) less than 10; (1) 10 or greater.
2. Ratio between the proximodistal length of the ulna and that of the humerus (modified from Norman, 2002 character 47): (0) presence of a relatively short ulna, with the ratio up to 1.0; (1) presence of a moderately long ulna, with the ratio greater than 1.0 and up to 1.2; (2) presence of a very long ulna, with the ratio greater than 1.2.

**Carpals**

1. Composition of the carpus (corresponding with Horner et al., 2004 character 86): (0) presence of fused ulnare, radiale, intermedium and distal carpals; (1) presence of two small unfused carpals.

**Metacarpals**

1. Metacarpal I (modified from Norman, 2002 character 49): (0) present and fused to the carpus; (1) absent.
2. Ratio between the proximodistal length and the mediolateral width at the midshaft of the metacarpal III (modified from Horner et al., 2004 character 89): (0) up to 5; (1) greater than 5.
3. Ratio between the proximodistal length and the mediolateral width of the proximal end of the metacarpal V (modified from Prieto-Marquez and Wagner, 2009 character 238): (0) less than 2; (1) 2 or greater.
4. Strong mediolateral expansion of the proximal end of the metacarpal V relative to the distal end (corresponding with Prieto-Marquez and Wagner, 2009 character 239): (0) absent; (1) present.
5. Position of the proximal end of the metacarpal III (corresponding with Horner et al., 2004 character 88): (0) the proximal end of the metacarpal III is aligned with those of the metacarpal II and IV; (1) the proximal end of the metacarpal III is offset distally relative to those of the metacarpal II and IV.

**Manual Phalanges**

1. Morphological character of manual phalanx III-1 in dorsal view (modified from Prieto-Marquez and Wagner, 2009 character 240): (0) moderately elongated proximodistally, with the proximodistal length almost equal to or slightly greater than the mediolateral width; (1) greatly elongated proximodistally, with the proximodistal length obviously greater than the mediolateral width; (2) strongly compressed proximodistally, with the proximodistal length less than the mediolateral width.
2. Manual digit I (corresponding with Norman, 2002 character 51): (0) present; (1) absent.
3. General shape of manual ungual II (corresponding with Norman, 2002 character 53): (0) claw-shaped; (1) hoof-shaped.
4. General shape of manual phalanx III-2 in dorsal view (corresponding with Horner et al., 2004 character 90): (0) rectangular, the medial and lateral margins are subequal in length; (1) wedge-shaped and strongly compressed, the medial margin is significantly shorter than the lateral margin.
5. Proximodistal length of manual phalanx II-1 relative to that of the manual phalanx II-2 (modified from Prieto-Marquez and Wagner, 2009 character 242): (0) manual phalanx II-1 is less than three times as long as manual phalanx II-2; (1) manual phalanx II-1 is three times or more as long as manual phalanx II-2.

**Pelvic Girdle**

**Ilium**

1. Degree of ventral deflection of the iliac preacetabular process (modified from Suzuki et al., 2004 character 69): (0) slightly deflected ventrally, with the angle greater than 150º; (1) markedly deflected ventrally, with the angle up to 150º.
2. Ratio between the craniocaudal length of the preacetabular process and that of the central plate (main blade) of the ilium (modified from Prieto-Marquez and Wagner, 2009 character 244): (0) up to 1.7; (1) more than 1.7.
3. Ratio between the maximum dorsoventral depth of the caudal end of the preacetabular process and the dorsoventral distance from the pubic peduncle to the dorsal margin of the ilium (corresponding with Prieto-Marquez and Wagner, 2009 character 245): (0) less than 0.50; (1) 0.50 to 0.55; (2) greater than 0.55.
4. Ratio between the dorsoventral height and craniocaudal length of the iliac central plate (corresponding with Prieto-Marquez and Wagner, 2009 character 246): (0) 0.8 or greater; (1) less than 0.8.
5. Position of the ventral apex of the supraacetabular process (antitrochanter) relative to that of the caudal tuberosity of the iliac ischial peduncle (corresponding with Brett-Surman and Wagner, 2007): (0) located caudodorsal to ventral apex of the caudal tuberosity of the iliac ischial peduncle; (1) located craniodorsal to ventral apex of the caudal tuberosity of the iliac ischial peduncle.
6. Ratio between the craniocaudal breadth of the supraacetabular process along its dorsal margin and the craniocaudal length of the iliac central plate (modified from Prieto-Marquez and Wagner, 2009 character 249): (0) greater than 0.85; (1) greater than 0.70 and up to 0.85; (2) 0.55 to 0.70; (3) less than 0.55.
7. Lateroventral expansion of the iliac supraacetabular process (modified from Horner et al., 2004 character 91): (0) absent, the supraacetabular process slightly swells laterally along the dorsal margin of the central plate of the ilium; (1) present, the supraacetabular process slightly expands lateroventrally, with its lateroventral margin located at three-fourths of the dorsoventral height of the iliac central plate; (2) present, the supraacetabular process moderately expands lateroventrally, with its lateroventral margin located at approximately half dorsoventral height of the iliac central plate; (3) present, the supraacetabular process extremely expands lateroventrally and nearly overlaps the whole central plate of the ilium in lateral view.
8. General shape of the lateroventral margin of the iliac supraacetabular process in lateral view (corresponding with Prieto-Marquez and Wagner, 2009 character 251): (0) strip-shaped; (1) widely arcuate; (2) U-shaped or V-shaped.
9. Symmetry of the lateral profile of the supraacetabular process (corresponding with Prieto-Marquez and Wagner, 2009 character 250): (0) absent; (1) present.
10. A strong ridge connecting the caudoventral margin of the iliac supraacetabular process with the dorsal margin of the iliac postacetabular process (corresponding with Prieto-Marquez.2009 character 252): (0) present; (1) absent.
11. Lateral profile of the dorsal margin of the ilium above the supraacetabular process (corresponding with Horner et al., 2004 character 100): (0) nearly straight or slightly convex; (1) strongly concave.
12. Morphological character of the pubic peduncle of the ilium (modified from Horner et al., 2004 character 92): (0) relatively long and stick-shaped in lateral view; (1) relatively short and triangular in lateral view.
13. Morphological character of the ischial peduncle of the ilium (modified from Godefroit et al., 2001 character 30): (0) formed by a single large knob; (1) composed of a relatively large knob and a relatively small knob separated by a shallow embayment; (2) composed of two knobs with similar size separated by a shallow embayment.
14. Ratio between the craniocaudal length of the postacetabular process and that of the central plate of the ilium (corresponding with Prieto-Marquez and Wagner, 2009 character 255): (0) up to 0.8; (1) greater than 0.8 but less than 1.1; (2) 1.1 or greater.
15. General profile of the postacetabular process of the ilium in lateral view (modified from Horner et al., 2004 character 93): (0) gradually tapering caudally, forming a wedge-shaped postacetabular process; (1) rectangular, with a straight or arched caudal margin.
16. Brevis shelf at the base of the postacetabular process of the ilium (modified from Godefroit et al., 2008 character 52): (0) present; (1) absent.
17. Mediolateral thickening of the caudal portion of the iliac postacetabular process (modified from Prieto-Marquez and Wagner, 2009 character 257): (0) present, the caudal portion of the postacetabular process moderately thickens mediolaterally on account of the mediolateral expansion of its ventral surface; (1) present, the caudal portion of the postacetabular process markedly thickens mediolaterally triggered by the dorsomedial twist of the postacetabular process; (2) absent, the caudal portion of the postacetabular process is compressed mediolaterally.
18. Orientation of the dorsal margin of the postacetabular process relative to the ventral margin of the iliac acetabular region (modified from Prieto-Marquez and Wagner, 2009 character 260): (0) almost horizontal; (1) oriented caudodorsally.
19. Position of the sacral ridge on the medial surface of the iliac central plate (corresponding with Prieto-Marquez and Wagner, 2009 character 261): (0) located at 50% to 70% dorsoventral height of the iliac central plate; (1) located at 80% dorsoventral height of the iliac central plate.
20. Morphological character of the sacral ridge on the medial surface of the iliac central plate in medial view (modified from Prieto-Marquez and Wagner, 2009 character 262): (0) nearly straight and directed craniocaudally; (1) arched and convex dorsally, with the cranial portion of the sacral ridge directed cranioventrally.

**Pubis**

1. Strong constriction of the caudal neck of the pubic prepubic process relative to the acetabular region (ratio between the dorsoventral depth of the caudal neck and that of the acetabular region of the pubic proximal end) (**new character**): (0) present, with the ratio up to 0.5; (1) absent, with the ratio greater than 0.5.
2. Orientation of the dorsoventral expansion in the cranial region of the pubic prepubic process (corresponding with Prieto-Marquez and Wagner, 2009 character 264): (0) the dorsal portion of the cranial blade of the prepubic process is more expanded than the ventral portion, so that the expansion of the cranial blade is directed dorsally; (1) the ventral portion of the cranial blade of the prepubic process is more expanded than the dorsal portion, so that the expansion of the cranial blade is directed ventrally.
3. Lateral profile of the cranial blade of the pubic prepubic process (modified from Prieto-Marquez and Wagner, 2009 character 265): (0) elliptic, anteroposteriorly longer than dorsoventrally tall, with the dorsal region slightly more expanded than the ventral region; (1) subovate, slightly deeper dorsoventrally than long anteroposteriorly, with the dorsal region much more expanded than the ventral region; (2) circular, with the ventral region much more expanded than the dorsal region; (3) subrectangular and anteroposteriorly elongate, with the ventral region much more expanded than the dorsal region.
4. Dorsoventral depth of the cranial blade of the pubic prepubic process relative to that of the pubic acetabular region (modified from Prieto-Marquez and Wagner, 2009 character 266): (0) the dorsoventral depth of the cranial blade is greater than that of the pubic acetabular region; (1) the dorsoventral depth of the cranial blade is equal to or less than that of the pubic acetabular region.
5. Craniocaudal length of the cranial blade of the prepubic process relative to that of the caudal neck (caudal constriction) of the prepubic process (modified from Horner et al., 2004 character 96): (0) the length of the cranial blade is less than that of the caudal neck; (1) the length of the cranial blade is equal to or greater than that of the caudal neck.
6. Morphological character of the iliac peduncle of the pubis (corresponding with Xing et al., 2012 character 300): (0) relatively short and robust; (1) relatively long and slender.
7. A sharp ridge on the lateral surface of the iliac and ischial peduncles, bounding the cranioventral margin of the acetabulum (corresponding with Prieto-Marquez and Wagner, 2009 character 269): (0) present; (1) absent or bearing a faint ridge.
8. A pubic obturator notch ventral to the ischial peduncle of the pubis for the passage of the obturator nerve (modified from Horner et al., 2004 character 97): (0) present; (1) absent.
9. Length/width ratio of the ischial peduncle of the pubis (modified from Prieto-Marquez and Wagner, 2009 character 271): (0) less than 2; (1) 2 to 3; (2) greater than 3.
10. Lateroventral protuberance in the proximal region of the ischial peduncle of the pubis (corresponding with Prieto-Marquez and Wagner, 2009 character 272): (0) absent or faintly developed; (1) present.
11. Ratio between the craniocaudal distance from the cranial margin of the prepubic process to that of the pubic acetabular foramen and the dorsoventral distance from the dorsal margin of the iliac peduncle to the ventral margin of the cranial end of the postpubic process (modified from Prieto-Marquez and Wagner, 2009 character 274): (0) less than 2.8; (1) 2.8 to 3.0; (2) greater than 3.0.

**Ischium**

1. Caudally oriented curvature of the iliac peduncle of the ischium (corresponding with Prieto-Marquez and Wagner, 2009 character 275): (0) present, the ischial iliac peduncle is slightly curved caudally; (1) present, the ischial iliac peduncle is markedly curved caudally; (2) absent.
2. Angle between the craniodorsal margin and the cranioventral margin of the iliac peduncle of the ischium (corresponding with Prieto-Marquez and Wagner, 2009 character 276): (0) up to 115º; (1) greater than 115º.
3. Ratio between the dorsoventral height of the iliac peduncle of the ischium and the length of the craniodorsal margin of this peduncle (corresponding with Prieto-Marquez and Wagner, 2009 character 277): (0) less than 1.5; (1) 1.5 to 2; (2) greater than 2.
4. Orientation of the cranioventral margin of the ischial iliac peduncle relative to the caudodorsal margin of this element (corresponding with Prieto-Marquez and Wagner, 2009 character 278): (0) the cranioventral margin is either parallel or convergent with the caudodorsal margin along the caudoventral direction; (1) the cranioventral margin is slightly divergent with the caudodorsal margin along the caudoventral direction.
5. Orientation of the craniocaudal axis of the ischial pubic peduncle relative to the ischial shaft in parallel with the level (modified from Prieto-Marquez and Wagner, 2009 character 279): (0) directed cranioventrally; (1) almost directed cranially.
6. Craniocaudal length of the ischial pubic peduncle relative to the dorsoventral depth of this element (corresponding with Prieto-Marquez and Wagner, 2009 character 280): (0) the craniocaudal length is greater than the dorsoventral depth; (1) the craniocaudal length is approximately equal to the dorsoventral depth; (2) the craniocaudal length is less than the dorsoventral depth.
7. A completely enclosed foramen surrounded by the obturator process and the pubic peduncle of the ischium in adults (corresponding with Xing et al., 2012 character 312): (0) absent; (1) present.
8. Location of the craniodorsal corner of the ischial pubic peduncle relative to the dorsal margin of the ischial shaft (corresponding with Prieto-Marquez and Wagner, 2009 character 281): (0) ventral to or at the same level as the dorsal margin of the ischial shaft; (1) dorsal to the dorsal margin of the ischial shaft.
9. Morphological character of the ischial shaft in lateral view (corresponding with Horner et al., 2004 character 98): (0) strongly curved downwards; (1) nearly straight.
10. Ratio between the dorsoventral depth of the ischial shaft at the midshaft and the craniocaudal length of the ischial shaft (corresponding with Prieto-Marquez and Wagner, 2009 character 282): (0) up to 0.05; (1) more than 0.05 and up to 0.075; (2) greater than 0.075.
11. Morphological character of the caudal end of the ischial shaft (modified from Godefroit et al., 2001 character 31): (0) strongly expanded ventrally, forming a large foot-shaped or boot-like protuberance at the caudal end of the ischium; (1) straight and gracile, without the pronounced ventral expansion at the caudal end of the ischium.
12. Orientation of the long axis of the ventral expansion of the ischial caudal end relative to the ischial shaft (corresponding with Prieto-Marquez and Wagner, 2009 character 286): (0) oriented ventrally; (1) oriented cranioventrally.
13. Strong recurvation of the ventral expansion of the ischial caudal end (corresponding with Prieto-Marquez and Wagner, 2009 character 285): (0) absent; (1) present.

**Hindlimb and Pes**

**Femur**

1. Slightly curved caudomedially distal half of the femoral shaft (modified from Norman, 2002 character 62): (0) present; (1) absent.
2. Degree of the development of the lesser trochanter on the craniolateral surface of the proximal portion of the femur (corresponding with Xing et al., 2012 character 320): (0) presence of a strongly developed lesser trochanter; (1) presence of a moderately developed lesser trochanter which is sometimes fused to the greater trochanter.
3. General profile of the caudal margin of the femoral fourth trochanter in lateral view (corresponding with Prieto-Marquez and Wagner, 2009 character 288): (0) triangular; (1) arcuate and smooth.
4. Morphological character of the cranial intercondylar groove in the distal region of the femur (modified from Norman, 2002 character 64): (0) fully open ventrally; (1) nearly or completely enclosed by the lateral and medial condyles owing to the fusion of these two condyles.

**Tibia**

1. Morphological character of the cnemial crest of the tibia (corresponding with Godefroit et al., 2000 character 31): (0) presence of an expanded cranially cnemial crest restricted to the proximal end of the tibia; (1) presence of an extended ventrally cnemial crest along the proximal half of the tibial shaft.

**Fibula**

1. General shape of the distal end of the fibula in lateral view (modified from Godefroit et al., 2000 character 32): (0) subtriangular, forming a moderately expanded cranially fibular distal end; (1) club-shaped, forming a greatly expanded cranially fibular distal end.

**Tarsals**

1. General shape of the cranial ascending process of the astragalus in cranial view (corresponding with Godefroit et al., 2000 character 33): (0) subtriangular in shape and skewed laterally; (1) triangular in shape and equilateral for each margin of the cranial ascending process.
2. Development degree of the articular surface of the astragalus for the internal malleolus of the tibia (corresponding with Prieto-Marquez and Wagner, 2009 character 291): (0) markedly expanded medially, articulating with the whole ventral surface of the tibial internal malleolus; (1) moderately expanded medially, articulating with only part of the ventral surface of the tibial internal malleolus.
3. Distal tarsals II and III (corresponding with Horner et al., 2004 character 102): (0) present; (1) absent.

**Metatarsals**

1. Metatarsal I (modified from Norman, 2002 character 66): (0) present, slender and rod-shaped; (1) absent.
2. Ratio between the proximodistal length of the metatarsal III and the mediolateral width of this element at the midshaft (modified from Prieto-Marquez and Wagner, 2009 character 294): (0) greater than 4.5; (1) up to 4.5.

**Pedal Phalanges**

1. Proximodistal length of pedal phalanx II-2 relative to the mediolateral width of this element at the midshaft (modified from Prieto-Marquez and Wagner, 2009 character 295): (0) the proximodistal length is slightly less than the mediolateral width at the midshaft; (1) the mediolateral width at the midshaft is approximately twice as long as the proximodistal length.
2. Ratio between the mediolateral width at the midshaft and the proximodistal length of pedal phalanges III-2 and III-3 (modified from Horner et al., 2004 character 104): (0) up to 3; (1) greater than 3.
3. Ratio between the mediolateral width at the midshaft and the proximodistal length of pedal phalanges IV-2, IV-3 and IV-4 (modified from Prieto-Marquez., 2009 character 297): (0) up to 3; (1) greater than 3.
4. General shape of pedal unguals in dorsal view (modified from Norman, 2002 character 67): (0) claw-shaped, with the presence of prominent claw grooves; (1) hoof-shaped, with the presence of faint claw grooves or absence of the claw grooves.
5. Plantar median ridge on the ventral surface of pedal ungual (corresponding with Godefroit et al., 2008 character 56): (0) absent; (1) present.

**References**

Brett-Surman M K, Wagner J R, 2007. Discussion of character analysis of the appendicular anatomy in Campanian and Maastrichtian North American hadrosaurids – variation and ontogeny. In: Carpenter K ed. Horns and Beaks: Ceratopsian and Ornithopod Dinosaurs.

Evans D C, 2006. Nasal cavity homologies and cranial crest function in lambeosaurine dinosaurs. Paleobiology, 32: 109–125.

Evans D C, Reisz R R, 2007. Anatomy and relationships of *Lambeosaurus magnicristatus*, a crested hadrosaurid dinosaur (Ornithischia) from the Dinosaur Park Formation, Alberta. Journal of Vertebrate Paleontology, 27: 373–393.

Gates T A, Sampson S D, 2007. A new species of *Gryposaurus* (Dinosauria: Hadrosauridae) from the late Campanian Kaiparowits Formation, southern Utah, USA. Zoological Journal of the Linnean Society, 151: 351–376.

Godefroit P, Zan S Q, Jin L Y, 2000. *Charonosaurus jiayinensis* n.g., n.sp., a lambeosaurine dinosaur from the Late Cretaceous of northeastern China. Comptes Rendus de l’Academie des Sciences de Paris, Sciences de la Terre et des Planetes, 330: 875–882.

Godefroit P, Zan S Q, Jin L Y, 2001. The Maastrichtian (Late Cretaceous) lambeosaurine dinosaur *Charonosaurus jiayinensis* from north-eastern China. Bulletin de l'Institut Royal des Sciences Naturelles du Belgique, Sciences de la Terre, 71: 119–168.

Godefroit P, Bolotsky Y L, Van Itterbeeck J, 2004a. The lambeosaurine dinosaur *Amurosaurus riabinini*, from the Maastrichtian of Far Eastern Russia. Acta Palaeontologica Polonica, 49: 585–618.

Godefroit P, Hai S L, Yu T X et al., 2008. New hadrosaurid dinosaurs from the uppermost Cretaceous of northeastern China. Acta Palaeontologica Polonica, 53: 47–74.

Horner J R, Weishampel D B, Forster C A, 2004. Hadrosauridae. In: Weishampel D B, Dodson P, Osmólska H eds. The Dinosauria, 2nd ed. Berkeley: University of California Press. 438–463.

Kobayashi Y, Azuma. Y, 1999. Cranial material of a new iguanodontian dinosaur from the Lower Cretaceous Kitadani Formation of Japan. Journal of Vertebrate Paleontology, 19: (3, suppl.). 57A.

Norman D B, 2002. On Asian ornithopods (Dinosauria: Ornithischia). 4. *Probactrosaurus* Rozhdestvensky, 1966. Zoological Journal of the Linnean Society, 136: 113–144.

Prieto-Marquez A, Wagner J R, 2009. *Pararhabdodon isonensis* and *Tsintaosurus spinorhinus*: a new clade of lambeosaurine hadrosaurids from Eurasia. Cretaceous Research, 30: 1238–1246.

Prieto-Marquez A, 2010. *Glishades ericksoni*, a new hadrosauroid (Dinosauria: Ornithopoda) from the Late Cretaceous of North America. Zootaxa, 2452: 1–17.

Sereno P C, 1986. Phylogeny of the bird-hipped dinosaurs (Order Ornithischia). National Geographic Research, 2: 234–256.

Suzuki D, Weishampel D B, Minoura N, 2004. *Nipponosaurus sachalinensis* (Dinosauria: Ornithopoda): anatomy and systematic position within Hadrosauridae. Journal of Vertebrate Paleontology, 24: 145–164.

Wagner J R, 2001. The hadrosaurian dinosaurs (Ornithischia: Hadrosauria) of Big Bend tional Park, Brewster County, Texas, with implications for Late Cretaceous Paleozoogeography. 417 pp. Unpubished Master’s Thesis, Texas Tech University, Austin.

Weishampel D B, 1981. The nasal cavity of lambeosaurine hadrosaurids (Reptilia: Ornithischia): comparative anatomy and homologies. Journal of Paleontology, 55:1046–1057.

Weishampel D B, Horner J R, 1986. The hadrosaurid dinosaurs of the Iren Dabasu Formation (People’s Republic of China, Late Cretaceous). Journal of Vertebrate Paleontology, 6: 38–45.

Weishampel D B, Norman D B, Grigorescu D, 1993. *Telmatosaurus transsylvanicus* from the Late Cretaceous of Romania: the most basal hadrosaurid. Palaeontology, 36: 361–385.

Xing H, Prieto-Marquez A, Gu W et al., 2012. Reevaluation and phylogenetic analysis of the hadrosaurine dinosaur *Wulagasaurus dongi* from the Maastrichtian of northeast China. Vertebrata PalAsiatica, 50: 160-169.

You H L, Luo Z X, Shubin N H et al., 2003. The earliest-known duck-billed dinosaur from deposits of late Early Cretaceous age in northwest China and hadrosaur evolution. Cretaceous Research, 24: 347–355.
