# Supplementary material for: A New Basal Hadrosauroid Dinosaur (Dinosauria: Ornithopoda) with Transitional Features from the Late Cretaceous of Henan Province, China
Source: PLoS One. 2014 Jun 5;9(6):e98821. doi: 10.1371/journal.pone.0098821 (PMC4047018; doi:10.1371/journal.pone.0098821)
Supplement: Supporting Information S4 — Character-taxon matrix used in the phylogenetic analysis of Hadrosauroidea. (DOC) [file pone.0098821.s004.doc]

**Supporting Information S4**

**Character-taxon Matrix**

The coded matrix of 61 species for 346 unordered osteological characters (235 cranial and 111 postcranial components) was used in the phylogenetic analysis of hadrosauroids conducted by the software TNT version 1.0 (Goloboff et al., 2008). It was arranged by NEXUS Data Editor (NDE). Subsequently, NDE exported a Hennig86 file holding the data of polymorphism to TNT prior to the cladistic analysis. The maximum parsimony analysis involves one outgroup taxon and 59 ingroup taxa. *Ouranosaurus nigerensis*, which belongs to basal iguanodontians outside of Hadrosauroidea, was used as the exclusive outgroup. This taxon is well described and figured in the literature, and possesses overall morphological information in response to most of the characters in this analysis (Taquet, 1976; Norman, 2004; Paul, 2008). Interrogation marks could indicate the unknown state of the corresponding characters when the missing or distorted anatomical structures derived from the fossil material embarrass the character coding. Brackets indicate polymorphism among the states of a certain character. Hyphens reveal the characters which are inapplicable to some groups or taxa. The data matrix used here was updated, in order to acquire a more credible phylogenetic tree topology of Hadrosauroidea, especially the databases of the following three taxa that were greatly revised, namely *Nipponosaurus sachalinensis*, *Lophorothon atopus*, and *Charonosaurus jiayinensis*.

***Nipponosaurus sachalinensis***

We concur with Suzuki et al. (2004) and Evans and Reisz (2007) in considering *Nipponosaurus sachalinensis* indicative of a non-adult lambeosaurine individual. Some characters of the species probably change through ontogeny so as to confound the morphological phylogenetic analysis, such as the number of dentary teeth per centimeter (character 2) and the height/width ratio of the dentary tooth crown (character 5) (see Supporting Information S3). They are therefore regarded to be the missing data in the character matrix.

***Lophorothon atopus***

The widely open sutures of the bones on the skull roof and relatively small size indicate that the holotype of *Lophorothon atopus* (FMNH P27383) is probably from a juvenile individual. Considering the immature identity, each character state observed in the holotype of the species has been carefully identified. The character coding of *Lophorothon atopus* for the present phylogenetic analysis is only based on the holotype described in the literature published by Langston (1960). It is unclear whether the additional postcranial information of *Lophorothon atopus* in the data matrix of Prieto-Márquez (2010) is referable to this species.

***Charonosaurus jiayinensis* (*Mandschurosaurus amurensis*)**

According to the information of regional geological survey of Jiayin area, both *Mandschurosaurus amurensis* and *Charonosaurus jiayinensis* were recovered from the upper part of the Longgushan outcrop section within the Yuliangzi Formation. These two hadrosaurid members are definitely problematic owing to the unmeant mixture of several different individuals and taxa into one erected taxonomic unit (Riabinin, 1930; Yang et al., 1986; Godefroit et al., 2000, 2001). Much of the available material referring to the two species in the literature was collected from the same horizon, namely the third dinosaur-bearing layer. Recently, the hadrosaurid material known from the preceding horizon has undergone the preliminary culling and re-evaluation. The picked specimens are quantitatively dominant in the quarry, and account for 75% of the total number of the bones. These specimens probably pertain to a valid lambeosaurine taxon, and include the partial braincase and postorbital described by Riabinin (1930), as well as the holotype of *Charonosaurus jiayinensis*. Except for the specimens assigned to the valid lambeosaurine dinosaur and the ambiguous material, the other specimens could be assigned to one or two hadrosaurine taxa, which are excluded from the data matrix used in the phylogenetic analysis. Here we tentatively use “*Charonosaurus jiayinensis*” to denote the valid lambeosaurine dinosaur recovered from the upper part of the Longgushan outcrop section within the Yuliangzi Formation. However, it is incontestable that the material of *Charonosaurus jiayinensis* described by Godefroit et al. (2001) consists of some different individuals belonging to lambeosaurines and hadrosaurines, respectively. The data of the valid lambeosaurine taxon recovered from the Longgushan outcrop section within the Yuliangzi Formation have been revised for the current cladistic analysis.

Here we exclude *Hadrosaurus foulkii* from the phylogenetic analysis carried out in this paper because this taxon is tentatively regarded as a *nomen dubium* that lacks any diagnostic characters. *H. foulkii* was erected based on fragments of the maxilla and the partial postcranial skeletons (Leidy, 1858). Only the humerus, ilium, and ischium of this taxon can provide significant morphological information that is transformed into the data for the phylogenetic analysis. The ilium and ischium of *H. foulkii* display a series of striking features that are typical of *Edmontosaurus* and *Shantungosaurus*, such as an anteroposteriorly shortened iliac supraacetabular crest, the symmetrically U-shaped lateroventral edge of the supraacetabular crest at the half height of the central plate of the ilium, the poorly defined posteroventral margin of the supraacetabular crest that connect the anterodorsal margin of the postacetabular process, no evidence of the posteriorly directed curvature of the iliac peduncle of the ischium, and the elevated anterodorsal corner of the pubic peduncle of the ischium relative to the ischial shaft. The most pronounced character of the humerus assigned to *H. foulkii* is the weakly developed deltopectoral crest with a wide arcuate laterodistal corner (Prieto-Márquez, 2011). Prieto-Márquez (2011) regarded the short deltopectoral crest (whose length is less than 48% of the length of the humerus) and the wide arcuate laterodistal corner of the configuration as plesiomorphic for Hadrosauroidea. However, it is undeniable that the wide arcuate laterodistal corner of the deltopectoral crest leads to the difficulty in the confirmation of the ventral limit of the deltopectoral crest, and possibly misleadingly makes the deltopectoral crest shorter. In fact, the “short” deltopectoral crest with a wide arcuate laterodistal profile can be also observed in sporadic specimens of *Shantungosaurus*, *Brachylophosaurus*, and *Edmontosaurus*, where the deltopectoral crest is approximately 45–48% as long as the humerus. The presence of the configuration in hadrosaurines may be attributed to either intraspecific variation or anomalous preservation. Therefore, we agree with Prieto-Márquez et al. (2006) in considering *H. foulkii* to be a *nomen dubium*.

*Bactrosaurus johnsoni*

0111(12)100212?1110(01)111(01)010001000011010001211101(01)00(01)000000111110001000111110000000110000000000--00?0(01)10(12)0010(01)0010010110000000200011?011100000000000000?100?00000?0?0000--00--100000000000001010100000000000000000000011011000100??000000??0100?001??00011?10002000000010011000000(01)0010??101???1??1020011100011000001000001001110011111001201011111000?1000010

*Claosaurus agilis*

????1300??1????????11?1????????????????????????????????????????????????????????????????????--??????????????????????????????????????????????????????????????????????????????????????????????????????????????????????????????????????????????????????0?1?????????00000002000000000000???????????0?111112?00?1111?0???????????????????????????????????1110???

*Eolambia caroljonesa*

01110300??10?0100?0??01??????????????00102101(01)000?0?0001?10000000000?1111?00000000000000000--00?00?0??????0????10??0?000?01?0020?0000000?0000000?000100?00000?0??000--00--??????00000000??0000000000000?001000000111110000100????0??????110??????000?0????????????????????????????????????????10???0000001???000?????????????0111010011000???0????????????

*Equijubus normani*

00110000??00???00??00000101010000000?00102?01?000?0?0???0100000100?0?0000000000010000000000--000000010??000010?000?000000000001?000000000000000000021001000001000000--00--1000000?00000000001??0000000???00?0???0001???0???0???00000?001?00000100??0?0????????????????????????????????????????000000000000000??0??????????????????????????????????????????

*Gilmoreosaurus mongoliensis*

01112???????11?001111010??0?0001101??0??010010???0?0000111?????????????????????????????????--?0???10200101001?011110??0000??00???0011000???00000000???????000?0?????--?0--100???0?00???????????????00????????????????????????????????????????01??00??????002000000??001100000000010??1????????1021011100(01)120100010?????001?1?011111001100011111000?1000000

*Iguanodon bernissartensis*

000000000000000001000000(01)000000000000001100000000000000000000000000000000000000000000000000--00000000000000000000000000000100000000000000000000000000000000001000000--00--0000000000000010001000000000???010000000000000??10000000000000000000000001100000000000000000000000000000000000000000100(01)00000000010000000000000000000000000010000000000000110000

*Jintasaurus meniscus*

?????????????????????????????????????????????????????????????????????????????????????????????????????????????????????????????????????????0?0?0??????1???????????????--????????????0011001?001000000000000110010101110?000?100????00?1??1100???????????????????????????????????????????????????????????????????????????????????????????????????????????????

*Jinzhousaurus yangi*

00000000??00?0000??0000??0?00????0?0000102??0?00??0??0?00100000000?0???0?100000?00000000000--0?0?0?000?0000010000??00000002000000000?0?00000000000001001000001010000--00--0000000000000000001000000000???000????????????????000000001011?0?000000000100000000000000000000000000000000000000000000000000000010000000000100000000000000110000000000001000000

*Levnesovia transoxiana*

00??110?111011100?11101??010000???1??001011010???????00?11110?010000??????????????????????????0??0?0??010100?0010110?00000???001??111?00???????????????????????????????????00000000011001?10000000000000001000000011110000100????00?0??1100????????0?1????0100??????????00000??0???????????????????101000??????????00??011?1??????????????1??1??????????10

*Mantellisaurus atherfieldensis*

00000000000000000(01)0000000000000000000011010000000000000000000000000000000000000000000000000--00000000000000000000000000000100000010000000000000000000001000001000000--00--0000000000000010001000000000???000000000100000??100000100000000000000000001000000000000000000000000000000000000000000(01)0(01)00000000000000000000000000000000000010000000000000000000

*Nanningosaurus dashiensis*

01??1101111?11100?1110???????????????????2????011101100111??????????1?????????????????????????0?0000101100001?11111???????????????????11????????????????????????????????????????????????1?101?????????????????????1?1????????????????????????11?????????????????????0010?????????????????????????????????????????????????????100001001?00011111???????????

*Nanyangosaurus zhugeii*

?????????????????????????????????????????????????????????????????????????????????????????????????????????????????????????????????????????????????????????????????????????????????????????????????????????????????????????????????????????????????001110?????????????????00100011010?11000111?????????????????????????????????????????1?0001111100011000010

*Ouranosaurus nigeriensis* (**outgroup**)

0000000000000000000000?200100000000??010020100000000000000000000000000000?00000000000000000--0?00?000000010000003200?00000000000000000000000001000000020000001000001--00--000000000011000000?000000000???01000000010000000100000100011001000100?11102000010000010000000000000000000000000000000120000000000100000000000000000000000001100000000000?1000000

*Probactrosaurus gobiensis*

001111002100?1100??1?0?000?000011?1??01101101?0000000001110???0????0???00000000?00000000000--000???????0??0??0??00?00000000??0?0???????????000000???????00000?0?????--00--??????0?00???00?00?000000000???0(01)000??0??????????0???0000000?1?00??????000?0?000010000000000000000000001??01???001??0?0000000000000000100000?00???000100100110000000??????000000

*Protohadros byrdi*

001?01(01)01110111101??10100000??1131200101221?100000000011010100000000???11100000000000000000--0010010100101001?01011000000010002000100?00?0000000?000?001000001000000--00--?00?000?001?0????????0000000??0????????????????????????000?0??????????????????????????????????000000000?????????????????????????????????????????????????????????????????????????

*Shuangmiaosaurus gilmorei*

0??????????????00111101??????????????01012000000?0?0000111???????????????????????????????????????0000001???01001111?00????????????????????????????????????????????????????????????????????????????????????????????????????????????????????????????????????????????????????????????????????????????????????????????????????????????????????????????????????

*Tanius sinensis*

???????????????????????????????????????????????????????????????????????????????????????????--??????????????????????0?00000000011?01??0???0000?00??????????????0???????????????0?0?0011001010?0?000000000?0100000001111000?10000?00000??1100?101???0?1??????????100000000000000100?????????????10200101000111100000????????????????????????10???1??????????

*Telmatosaurus transsylvanicus*

011?1200??0?11101?11100??????????????000000110001101001111111?11010111111000000000000000000--00??010200101101002111?0?0000???001?01??01110000000???010?1000001000000--00--??????0?00???????0??000000000??00?0??1??11111???100????000000??10?011????????????????????????????????????????????????????????????????????????????????????????????????0??????????

*Tethyshadros insularis*

01111101??0011101111100?001000?11?1000?00???1?001?0???111111101101?11111?000000000000000000--00??01021?1011?10?2???000??0000002?001111?11000000000001001000001000000--00--1000000000110010100?00000000????0000??0111???????????000000101???00110000111110001000100010???00000000?10111??0011111?2111320001?10120??00010011110??111?0?101--11??1???11001?10

*Xuwulong yueluni*

00110000??00???00??100?00?1000000000100102001000000?0001110?00010000?0000000000110000000000--000000010??000010?0?0?00000002?001?01100000000000000000?0000000010300?0--00--1000000000000010001000000000???000000000110??0???000?000000000100000100000??1???????????????????????????????????????000000000000000000??000010000100000010012000????????????????

*Zhanghenglong yangchengensis*

01211210?11?11100111111??????????????01202011000???0011111????????????1???????????????????????0?01101001011010121210?012?12?0001??????????????????????????????????????????????????????????????????????????????????????????????????????????????1?1??????????????101000010?????????1????????????????????????????????????????????????????????????????????????

*Acristavus gagslarsoni*

1122131??12?11111121111??????????????21(12)000111011101111211111?10010111112?31101?00000000000--00?1010210211111112121001121113121110011111100000001?10001?0000000?11?0--00--2000000100(01)(01)001110?120000000?1012021111011110100200???100?0??0111??????????????????????????????????????1????????????????????????????????0????11101??????????????1111????????????

*Brachylophosaurus canadensis*

11222311212?111111211(01)120121112120211312(01)(12)11110111111112111110100101111121311011(01)0000000000--00110102102111111122211011211231(12)1110111011101100301112001(12)000000011101--00--2000010100(01)(01)0011100111011001??012021111011110100200101100000101111111100011111111211210111102100101011012111101111111120122201112111211111301111111(02)11112(01)1111--1111100011000011

*Edmontosaurus annectens*

213313102121111211211?21(01)111?11130211(23)100(01)01011221011112111110200101111121301111(01)1000000000--00111002102111111123211101201020211101111111000000022121020000001011210--00--2001000100121111102110000000010121211120111100001010111011010010110111000101111112112101111021001011111(01)1111111011111(01)2113221111211121111120011110221211211101--1111100011000010

*Edmontosaurus regalis*

2133231021211112112110210111?11130211(13)100(01)01011221011112111110(12)0010111112130111101000000000--00111102102111111123211101201020211101111111000000022121020000001011210--00--200100010012111110211000000001012121112011110000101011101100101011011?0001011111121111011110210010112111111111101111102113221111211121111120011110221211111101--1111100011100?10

*Edmontosaurus saskatchewanensis*

213313102121111211211?210111?111302113100(01)0101122101111211111020010111112130111101000000000--00111002102111111123211101201020211101111111000000022121020000001011210--00--2001000100121111102110000000010121211120111100001010111011010010110111000101111112112101111021001011111111111110?1111121132211112111211111200111?0221211211101--1111?00011000010

*Gryposaurus incurvimanus*

11231310??2?111101211??0002101013?210202110111012101111211111010010111111120101100000000000--10011203102111110123211111100121121101110111000002001102122000001031101--00--200100010011001100211000000001012021112011110000100001010000111111011100010111111211210100112100101121011111111011111111112211112211211?11301111?0221111201101--1111110011100110

*Gryposaurus latidens*

11230311111011110?211????????????????2021201110121011112111110100101111121201??100000000000--?001120310211111012321???11001??121????????100000200110?12200000?0?1101--00--?00???01?????0???02??000?????????????????????????????1??00?????11?011????10111111211210100112100101(01)(12)1001??1????????101?112210112211211111301111?02??????????1--?????1????????10

*Gryposaurus monumentensis*

11231310??2?111201211020011101013121020221011101?101111211111020010111111120101100000000000--10011203102111110123211111101221221101110111000002001102222000001031101--00--20010001001100110021?000000001?12?2111?011?10???10000111000021111???????????????????????????????????????????????????????????????????????????????????????????????????????????????

*Gryposaurus notabilis*

11231310112?111101211??00021010131210(12)02(12)(01)0111012101111211111020010111111120101100000000000--10011203102111110123211111100121121101110111000002001102222000001031101--00--20010001001100110021100000000101202111201111000010000101000011111101110001011111121121010011210010112101111111101111111111221111221??11111301?11?0221111201101--1111110011100?10

*Kerberosaurus manakini*

???????????????21?211?????????????????????????????????????????????????????????????00?00????-??0??110210211111?123211101200020211?0111?11??0000?0????????00000?0?121?--00--?0010001?????????0?1?00000?0??012021??2?1?110????0??????????????????????????????????????????????????????????????????????????????????????????????????????????????????????????????

*Kritosaurus navajovius*

11232210??2?1111112110?00?1?1101212??00120011101210111121111101001?1111????0??????000000000--0001120210211111?1232111111002211211011101110000020011011??00000?0?1101--00--2001000100110011002100000000???(01)2021112011110000100?01010000?1111??11???????????????????????????????????????????????????????????2???????????????????????????????????????????????

*Lophorhothon atopus*

????22102120??1????1101???????????????????????????????????????????????????????????00000000?--?0????02???111??0??????10??00????????112?11?0000060????10??00000?0?1221--00--?00?0001001100??1021?00000000?0??0???120111???????0?0110??0??1111??????00???????????????????????????????????????11?????????????????????????????????2121??????1--11?11000?1100010

*Maiasaura peeblesorum*

11222311212?111111211??20121112120211202110111011111111211111010010111112131101110000000000--00110102102111111122211011211231(12)11100110111012005011120011000000001101--00--2000010100(01)(01)001110012003000001?1202111101111010020010110000000111111110001111?111211?10111102100101011011111?01111111120122201112111211111301111111(02)1111201111--11111000110?0011

*Prosaurolophus maximus*

(12)1232310112?1112112110?01121011131211110(01)001011(12)2101111211111020010111112120101100000000000--00011(01)02102111110123211101200(01)20211101121111021006022121021000002021221--00--20010(01)01001101111021100000110101202111201111000000000110110(01)111111?11?0001011111121121011111210010112211111111101111102112(23)21011221121111121011120221211211111--1111100011001110

*Saurolophus angustirostris*

11231310??2?111211211??01121011131211(12)10000101112101111211111020010111111120101100001000100--00011102102111110123211101200020211101121111021004022121021000002021221--00--200001011011011110212102001122?1202111201111?0??00000110110111111111111001011111121111011110210010(12)222101111??1011111021123211112211211?11200111?0021211211111--1111110011101110

*Saurolophus osborni*

21232310112?1112112110?0113101113121?210000101112101111211111020010111111120101100001000100--00011102102111110123211101200020211101121111021004022121021000002021221--00--200001011011011110212102001122?1202111201111?0??00000110110111111101111001011?111211110111102100101222101111??1011111021123211112211211?11200111??021211211111--1111110011101110

*Secernosaurus koerneri*

1?2?2310??2?111111?110???1?1?????????0021111110?2101?112?11????????1111???????????????0????-??0??1203102??1????2121?????????????????????????????????2??????00?0???????0??-?0010001001200???0?1?00000?0??01202111201111000?000???????0??1?1???11??00?1??11111012101001121?????????1???1????????1111112200112111111111311111202211112011?1--11111????11?????

*Shantungosaurus giganteus*

21331311??2?11121?211???0?2??????????110020111112101111211?????????1111??1?0??????00000000?--?0?1100210211111??2?21??????????????1111111?0000000???????000000?0??210--00--?00000010012011?10211000000001012121112?111?????10????101?00?01(01)1??11?000111111112112101??102100101121111??1????????102113221111211121111120011110021211201111--11111000?1100?10

*Wulagasaurus dongi*

11??????????11???????????????????????11101011?01?1?1111210????????????1????????????????????-????????????????1??????10?12???3??11?01111?1???????????????????????????????????????????????????????????????????????????????????????????????????????????????111121121011?1021001010110?????????????10??1222011?2?112111???????????21111201111--?????0??????????

*Amurosaurus riabinini*

11232211212?11111121101??????????????1022211110111011112111111111111111111110001001320??1?111-1--101201311121?2232113?121101020110001111110342-14-211-2?11111-?--00132?????12010?11111001011112111111112103031110111111000100?01????1?21?11??11??10????01112111112?1001011112222112??1????????102?1322101?2?????1110100111110111112?0110??11111101?10???10

*Aralosaurus tuberiferus*

???????????????11??110??????????????????????????????????????????????1?????????????000000000-??1--000201201121?12121111???11?020??11111??110110-0?-??0???00?00?0????10000???00000010011001?10?0?000000000?0?011110?1????????????110001??1?1??????????????????????????????101?11?10????????????????????????????????????????????????????????????1????????????

*Arenysaurus ardevoli*

112?2211211?111?1????????????????????100121101011101111211111?111111111????????????????????????????????????2?????2????????????0??????????10??????-??1??????????????????????12110?110110010111121131111?2?0301111011111100?100???1?0?1??1111??11??10???0?1?1??1?100?1??2???????????????????????????????????????????101??1??????????????????1??1????????????

*“Charonosaurus” jiayinensis*

11233311212011111??111???????????????(01)021211100111011112111110111111111???????????1??????????-1--11110?311121??23211321211010201110111111103?2-??-?11-??111?1-?--001???????12010?110110011212121230112121030?1110011111001100??112??1??11111?111110111?011121111121110111111222201?1?1011??11110201322101120112111101001111?0111112001101111111111?10?1?10

*Corythosaurus casuarius*

112322112110111111211??01132111140210111(12)(01)111(01)01110111121111102111111111111100010012201110111-1--(01)012013111211223211321211(01)1021111000111110332-15-211-2(01)11111-1--00132111131201011111100101111211111111210301111001111101100000112001021111101111101111111121111121110101111222211211111101111112013221011211121111010011111011111210120011111110111011110

*Corythosaurus intermedius*

112322112110111111211??01132111140210111(12)1111(01)01110111121111102111111111111100010012201110111-1--1012013111211223211321211(01)1021111000111110332-15-211-2(01)11111-1--0013211113120101111110010111121111111121030111100111110110000011200102111110111110111?111121111121110101111222211211111101111112013221011211121111010011111011111210120011111110111011110

*Hypacrosaurus altispinus*

11233211212?11111121112?113221114021?1111(12)111101110111121111102111011111111100010012201210201-1--1012013111211223211321211(01)1021111000111110332-15-211-2111111-2--0013211113120101111110011111121111111121030111100111110100(01)0001120010111111111?1111210111121111121100101111222211211111101111112013221011211111111010011111011111210120011111110111111110

*Hypacrosaurus stebingeri*

112332(01)02110111111211??1113211114021010112011101110111121111102111011111113100010012201110111-1--101201311121122321132121111021111000111110332-15-211-2111111-2--001321111312010111111001111112111111112103011110011111011100??112001021111??111?11121?1111211111211001011112222112???????????112013221011211121111010011111011111?101200111111?01?1111110

*Jaxartosaurus aralensis*

?????????????????????????????????????????????????????????????????????????????????????????????????????????????????????????????????????????10???-??-?11-???11?1-?--001???????11????10011001?102121111110???03011110?111??0???00???10??1??1?11???????????????????????????????????????????????????????????????????????????????????????????????????????????????

*Lambeosaurus lambei*

1123(23)21121101111112111101?2101114021011111011(01)01110111121111102111111111111100010013201211112-1--0012013111211223211321211(01)1020111001111110342-14-211-2111111-1--00132112131201011111100101111211111111210301111001111100011000112001021111101111101110111121111121100101111??22112111111011111(01)201322101121112111101?011111111111210110011111110111011110

*Lambeosaurus magnicristatus*

11?3?211????11111121111?112?01114021011111??11011?0?11121111101111?11111111100010013201211112-1--00120131112112232?132121101021111001111110342-14-211-2111111-1--001321121312010111111001011112111111112?03?11110011???????1???11200102?1111?11?110????11??????11211001011112222112111??1011111020132210112111211?101001111?1111112?012001111111??110???10

*Magnapaulia laticaudus*

11?3221121??11111?211????????????????????????????????112?????????????11111100001001?????1?10?-1--001201311121??232113212111?02???????????103??-??-21?-2?111?1-?--001?????????????1?????????????1?????????????????????????????????????????????11?1???2???1112111??????01?111121221?????????????1020132210112?1??111101?01111101111121001011?????????1??????

*Nipponosaurus sachalinensis*

??22?211??2???1??121112???????????????????????0111011??21111102????1111??1????????1?????1????-1--???????????1?????1????????????1?????????103?2-??-211-??111?1-?--001?????????????1?????????????1??????12????????????????????????????????????011?????????11120??01?110010?????????1??11???01111?????312101???1??111??????????????11?001?00?11???????1?11?10

*Olorotitan ararhensis*

11?3221121???11111211??1113111114?1?021111111001110111121111102111111111111100010012201110200-1--0011013111211223211421211010211?1???1??110332-1?-211-2111111-?--0013??1??31201011???????????1?11???1112??3???????11?????????0011200?02??1110111110111?11112111112111010111122221?????????????1120132210112111211??????1111??111112001100?11111???????????

*Pararhabdodon isonensis*

11??????????11111????????????????0???100222101??????111211???????????11???????????????????????????0010?4111?1?12321??????????????????????????????????????????????????????????????1???????????????????????????????????????????????????????????11????111?????????1?????010111122220????????????????????????????????????????????1????????????????????????????

*Parasaurolophus cyrtocristatus*

112222112110111???????????????????1???????????0??1?1?1121111101????1111???????????14010000??0-1--1????1???????????????1?????????????????110342-04-211-2211121-?--001220100312010111011001?212121210112?2?03011110011111001100??112??10?1?111?11?1101110????????10011101111112122011111??1?1???1021133210112011211110100111110100011001201111111111110???10

*Parasaurolophus tubicen*

11233(23)11??2011111121111???????????1??1121211100111011112111110111111111???????????14010000??0-1--1111013111210223211(23)21211010(23)0111?11111110342-04-211-2211121-?--0012201003120101110110011(12)12121230112?2103011110011111001100??112??1??1111????????????????????????????????????????????????????????????0???????????01???111???????????????????????????????

*Parasaurolophus walkeri*

11233???????111?1??111111121?1113011010202111?01?1?1111211111011111111111131000?0014010000200-1--1111013111?10?232?1221?11010301110??111110342-04-211-2211121-?--0012201003120101110110011112121220112?2103011110011???????0???11200102111110111110111??11121111121110111111222201111101121111102013221011201121?1101001111?0111112001101111111???11?????0

*Sahaliyania elunchunorum*

11232211212?111?1?21111??????????????102221111011101111211111111111111111?1100010013201?1?111-1--101?0?311121??2321132121101020111101111110342-??-?11-??111?1-?--0013??????12010?1??????1?11112111111112103031110111111000100?01????1?2??11??11??101?1?01112111112?1001011112222112??1?????1??0020132210112111211110100111110111112?01100?11111101?10???10

*Tsintaosaurus spinorhinus*

1122231111101111112110?10121011140110(12)002221010(12)1101111211111011(01)10111110130001100110000?00?0-1--000101411121?123211?212?1110301?101(12)111110121-03-210-2?11101-?--0011001???10?00010011001?102100000110???0301111011111100?100??110??10?11111?11?110111001112010100110010111(01)22220121?1011??1?11021132200112011(12)11110100111110111111001100111111101?1011110

*Velaphrons coahuilensis*

11??221?????11111?211??11122?1114?1??012111111011101111211?????????11111111100010012201010101-1--1012013111211122211321211110201?1100111?10332-15-211-2111111-1--001311111?12????111??001?1??121111?1112?03011110??????????????11?001021?????????????????????????????01?1111?2221????1????????112013121011211111111????11111?1111121011001?????????10???10

*Hadrosaurus foulkii* (regarded as a *nomen dubium* in this paper)

?1??131?112???1????1101??????????????????????????????????????????????????????????????????????????????????????????2???????????????????????????????????????????????????????????????????????????????????????????????????????????????????????????????????????????1??????????000001???????????1?21132211112??12111?????11????21111??1?????111?1???????????

**References**

Evans D C, Reisz R R, 2007. Anatomy and relationships of *Lambeosaurus magnicristatus*, a crested hadrosaurid dinosaur (Ornithischia) from the Dinosaur Park Formation, Alberta. Journal of Vertebrate Paleontology, 27: 373–393.

Godefroit P, Zan S Q, Jin L Y, 2000. *Charonosaurus jiayinensis* n.g., n.sp., a lambeosaurine dinosaur from the Late Cretaceous of northeastern China. Comptes Rendus de l’Academie des Sciences de Paris, Sciences de la Terre et des Planetes, 330: 875–882.

Godefroit P, Zan S Q, Jin L Y, 2001. The Maastrichtian (Late Cretaceous) lambeosaurine dinosaur *Charonosaurus jiayinensis* from north-eastern China. Bulletin de l'Institut Royal des Sciences Naturelles du Belgique, Sciences de la Terre, 71: 119–168.

Goloboff P A, Farris J S, Nixon K, 2008. TNT, a free program for phylogenetic analysis. Cladistics, 24: 774–786.

Langston W D, 1960. The vertebrate fauna of the Selma Formation of Alabama. Part 6: the dinosaurs, Fieldiana, Geology Memoirs, 3: 313–363.

Leidy J, 1858. *Hadrosaurus foulkii*, a new saurian from the Cretaceous of New Jersey, related to Iguanodon. Proceedings of the Academy of Natural Sciences of Philadelphia, 10: 213–218.

Norman D B, 2004. Basal Iguanodontia. In Weishampel D B, Dodson P, and Osmólska H (editors), The Dinosauria, 2nd ed. Berkeley: University of California Press, 413–437.

Paul G S, 2008. A revised taxonomy of the iguanodont dinosaur genera and species. Cretaceous Research, 29: 192–216.

Prieto-Márquez A, 2010. Global phylogeny of Hadrosauridae (Dinosauria: Ornithopoda) using parsimony and Bayesian methods. Zoological Journal of the Linnean Society, 159: 435–502.

Prieto-Márquez A, 2011. Revised diagnoses of *Hadrosaurus foulkii* Leidy, 1858 (the type genus and species of Hadrosauridae Cope, 1869) and *Claosaurus agilis* Marsh, 1872 (Dinosauria: Ornithopoda) from the Late Cretaceous of North America. Zootaxa, 2765: 61–68.

Prieto-Márquez A, Weishampel D B, Horner J R, 2006. The dinosaur *Hadrosaurus foulkii*, from the Campanian of the East Coast of North America, with a reevaluation of the genus. Acta Palaeontologica Polonica, 51(1): 77–98.

Riabinin A N, 1930. *Manschurosaurus amurensis* nov. gen. nov. sp., a hadrosaurian dinosaur from the Upper Cretaceous of Amur River, Mém. Soc. paléontol. Russie, 2: 1–36.

Suzuki D, Weishampel D B, Minoura N, 2004. *Nipponosaurus sachalinensis* (Dinosauria: Ornithopoda): anatomy and systematic position within Hadrosauridae. Journal of Vertebrate Paleontology, 24: 145–164.

Taquet P, 1976. Osteology of *Ouranosaurus nigeriensis*, iguanodontid from the Lower Cretaceous of Niger, Geology and paleontology of the Gadoufaoua fossil locality. Aptian of Niger. Cahiers de Paleontologie. Paris: Centre National de la Recherche Scientifique, 1–191.

Yang D S, Wei Z Y, Li W R, 1986. Report of preliminary excavation of dinosaur fossils from the Cretaceous of Jiayin County, Heilongjang Province. Natural Research of Heilongjiang Province, 2: 1–10.
