# Supplementary material for: A New Basal Hadrosauroid Dinosaur (Dinosauria: Ornithopoda) with Transitional Features from the Late Cretaceous of Henan Province, China
Source: PLoS One. 2014 Jun 5;9(6):e98821. doi: 10.1371/journal.pone.0098821 (PMC4047018; doi:10.1371/journal.pone.0098821)
Supplement: Supporting Information S5 — Original information in the analytical process of the program TNT, with the strict consensus tree and list of synapomorphies. (DOC) [file pone.0098821.s005.doc]

**Supporting Information S5**

**Original Information in the Analytical Process of the Program TNT, with the Strict Consensus Tree and List of Synapomorphies**

This appendix shows much of the original information of the maximum parsimony analysis in the analytical process of the program TNT. In addition, the strict consensus tree with node numbers is followed by the list of unambiguous synapomorphies that support each of the nodes present in this topology. The ordered numbers representing the nodes of the strict consensus tree were clearly distributed in the following image (Fig. 1). They were used as the substitutes for the initial, chaotic ones in the EMF file of the strict consensus tree, as well as the corresponding ones in the list of synapomorphies exported from the program TNT.

Abbreviations: MPTs, most parsimonious trees; SCT, strict consensus tree.

**Original information showing the concrete operation of the phylogenetic analysis of Hadrosauroidea in the user interface of the program TNT**

Reading from C:\Users\Hai Xing\Desktop\Zhanghenglong.ss

'Exported from NEXUS Data Editor'

Matrix (346 × 61, 16 states). Memory required for data: 0.31 Mbytes

Space for 10000 trees in memory

Not collapsing trees temporarily for consensus calculation

Ignore unshared taxa is ON

Outgroup is taxon 9 – Ouranosaurus_nigeriensis

0 trees in memory

Random seed is 1

Repl. Algor. Tree Score Best Score Time Rearrangs.

1000 TBR 54 of 54 ------ 1004 0:00:38 473,779,899

Completed 1000 random addition sequences.

Total rearrangements examined: 473,779,899.

Best score hit 1000 times out of 1000.

Best score (TBR): 1004. 54 trees retained.

Time 38.32 secs.

Strict consensus of 54 trees (0 taxa excluded) calculated, as tree 54

Time 0.02 secs.

Tree lengths

1004 (MPTs) 1038 (SCT)

Time 0.02 secs.

Reading from E:\TNT\WSTATS.RUN

Macro language is ON

Macros: 42.7 Kb in use, 59.6 Kb free

Report is ON

Consistency index

0.495

Retention index

0.863

**Topology of the SCT with marked nodes**


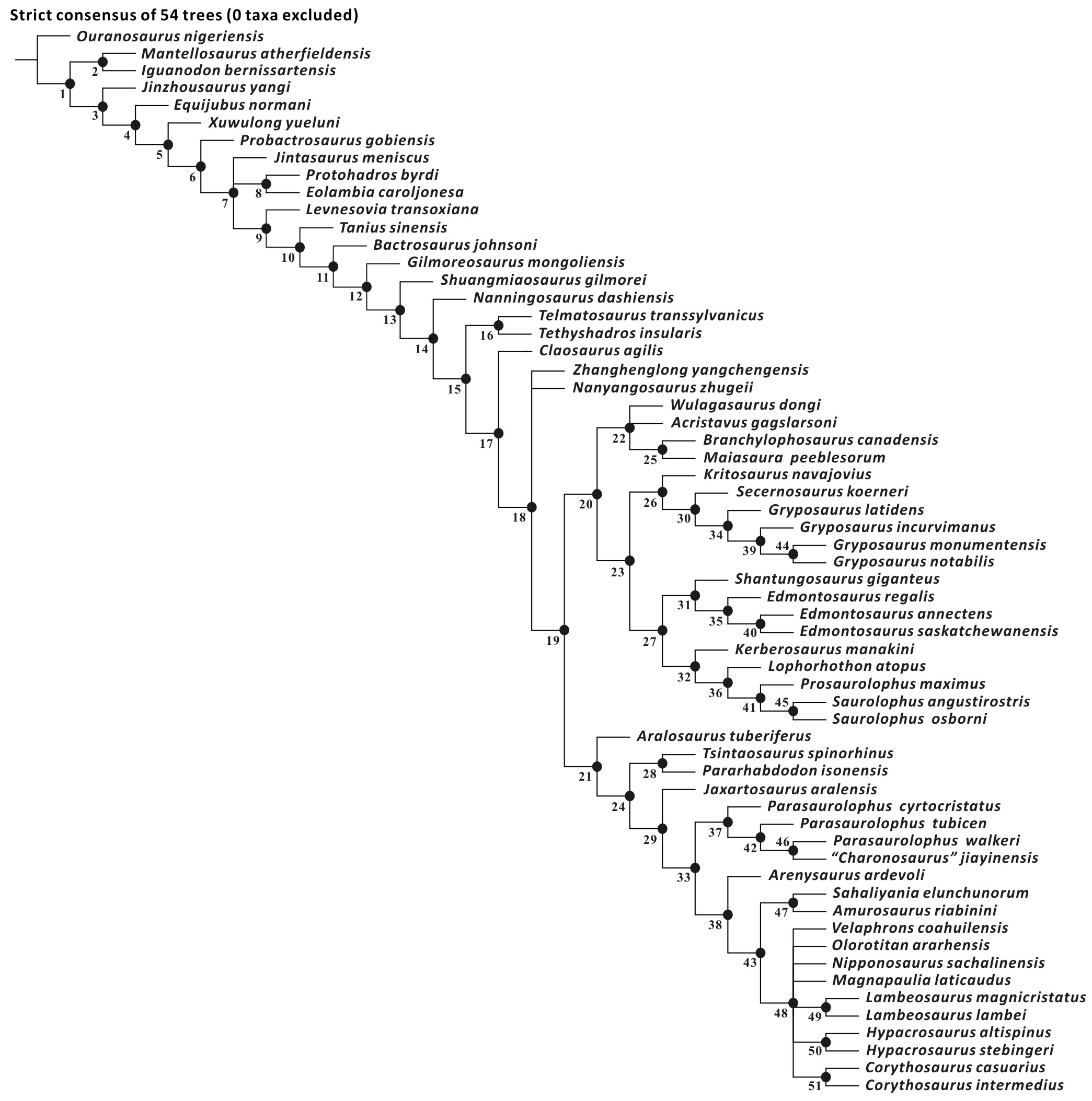


Figure 1. Strict consensus of 54 MPTs recovered from the phylogenetic analysis of Hadrosauroidea using the maximum parsimonious method, with the labeled node numbers.

**Unambiguous synapomorphies common to all MPTs for the nodes of the SCT**

Synapomorphies common to 55 trees

(Node numbers refer to nodes in consensus)

Node 1:

All trees:

No synapomorphies

Node 2:

All trees:

Char. 27: 1 --> 0

Char. 123: 0 --> 1

Char. 185: 0 --> 1

Char. 233: 1 --> 0

Char. 326: 1 --> 0

Char. 340: 1 --> 0

Node 3:

All trees:

Char. 58: 0 --> 1

Char. 109: 0 --> 1

Char. 149: 0 --> 1

Char. 232: 0 --> 1

Char. 311: 0 --> 1

Node 4:

All trees:

Char. 3: 0 --> 1

Char. 4: 0 --> 1

Char. 45: 0 --> 1

Char. 64: 0 --> 1

Char. 101: 0 --> 1

Char. 127: 0 --> 1

Char. 171: 0 --> 1

Char. 239: 0 --> 1

Char. 300: 1 --> 0

Node 5:

All trees:

Char. 20: 0 --> 1

Char. 57: 0 --> 1

Char. 131: 0 --> 1

Node 6:

All trees:

Char. 6: 0 --> 1

Char. 32: 0 --> 1

Char. 33: 0 --> 1

Char. 35: 0 --> 1

Char. 43: 0 --> 1

Char. 320: 0 --> 1

Node 7:

Some trees:

Char. 11: 0 --> 1

Char. 72: 0 --> 1

Char. 104: 0 --> 1

Char. 114: 0 --> 1

Char. 287: 0 --> 1

Char. 298: 0 --> 1

Char. 319: 0 --> 1

Char. 321: 0 --> 1

Node 8:

Some trees:

Char. 64: 1 --> 0

Char. 123: 0 --> 1

Char. 127: 1 --> 2

Node 9:

All trees:

Char. 186: 0 --> 1

Some trees:

Char. 19: 0 --> 1

Char. 59: 0 --> 1

Char. 128: 0 --> 1

Char. 132: 0 --> 1

Char. 133: 0 --> 1

Char. 246: 0 --> 1

Char. 292: 0 --> 1

Char. 294: 0 --> 1

Char. 334: 0 --> 1

Node 10:

All trees:

No synapomorphies

Node 11:

All trees:

Char. 203: 1 --> 0

Char. 215: 0 --> 1

Char. 260: 0 --> 1

Char. 263: 0 --> 1

Char. 293: 0 --> 1

Char. 332: 0 --> 1

Node 12:

All trees:

Char. 43: 1 --> 0

Char. 113: 0 --> 1

Char. 290: 0 --> 1

Char. 299: 1 --> 2

Node 13:

All trees:

Char. 42: 1 --> 2

Node 14:

All trees:

Char. 50: 0 --> 1

Char. 52: 0 --> 1

Char. 135: 0 --> 1

Char. 136: 0 --> 1

Char. 238: 0 --> 1

Char. 318: 0 --> 1

Node 15:

All trees:

Char. 55: 0 --> 1

Char. 107: 0 --> 1

Char. 112: 1 --> 2

Node 16:

All trees:

Char. 11: 1 --> 0

Char. 23: 1 --> 0

Char. 40: 2 --> 0

Node 17:

All trees:

Char. 3: 1 --> 2

Char. 6: 1 --> 3

Char. 54: 0 --> 1

Char. 114: 1 --> 2

Char. 119: 0 --> 1

Char. 120: 0 --> 2

Char. 122: 0 --> 1

Node 18:

All trees:

Char. 7: 0 --> 1

Char. 111: 0 --> 1

Char. 256: 0 --> 1

Char. 267: 0 --> 1

Char. 272: 0 --> 1

Node 19:

All trees:

Char. 5: 1 --> 2

Char. 16: 0 --> 1

Char. 19: 1 --> 2

Char. 46: 0 --> 1

Char. 48: 0 --> 1

Char. 53: 0 --> 1

Char. 56: 1 --> 2

Char. 104: 1 --> 2

Char. 116: 0 --> 1

Char. 118: 0 --> 1

Char. 126: 0 --> 2

Char. 269: 0 --> 1

Char. 281: 0 --> 1

Node 20:

All trees:

Char. 11: 1 --> 2

Char. 35: 1 --> 2

Char. 64: 1 --> 0

Char. 73: 01 --> 2

Char. 77: 0 --> 1

Char. 102: 0 --> 1

Char. 127: 0 --> 1

Char. 162: 0 --> 1

Char. 200: 0 --> 1

Char. 202: 0 --> 1

Char. 215: 1 --> 0

Char. 255: 0 --> 2

Char. 258: 0 --> 1

Char. 261: 0 --> 1

Char. 263: 1 --> 2

Char. 264: 0 --> 1

Char. 279: 0 --> 1

Char. 308: 0 --> 1

Char. 318: 1 --> 2

Char. 325: 0 --> 1

Char. 328: 0 --> 1

Some trees:

Char. 42: 2 --> 0

Node 21:

All trees:

Char. 95: 0 --> 1

Char. 99: 1 --> 0

Char. 103: 0 --> 1

Char. 130: 0 --> 1

Char. 138: 0 --> 1

Char. 140: 0 --> 1

Char. 229: 0 --> 1

Char. 265: 0 --> 1

Node 22:

Some trees:

Char. 76: 0 --> 1

Char. 110: 0 --> 1

Char. 145: 0 --> 1

Char. 151: 2 --> 1

Char. 158: 1 --> 0

Char. 216: 0 --> 1

Char. 219: 1 --> 2

Char. 232: 1 --> 0

Node 23:

All trees:

Char. 49: 1 --> 2

Char. 98: 0 --> 1

Char. 113: 1 --> 3

Char. 174: 0 --> 1

Char. 316: 1 --> 0

Char. 341: 0 --> 1

Node 24:

All trees:

Char. 83: 0 --> 1

Char. 113: 1 --> 3

Char. 118: 1 --> 2

Char. 131: 1 --> 0

Char. 153: 0 --> 1

Char. 154: 0 --> 1

Char. 157: 0 --> 1

Char. 168: 0 --> 1

Char. 172: 0 --> 1

Char. 196: 0 --> 1

Char. 197: 0 --> 1

Char. 266: 0 --> 1

Char. 269: 1 --> 2

Char. 272: 1 --> 2

Node 25:

All trees:

Char. 134: 1 --> 0

Some trees:

Char. 51: 0 --> 1

Char. 113: 1 --> 2

Char. 139: 0 --> 1

Char. 148: 0 --> 2

Char. 176: 0 --> 1

Char. 288: 0 --> 1

Node 26:

All trees:

Char. 99: 1 --> 2

Char. 120: 2 --> 1

Char. 126: 2 --> 1

Char. 127: 1 --> 2

Char. 134: 1 --> 0

Char. 143: 0 --> 2

Char. 150: 0 --> 1

Char. 187: 1 --> 0

Char. 225: 1 --> 0

Char. 226: 0 --> 1

Some trees:

Char. 38: 1 --> 0

Node 27:

All trees:

Char. 16: 1 --> 2

Char. 40: 2 --> 0

Char. 47: 0 --> 1

Char. 63: 1 --> 2

Char. 118: 1 --> 0

Char. 123: 12 --> 0

Char. 145: 0 --> 2

Char. 146: 1 --> 2

Char. 148: 0 --> 2

Char. 162: 1 --> 2

Char. 163: 0 --> 1

Char. 227: 0 --> 1

Char. 228: 0 --> 1

Char. 273: 0 --> 1

Char. 309: 3 --> 2

Char. 320: 1 --> 2

Node 28:

All trees:

Char. 40: 2 --> 0

Char. 45: 1 --> 0

Node 29:

All trees:

Char. 192: 0 --> 1

Char. 193: 0 --> 1

Char. 194: 0 --> 1

Node 30:

All trees:

Char. 101: 2 --> 3

Char. 149: 1 --> 2

Some trees:

Char. 42: 0 --> 1

Node 31:

All trees:

Char. 3: 2 --> 3

Char. 152: 1 --> 0

Char. 164: 1 --> 0

Char. 182: 1 --> 2

Char. 204: 0 --> 1

Char. 232: 1 --> 0

Node 32:

All trees:

No synapomorphies

Node 33:

All trees:

Char. 179: 0 --> 1

Char. 188: 0 --> 1

Char. 226: 0 --> 2

Node 34:

All trees:

Char. 17: 1 --> 0

Char. 38: 0 --> 2

Char. 300: 1 --> 2

Node 35:

All trees:

Char. 27: 2 --> 1

Char. 48: 1 --> 2

Char. 183: 0 --> 1

Char. 327: 1 --> 0

Node 36:

All trees:

Char. 133: 1 --> 2

Char. 163: 1 --> 2

Node 37:

All trees:

Char. 86: 0 --> 1

Char. 152: 1 --> 2

Char. 193: 1 --> 2

Char. 218: 0 --> 1

Char. 261: 0 --> 1

Char. 264: 0 --> 1

Char. 329: 0 --> 1

Char. 337: 0 --> 1

Node 38:

All trees:

Char. 189: 2 --> 1

Node 39:

All trees:

Char. 73: 2 --> 1

Node 40:

All trees:

Char. 230: 0 --> 1

Char. 231: 1 --> 0

Char. 271: 2 --> 1

Char. 341: 1 --> 0

Node 41:

All trees:

Char. 139: 0 --> 2

Char. 140: 0 --> 1

Char. 197: 0 --> 1

Char. 198: 0 --> 1

Char. 343: 0 --> 1

Char. 344: 0 --> 1

Node 42:

All trees:

Char. 5: 2 --> 3

Char. 11: 1 --> 2

Char. 194: 1 --> 3

Node 43:

All trees:

Char. 180: 0 --> 1

Char. 257: 0 --> 1

Char. 258: 0 --> 2

Node 44:

All trees:

Char. 63: 1 --> 2

Char. 150: 1 --> 2

Node 45:

All trees:

Char. 73: 2 --> 1

Char. 85: 0 --> 1

Char. 89: 0 --> 1

Char. 143: 6 --> 4

Char. 174: 1 --> 0

Char. 179: 0 --> 1

Char. 191: 1 --> 2

Char. 192: 0 --> 1

Char. 194: 0 --> 2

Char. 199: 0 --> 2

Char. 200: 1 --> 2

Char. 241: 0 --> 1

Char. 255: 2 --> 1

Char. 270: 1 --> 2

Char. 274: 1 --> 0

Char. 336: 0 --> 1

Node 46:

All trees:

No synapomorphies

Node 47:

All trees:

Char. 11: 1 --> 2

Char. 41: 1 --> 2

Char. 62: 0 --> 1

Char. 205: 1 --> 3

Some trees:

Char. 92: 0 --> 1

Node 48:

All trees:

Char. 39: 0 --> 1

Char. 42: 2 --> 1

Char. 248: 0 --> 1

Some trees:

Char. 40: 2 --> 1

Char. 63: 1 --> 2

Char. 127: 0 --> 1

Char. 133: 1 --> 0

Char. 141: 4 --> 3

Char. 145: 4 --> 5

Char. 217: 0 --> 1

Char. 288: 0 --> 1

Node 49:

All trees:

Char. 317: 0 --> 1

Some trees:

Char. 27: 3 --> 2

Char. 29: 1 --> 0

Char. 35: 1 --> 2

Char. 84: 2 --> 3

Char. 88: 01 --> 2

Char. 90: 0 --> 1

Char. 92: 0 --> 1

Char. 93: 1 --> 2

Char. 98: 1 --> 0

Char. 133: 0 --> 1

Char. 141: 3 --> 4

Char. 145: 5 --> 4

Char. 169: 1 --> 2

Char. 220: 0 --> 1

Node 50:

All trees:

Char. 159: 1 --> 2

Char. 243: 0 --> 1

Char. 341: 0 --> 1

Some trees:

Char. 5: 2 --> 3

Char. 67: 1 --> 0

Char. 186: 0 --> 1

Char. 245: 1 --> 2

Node 51:

All trees:

Char. 24: 1 --> 0

Some trees:

Char. 92: 0 --> 1

Char. 218: 0 --> 1

Char. 219: 1 --> 0

Char. 261: 0 --> 1

Char. 327: 1 --> 2

**Unambiguous synapomorphies of the nodes in the SCT**

Tree 54:

Node 1:

No synapomorphies

Node 2:

Char. 27: 1 --> 0

Char. 123: 0 --> 1

Char. 185: 0 --> 1

Char. 233: 1 --> 0

Char. 326: 1 --> 0

Char. 340: 1 --> 0

Node 3:

Char. 58: 0 --> 1

Char. 109: 0 --> 1

Char. 149: 0 --> 1

Char. 232: 0 --> 1

Char. 311: 0 --> 1

Node 4:

Char. 3: 0 --> 1

Char. 4: 0 --> 1

Char. 45: 0 --> 1

Char. 64: 0 --> 1

Char. 101: 0 --> 1

Char. 127: 0 --> 1

Char. 171: 0 --> 1

Char. 239: 0 --> 1

Char. 300: 1 --> 0

Node 5:

Char. 20: 0 --> 1

Char. 57: 0 --> 1

Char. 131: 0 --> 1

Node 6:

Char. 6: 0 --> 1

Char. 32: 0 --> 1

Char. 33: 0 --> 1

Char. 35: 0 --> 1

Char. 43: 0 --> 1

Char. 320: 0 --> 1

Node 7:

Char. 11: 0 --> 1

Char. 72: 0 --> 1

Char. 104: 0 --> 1

Char. 114: 0 --> 1

Char. 287: 0 --> 1

Char. 298: 0 --> 1

Char. 319: 0 --> 1

Char. 321: 0 --> 1

Node 8:

Char. 64: 1 --> 0

Char. 123: 0 --> 1

Char. 127: 1 --> 2

Node 9:

Char. 19: 0 --> 1

Char. 59: 0 --> 1

Char. 128: 0 --> 1

Char. 132: 0 --> 1

Char. 133: 0 --> 1

Char. 187: 0 --> 1

Char. 246: 0 --> 1

Char. 292: 0 --> 1

Char. 294: 0 --> 1

Char. 334: 0 --> 1

Node 10:

No synapomorphies

Node 11:

Char. 203: 1 --> 0

Char. 215: 0 --> 1

Char. 260: 0 --> 1

Char. 263: 0 --> 1

Char. 293: 0 --> 1

Char. 332: 0 --> 1

Node 12:

Char. 43: 1 --> 0

Char. 113: 0 --> 1

Char. 290: 0 --> 1

Char. 299: 1 --> 2

Node 13:

Char. 42: 1 --> 2

Node 14:

Char. 50: 0 --> 1

Char. 52: 0 --> 1

Char. 135: 0 --> 1

Char. 136: 0 --> 1

Char. 238: 0 --> 1

Char. 318: 0 --> 1

Node 15:

Char. 55: 0 --> 1

Char. 107: 0 --> 1

Char. 112: 1 --> 2

Node 16:

Char. 11: 1 --> 0

Char. 23: 1 --> 0

Char. 40: 2 --> 0

Node 17:

Char. 3: 1 --> 2

Char. 6: 1 --> 3

Char. 54: 0 --> 1

Char. 114: 1 --> 2

Char. 119: 0 --> 1

Char. 120: 0 --> 2

Char. 122: 0 --> 1

Node 18:

Char. 7: 0 --> 1

Char. 111: 0 --> 1

Char. 256: 0 --> 1

Char. 267: 0 --> 1

Char. 272: 0 --> 1

Node 19:

Char. 5: 1 --> 2

Char. 16: 0 --> 1

Char. 19: 1 --> 2

Char. 46: 0 --> 1

Char. 48: 0 --> 1

Char. 53: 0 --> 1

Char. 56: 1 --> 2

Char. 104: 1 --> 2

Char. 116: 0 --> 1

Char. 118: 0 --> 1

Char. 126: 0 --> 2

Char. 269: 0 --> 1

Char. 281: 0 --> 1

Node 20:

Char. 11: 1 --> 2

Char. 35: 1 --> 2

Char. 64: 1 --> 0

Char. 73: 01 --> 2

Char. 77: 0 --> 1

Char. 102: 0 --> 1

Char. 127: 0 --> 1

Char. 162: 0 --> 1

Char. 200: 0 --> 1

Char. 202: 0 --> 1

Char. 215: 1 --> 0

Char. 255: 0 --> 2

Char. 258: 0 --> 1

Char. 261: 0 --> 1

Char. 263: 1 --> 2

Char. 264: 0 --> 1

Char. 279: 0 --> 1

Char. 308: 0 --> 1

Char. 318: 1 --> 2

Char. 325: 0 --> 1

Char. 328: 0 --> 1

Node 21:

Char. 95: 0 --> 1

Char. 99: 1 --> 0

Char. 103: 0 --> 1

Char. 130: 0 --> 1

Char. 138: 0 --> 1

Char. 140: 0 --> 1

Char. 229: 0 --> 1

Char. 265: 0 --> 1

Node 22:

Char. 76: 0 --> 1

Char. 110: 0 --> 1

Char. 145: 0 --> 1

Char. 151: 2 --> 1

Char. 158: 1 --> 0

Char. 216: 0 --> 1

Char. 219: 1 --> 2

Char. 232: 1 --> 0

Node 23:

Char. 49: 1 --> 2

Char. 98: 0 --> 1

Char. 113: 1 --> 3

Char. 174: 0 --> 1

Char. 316: 1 --> 0

Char. 341: 0 --> 1

Node 24:

Char. 83: 0 --> 1

Char. 113: 1 --> 3

Char. 118: 1 --> 2

Char. 131: 1 --> 0

Char. 153: 0 --> 1

Char. 154: 0 --> 1

Char. 157: 0 --> 1

Char. 168: 0 --> 1

Char. 172: 0 --> 1

Char. 196: 0 --> 1

Char. 197: 0 --> 1

Char. 266: 0 --> 1

Char. 269: 1 --> 2

Char. 272: 1 --> 2

Node 25:

Char. 51: 0 --> 1

Char. 113: 1 --> 2

Char. 134: 1 --> 0

Char. 139: 0 --> 1

Char. 148: 0 --> 2

Char. 176: 0 --> 1

Char. 288: 0 --> 1

Node 26:

Char. 99: 1 --> 2

Char. 120: 2 --> 1

Char. 126: 2 --> 1

Char. 127: 1 --> 2

Char. 134: 1 --> 0

Char. 143: 0 --> 2

Char. 150: 0 --> 1

Char. 187: 1 --> 0

Char. 225: 1 --> 0

Char. 226: 0 --> 1

Node 27:

Char. 16: 1 --> 2

Char. 40: 2 --> 0

Char. 47: 0 --> 1

Char. 63: 1 --> 2

Char. 118: 1 --> 0

Char. 123: 12 --> 0

Char. 145: 0 --> 2

Char. 146: 1 --> 2

Char. 148: 0 --> 2

Char. 162: 1 --> 2

Char. 163: 0 --> 1

Char. 227: 0 --> 1

Char. 228: 0 --> 1

Char. 273: 0 --> 1

Char. 309: 3 --> 2

Char. 320: 1 --> 2

Node 28:

Char. 40: 2 --> 0

Char. 45: 1 --> 0

Node 29:

Char. 192: 0 --> 1

Char. 193: 0 --> 1

Char. 194: 0 --> 1

Node 30:

Char. 101: 2 --> 3

Char. 149: 1 --> 2

Node 31:

Char. 3: 2 --> 3

Char. 152: 1 --> 0

Char. 164: 1 --> 0

Char. 182: 1 --> 2

Char. 204: 0 --> 1

Char. 232: 1 --> 0

Node 32:

No synapomorphies

Node 33:

Char. 179: 0 --> 1

Char. 188: 0 --> 1

Char. 226: 0 --> 2

Node 34:

Char. 17: 1 --> 0

Char. 38: 0 --> 2

Char. 300: 1 --> 2

Node 35:

Char. 27: 2 --> 1

Char. 48: 1 --> 2

Char. 183: 0 --> 1

Char. 327: 1 --> 0

Node 36:

Char. 133: 1 --> 2

Char. 163: 1 --> 2

Node 37:

Char. 86: 0 --> 1

Char. 152: 1 --> 2

Char. 193: 1 --> 2

Char. 218: 0 --> 1

Char. 261: 0 --> 1

Char. 264: 0 --> 1

Char. 329: 0 --> 1

Char. 337: 0 --> 1

Node 38:

Char. 189: 2 --> 1

Node 39:

Char. 73: 2 --> 1

Node 40:

Char. 230: 0 --> 1

Char. 231: 1 --> 0

Char. 271: 2 --> 1

Char. 341: 1 --> 0

Node 41:

Char. 139: 0 --> 2

Char. 140: 0 --> 1

Char. 197: 0 --> 1

Char. 198: 0 --> 1

Char. 343: 0 --> 1

Char. 344: 0 --> 1

Node 42:

Char. 5: 2 --> 3

Char. 11: 1 --> 2

Char. 194: 1 --> 3

Node 43:

Char. 180: 0 --> 1

Char. 257: 0 --> 1

Char. 258: 0 --> 2

Node 44:

Char. 63: 1 --> 2

Char. 150: 1 --> 2

Node 45:

Char. 73: 2 --> 1

Char. 85: 0 --> 1

Char. 89: 0 --> 1

Char. 143: 6 --> 4

Char. 174: 1 --> 0

Char. 179: 0 --> 1

Char. 191: 1 --> 2

Char. 192: 0 --> 1

Char. 194: 0 --> 2

Char. 199: 0 --> 2

Char. 200: 1 --> 2

Char. 241: 0 --> 1

Char. 255: 2 --> 1

Char. 270: 1 --> 2

Char. 274: 1 --> 0

Char. 336: 0 --> 1

Node 46:

No synapomorphies

Node 47:

Char. 11: 1 --> 2

Char. 41: 1 --> 2

Char. 62: 0 --> 1

Char. 92: 0 --> 1

Char. 205: 1 --> 3

Node 48:

Char. 39: 0 --> 1

Char. 40: 2 --> 1

Char. 42: 2 --> 1

Char. 63: 1 --> 2

Char. 127: 0 --> 1

Char. 133: 1 --> 0

Char. 141: 4 --> 3

Char. 145: 4 --> 5

Char. 217: 0 --> 1

Char. 248: 0 --> 1

Char. 288: 0 --> 1

Node 49:

Char. 27: 3 --> 2

Char. 29: 1 --> 0

Char. 84: 2 --> 3

Char. 88: 1 --> 2

Char. 90: 0 --> 1

Char. 92: 0 --> 1

Char. 93: 1 --> 2

Char. 98: 1 --> 0

Char. 133: 0 --> 1

Char. 141: 3 --> 4

Char. 145: 5 --> 4

Char. 169: 1 --> 2

Char. 220: 0 --> 1

Char. 317: 0 --> 1

Node 50:

Char. 67: 1 --> 0

Char. 159: 1 --> 2

Char. 243: 0 --> 1

Char. 245: 1 --> 2

Char. 341: 0 --> 1

Node 51:

Char. 24: 1 --> 0

Char. 92: 0 --> 1

Char. 218: 0 --> 1

Char. 219: 1 --> 0

Char. 261: 0 --> 1

Char. 327: 1 --> 2


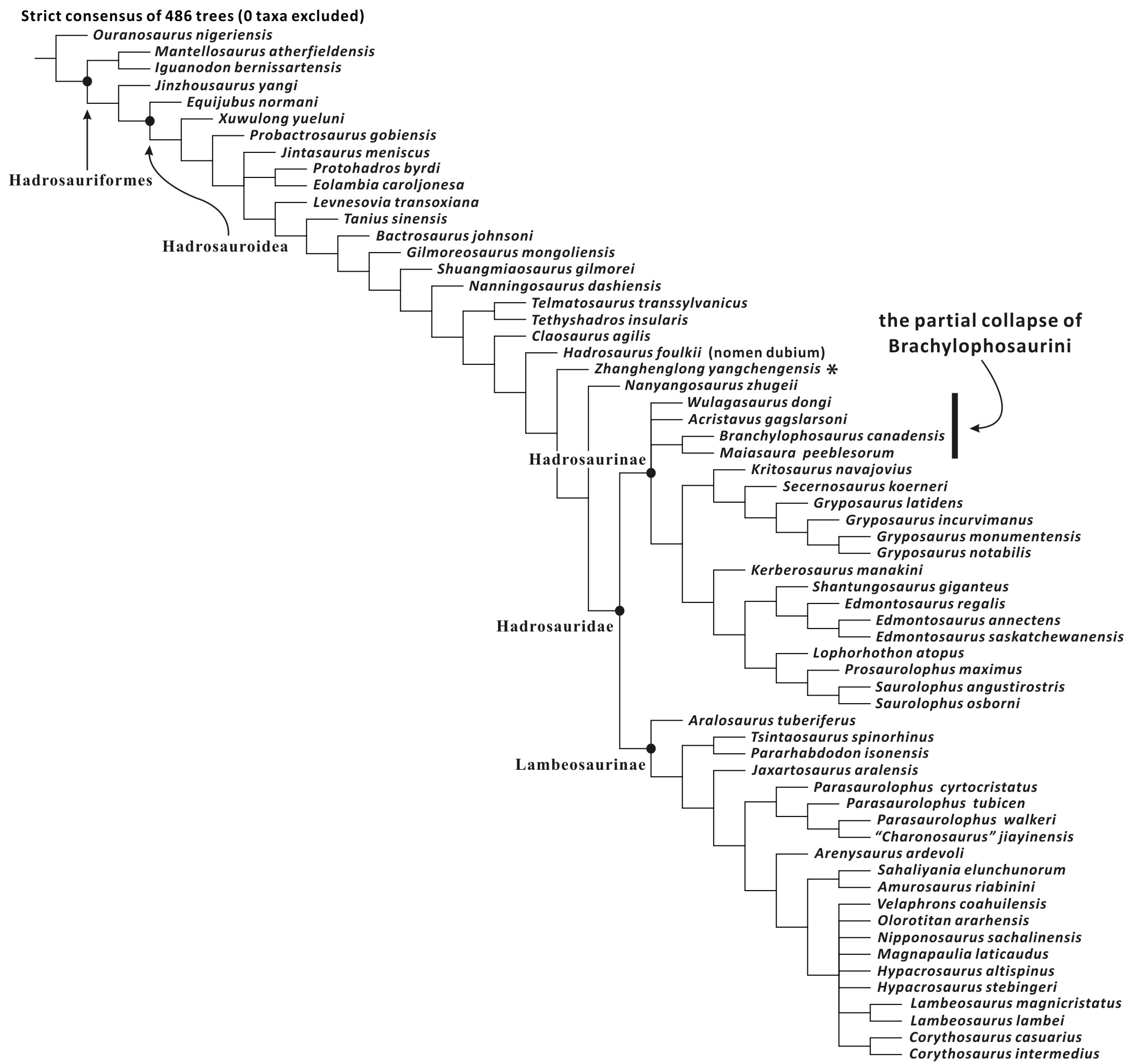


Figure 2. Strict consensus of 486 MPTs recovered from the phylogenetic analysis of Hadrosauroidea using the maximum parsimonious method, with the participation of *Hadrosaurus foulkii*.

Although *Hadrosaurus foulkii* is considered here to be a *nomen dubium*, we still attempted to add this taxon to the current phylogenetic analysis of Hadrosauroidea. The maximum parsimony analysis with the participation of *H. foulkii* has yielded 486 MPTs. Compared with the tree topology shown in Fig. 1 (also see text, Fig. 13), the phylogenetic position of *Zhanghenglong yangchengensis* doesn’t significantly alter in the strict consensus of the 486 MPTs (Fig. 2), where the taxon is placed as the sister group to the clade consisting of *Nanyangosaurus zhugeii* and Hadrosauridae, and is depicted as more derived than *H. foulkii*. Interestingly, the participation of *H. foulkii* in the phylogenetic analysis results in the collapse of the tribe Brachylophosaurini (Fig. 2). This tribe is recovered as the least inclusive clade containing *Brachyolophosaurus canadensis* and *Wulagasaurus dongi* in the tree topology of the text (Fig. 1).

**Original information showing the concrete operation of the phylogenetic analysis of Hadrosauroidea including *Hadrosaurus foulkii* in the user interface of TNT**

Reading from C:\Users\Hai Xing\Desktop\Zhanghenglongnew.ss

'Exported from NEXUS Data Editor'

Matrix (346 × 62, 16 states). Memory required for data: 0.31 Mbytes

Space for 10000 trees in memory

Not collapsing trees temporarily for consensus calculation

Ignore unshared taxa is ON

Outgroup is taxon 9 – Ouranosaurus_nigeriensis

0 trees in memory

Random seed is 1

Repl. Algor. Tree Score Best Score Time Rearrangs.

1000 TBR 486 of 486 ------ 1012 0:00:44 749,575,743

Completed 1000 random addition sequences.

Total rearrangements examined: 749,575,743.

Best score hit 1000 times out of 1000.

Best score (TBR): 1012. 486 trees retained.

Time 44.51 secs.

Strict consensus of 486 trees (0 taxa excluded) calculated, as tree 486

Time 0.02 secs.

Tree lengths

1012 (MPTs) 1052 (SCT)

Time 0.06 secs.

Reading from E:\TNT\WSTATS.RUN

Macro language is ON

Macros: 42.7 Kb in use, 59.6 Kb free

Report is ON

Consistency index

0.458

Retention index

0.838


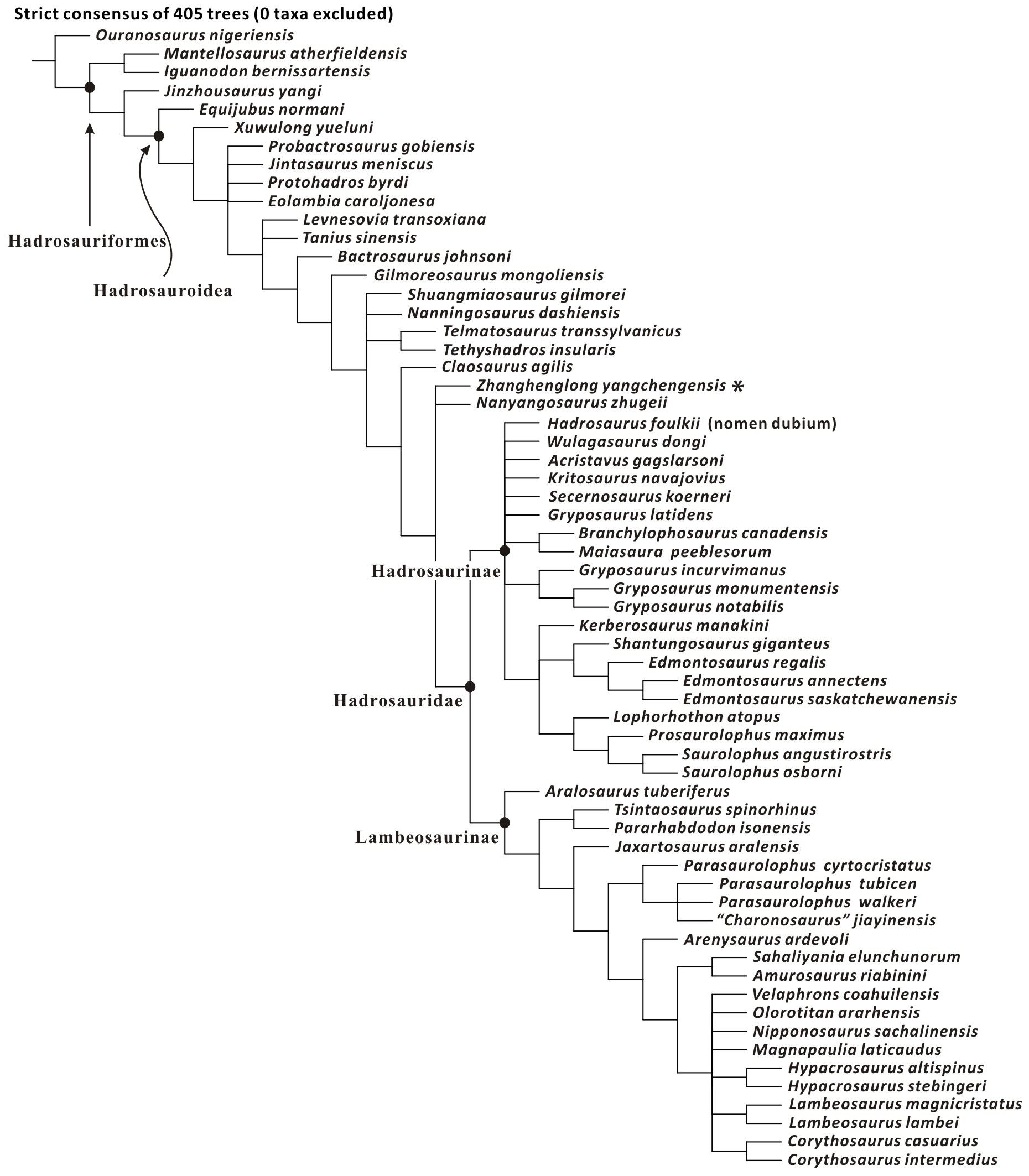


Figure 3. Strict consensus of 405 MPTs recovered from the maximum parsimonious analysis of Hadrosauroidea, with the participation of *Hadrosaurus foulkii* (the characters associated with the laterodistal corner of the deltopectoral crest were coded as polymorphic for *Brachylophosaurus*, *Shantungosaurus*, and *Edmontosaurus*).

Given that the wide arcuate laterodistal corner of the deltopectoral crest of the humerus possibly disturbs the accurate coding of the dataset of *Hadrosaurus foulkii* for the phylogenetic analysis, and is also seen in some non-lambeosaurine hadrosaurid taxa (see Supporting Information S4), we conducted another analysis, in which all the characters associated with the laterodistal corner of the deltopectoral crest (namely characters 267, 269, 271, and 272) were coded as polymorphic for *Brachylophosaurus*, *Shantungosaurus*, and *Edmontosaurus*. The result of the alternate cladistic analysis indicates that *H. foulkii* is definitely a member of Hadrosaurinae that has a large, unresolved polytomy at the base of the clade (Fig. 3). Therefore, we argue that the coding of the four preceding characters plays an important role in placing *H. foulkii* outside of Hadrosauridae (Fig. 2). Nevertheless, it is very difficult to exactly evaluate whether the wide arcuate laterodistal corner of the deltopectoral crest represents the common, natural condition among the adult individuals of *H. foulkii* (regardless of the validity of the taxon).
